# Global, regional, and national burden of rheumatoid arthritis, 1990–2020, and projections to 2050: a systematic analysis of the Global Burden of Disease Study 2021

**DOI:** 10.1016/S2665-9913(23)00211-4

**Published:** 2023-09-25

**Authors:** GBD 2021 Rheumatoid Arthritis Collaborators, Rachel J Black, Marita Cross, Lydia M Haile, Garland T Culbreth, Jaimie D Steinmetz, Hailey Hagins, Jacek A Kopec, Peter M Brooks, Anthony D Woolf, Kanyin Liane Ong, Deborah R Kopansky-Giles, Karsten E Dreinhoefer, Neil Betteridge, Amirali Aali, Mitra Abbasifard, Mohsen Abbasi-Kangevari, Ame Mehadi Abdurehman, Aidin Abedi, Hassan Abidi, Richard Gyan Aboagye, Hassan Abolhassani, Eman Abu-Gharbieh, Ahmed Abu-Zaid, Kidist Adamu, Isaac Yeboah Addo, Miracle Ayomikun Adesina, Qorinah Estiningtyas Sakilah Adnani, Muhammad Sohail Afzal, Ayman Ahmed, Janardhana P Aithala, Meisam Akhlaghdoust, Astawus Alemayehu, Saba Alvand, Nelson J Alvis-Zakzuk, Hubert Amu, Benny Antony, Jalal Arabloo, Aleksandr Y Aravkin, Judie Arulappan, Tahira Ashraf, Seyyed Shamsadin Athari, Sina Azadnajafabad, Alaa Badawi, Nayereh Baghcheghi, Atif Amin Baig, Asaminew Birhanu Balta, Maciej Banach, Palash Chandra Banik, Amadou Barrow, Azadeh Bashiri, Lindsay M Bearne, Alehegn Bekele, Isabela M Bensenor, Alemshet Yirga Berhie, Akshaya Srikanth Bhagavathula, Pankaj Bhardwaj, Ajay Nagesh Bhat, Vijayalakshmi S Bhojaraja, Saeid Bitaraf, Belay Boda Abule Bodicha, João Silva Botelho, Andrew M Briggs, Rachelle Buchbinder, Carlos A Castañeda-Orjuela, Periklis Charalampous, Vijay Kumar Chattu, Kaleb Coberly, Natália Cruz-Martins, Omid Dadras, Xiaochen Dai, Katie de Luca, Fikadu Nugusu Dessalegn, Gashaw Dessie, Meghnath Dhimal, Lankamo Ena Digesa, Mengistie Diress, Paul Narh Doku, Hisham Atan Edinur, Michael Ekholuenetale, Muhammed Elhadi, Yasser Mohamed El-Sherbiny, Farshid Etaee, Rana Ezzeddini, Shahriar Faghani, Irina Filip, Florian Fischer, Takeshi Fukumoto, Balasankar Ganesan, Mathewos Alemu Gebremichael, Urge Gerema, Motuma Erena Getachew, Ahmad Ghashghaee, Tiffany K Gill, Bhawna Gupta, Sapna Gupta, Veer Bala Gupta, Vivek Kumar Gupta, Rabih Halwani, Md Abdul Hannan, Shafiul Haque, Netanja I Harlianto, Mehdi Harorani, Ahmed I Hasaballah, Mohammed Bheser Hassen, Simon I Hay, Khezar Hayat, Golnaz Heidari, Kamal Hezam, Catherine L Hill, Yuta Hiraike, Nobuyuki Horita, Amir Human Hoveidaei, Alexander Kevin Hsiao, Evelyn Hsieh, Salman Hussain, Ivo Iavicoli, Irena M Ilic, Sheikh Mohammed Shariful Islam, Nahlah Elkudssiah Ismail, Masao Iwagami, Mihajlo Jakovljevic, Chinmay T Jani, Jayakumar Jeganathan, Nitin Joseph, Vidya Kadashetti, Himal Kandel, Tesfaye K Kanko, Ibraheem M Karaye, Himanshu Khajuria, Md Jobair Khan, Moien AB Khan, Javad Khanali, Moawiah Mohammad Khatatbeh, Jagdish Khubchandani, Yun Jin Kim, Adnan Kisa, Ali-Asghar Kolahi, Farzad Kompani, Hamid Reza Koohestani, Ai Koyanagi, Kewal Krishan, Mohammed Kuddus, Narinder Kumar, Ambily Kuttikkattu, Bagher Larijani, Stephen S Lim, Justin Lo, Vanessa Sintra Machado, Preetam Bhalchandra Mahajan, Azeem Majeed, Elaheh Malakan Rad, Ahmad Azam Malik, Mohammad Ali Mansournia, Elezebeth Mathews, José João Mendes, Alexios-Fotios A Mentis, Mohamed Kamal Mesregah, Tomislav Mestrovic, Seyed Peyman Mirghaderi, Erkin M Mirrakhimov, Awoke Misganaw, Ashraf Mohamadkhani, Shafiu Mohammed, Ali H Mokdad, Md Moniruzzaman, Ahmed Al Montasir, Getaneh Baye Mulu, Efrén Murillo-Zamora, Christopher J L Murray, Ghulam Mustafa, Mohsen Naghavi, Tapas Sadasivan Nair, Atta Abbas Naqvi, Zuhair S Natto, Biswa Prakash Nayak, Subas Neupane, Cuong Tat Nguyen, Robina Khan Niazi, Ogochukwu Janet Nzoputam, In-Hwan Oh, Hassan Okati-Aliabad, Osaretin Christabel Okonji, Isaac Iyinoluwa Olufadewa, Mayowa O Owolabi, Kevin Pacheco-Barrios, Jagadish Rao Padubidri, Jay Patel, Aslam Ramjan Pathan, Shrikant Pawar, Paolo Pedersini, Arokiasamy Perianayagam, Ionela-Roxana Petcu, Ibrahim Qattea, Amir Radfar, Alireza Rafiei, Mohammad Hifz Ur Rahman, Vahid Rahmanian, Vahid Rashedi, Mohammad-Mahdi Rashidi, Zubair Ahmed Ratan, Salman Rawaf, Mohammad Sadegh Razeghinia, Elrashdy Moustafa Mohamed Redwan, Andre M N Renzaho, Nazila Rezaei, Nima Rezaei, Abanoub Riad, Aly M A Saad, Basema Saddik, Umar Saeed, Azam Safary, Maryam Sahebazzamani, Amirhossein Sahebkar, Harihar Sahoo, Amir Salek Farrokhi, Muhammad Arif Nadeem Saqib, Allen Seylani, Saeed Shahabi, Masood Ali Shaikh, Bereket Beyene Shashamo, Adithi Shetty, Jeevan K Shetty, Mika Shigematsu, Velizar Shivarov, Parnian Shobeiri, Migbar Mekonnen Sibhat, Ehsan Sinaei, Ambrish Singh, Jasvinder A Singh, Paramdeep Singh, Surjit Singh, Md Shahjahan Siraj, Anna Aleksandrovna Skryabina, Helen Slater, Amanda E Smith, Yonatan Solomon, Mohammad Sadegh Soltani-Zangbar, Mohammad Tabish, Ker-Kan Tan, Nathan Y Tat, Arash Tehrani-Banihashemi, Samar Tharwat, Marcos Roberto Tovani-Palone, Biruk Shalmeno Tusa, Sahel Valadan Tahbaz, Pascual R Valdez, Rohollah Valizadeh, Siavash Vaziri, Stein Emil Vollset, Ai-Min Wu, Dereje Y Yada, Sisay Shewasinad Yehualashet, Naohiro Yonemoto, Yuyi You, Ismaeel Yunusa, Moein Zangiabadian, Iman Zare, Armin Zarrintan, Zhi-Jiang Zhang, Chenwen Zhong, Mohammad Zoladl, Theo Vos, Lyn M March

## Abstract

**Background:**

Rheumatoid arthritis is a chronic autoimmune inflammatory disease associated with disability and premature death. Up-to-date estimates of the burden of rheumatoid arthritis are required for health-care planning, resource allocation, and prevention. As part of the Global Burden of Diseases, Injuries, and Risk Factors Study (GBD) 2021, we provide updated estimates of the prevalence of rheumatoid arthritis and its associated deaths and disability-adjusted life-years (DALYs) by age, sex, year, and location, with forecasted prevalence to 2050.

**Methods:**

Rheumatoid arthritis prevalence was estimated in 204 countries and territories from 1990 to 2020 using Bayesian meta-regression models and data from population-based studies and medical claims data (98 prevalence and 25 incidence studies). Mortality was estimated from vital registration data with the Cause of Death Ensemble model (CODEm). Years of life lost (YLL) were calculated with use of standard GBD lifetables, and years lived with disability (YLDs) were estimated from prevalence, a meta-analysed distribution of rheumatoid arthritis severity, and disability weights. DALYs were calculated by summing YLLs and YLDs. Smoking was the only risk factor analysed. Rheumatoid arthritis prevalence was forecast to 2050 by logistic regression with Socio-Demographic Index as a predictor, then multiplying by projected population estimates.

**Findings:**

In 2020, an estimated 17·6 million (95% uncertainty interval 15·8–20·3) people had rheumatoid arthritis worldwide. The age-standardised global prevalence rate was 208·8 cases (186·8–241·1) per 100 000 population, representing a 14·1% (12·7–15·4) increase since 1990. Prevalence was higher in females (age-standardised female-to-male prevalence ratio 2·45 [2·40–2·47]). The age-standardised death rate was 0·47 (0·41–0·54) per 100 000 population (38 300 global deaths [33 500–44 000]), a 23·8% (17·5–29·3) decrease from 1990 to 2020. The 2020 DALY count was 3 060 000 (2 320 000–3 860 000), with an age-standardised DALY rate of 36·4 (27·6–45·9) per 100 000 population. YLDs accounted for 76·4% (68·3–81·0) of DALYs. Smoking risk attribution for rheumatoid arthritis DALYs was 7·1% (3·6–10·3). We forecast that 31·7 million (25·8–39·0) individuals will be living with rheumatoid arthritis worldwide by 2050.

**Interpretation:**

Rheumatoid arthritis mortality has decreased globally over the past three decades. Global age-standardised prevalence rate and YLDs have increased over the same period, and the number of cases is projected to continue to increase to the year 2050. Improved access to early diagnosis and treatment of rheumatoid arthritis globally is required to reduce the future burden of the disease.

**Funding:**

Bill & Melinda Gates Foundation, Institute of Bone and Joint Research, and Global Alliance for Musculoskeletal Health.

## Introduction

Rheumatoid arthritis is a chronic autoimmune inflammatory disease that presents as a symmetrical polyarthritis characterised by joint pain, swelling, and stiffness. It can affect any synovial joint in the body, most commonly starting in the small joints of the hands and feet, with the potential to impact every aspect of daily living. High levels of inflammation are associated with fatigue and impairment of participation in occupational, recreational, and societal roles. Rheumatoid arthritis can affect men, women, and children at any age, but is 2–3 times more likely to occur in women and is more common with increasing age, with onset most often occurring at 60–70 years of age.^1^ Without adequate treatment, the disease can lead to progressive joint destruction and deformity, causing long-term disability, chronic pain, and premature death.

Treatment for rheumatoid arthritis has improved considerably over time, leading to better health outcomes. However, access to treatment varies globally and substantial inequities exist.^2^ The optimal management of rheumatoid arthritis involves early diagnosis within a 3-month window of opportunity and treatment with conventional synthetic disease-modifying antirheumatic drugs (DMARDs), using a treat-to-target management approach in which treatment is intensified according to disease activity measures until a state of low disease activity or disease remission is reached.^3^, ^4^ The addition of, or switch to, biological DMARDs or targeted synthetic DMARDs can be considered if there is severe disease or ongoing disease activity with first-line therapies.^4^, ^5^ However, biological and targeted synthetic DMARDs are expensive and access to treatment varies globally.^6^, ^7^ Methotrexate is a low-cost conventional synthetic DMARD and, when started early at effective doses, can control disease activity and minimise long-term disability and mortality. When diagnosis and access to specialist care is delayed, people with rheumatoid arthritis are more likely to have both short-term and long-term disability.^8^

**Research in context**

**Evidence before this study**

The Global Burden of Diseases, Injuries, and Risk Factors Study (GBD) is the only source of global, regional, and country estimates of rheumatoid arthritis burden over time. An initial extensive systematic review of rheumatoid arthritis prevalence globally was done for GBD 2010 and updated for GBD 2017. We searched PubMed for population-based epidemiological studies of rheumatoid arthritis between 1980 and 2017 using the search terms (“Arthritis, Rheumatoid”[Mesh] OR arthrit*) AND (prevalen*[Mesh] OR inciden*[Mesh]) AND (“2017/12/18”[PDAT] : “2019/10/18[PDAT]). Additional studies encountered opportunistically during data review were added for GBD 2015, 2016, and 2019 and six additional studies were added for GBD 2021. In addition, health insurance claims data for 2000, 2010–2012, and 2014–2016 from the USA by state, and for 2016 from Taiwan were included for GBD 2021. To date, there is no published projection to 2050 of the global prevalence of rheumatoid arthritis.

**Added value of this study**

This work provides updated fatal and non-fatal estimates of the global burden of rheumatoid arthritis to 2020. The 2021 iteration of GBD includes new statistical methods to adjust for case definition and the incorporation of modelled excess mortality data into non-fatal models to improve consistency between prevalence and death estimates, and to more accurately capture trends based on health-care access. We report a decrease of 23·8% in the age-standardised global rate of deaths due to rheumatoid arthritis between 1990 and 2020. Additionally, for the first time, we provide forecasted prevalence estimates at the global and regional levels to 2050. We forecast an 80·2% (63·3–92·1) increase to reach 31·7 million people living with rheumatoid arthritis globally by 2050.

**Implications of all the available evidence**

Rheumatoid arthritis continues to affect women more than men, and, in keeping with other autoimmune diseases, age-standardised rheumatoid arthritis prevalence is greater in high-income than in low-income and middle-income countries. However, primary country-level data from low-income and middle-income regions are sparse. Outcomes in rheumatoid arthritis, including severity of disability and mortality, are improved by early diagnosis and access to effective disease-modifying antirheumatic drug (DMARD) therapy, including affordable conventional synthetic DMARDs, such as methotrexate, along with the more expensive biological DMARDs. The decrease in mortality seen over time was greatest in high-income countries which is in keeping with the premise that early access to effective treatment is common in those countries but much less accessible in low-income and middle-income countries. There is a growing body of evidence to suggest that a range of risk factors contribute to the development and progression of rheumatoid arthritis, which might, in part, be preventable; however, only smoking has been addressed as a risk factor in GBD analyses. Further epidemiological studies in low-income and middle-income countries addressing prevalence, mortality, disability impact, and severity distribution are needed. For all regions, a greater focus on modifiable risk factors and access to treatment is needed to both support strategies for rheumatoid arthritis prevention and to enable more accurate comparisons and health policy responses to be made in the future. The estimated increase in number of cases by 2050 is substantial and health-care planning that targets treatment with a particular focus on sex, given the higher prevalence and incidence in females, is warranted. Early access to currently available cost-effective rheumatoid arthritis treatments is required to limit this burden globally.

The global burden of rheumatoid arthritis was first reported in 1990 as part of the initial Global Burden of Diseases, Injuries, and Risk Factors Study (GBD) based on limited data sources.^9^ These data were comprehensively updated for GBD 2010 and were reported in a separate report on the global burden of rheumatoid arthritis for the first time in 2014,^10^ with subsequent updates and expansion in the ongoing iterations of GBD.^11^ Up-to-date measures of rheumatoid arthritis disease burden are required to inform health-care planning and resource allocation, and GBD 2021 provides updated data on rheumatoid arthritis prevalence, mortality (years of life lost [YLL]), years lived with disability (YLD), and disability-adjusted life-years (DALYs). In addition to current measures, estimates of future disease burden are important to a wide range of groups, including, clinicians, researchers, policy makers, and non-government agencies. While industry-driven epidemiological reports have forecasted rheumatoid arthritis across seven to eight countries,^12^, ^13^ to date there has been no broader global forecasting to estimate the future burden of this disease.

The aim of this study was to examine the data from GBD 2021 to provide an updated analysis of rheumatoid arthritis burden (fatal and non-fatal estimates) by age, sex, location, and year. In addition, this study sought to report on smoking as a risk factor for rheumatoid arthritis and to forecast rheumatoid arthritis prevalence to 2050.

## Methods

### Overview

GBD 2021 is a systematic analysis of health loss produced by 369 diseases, injuries, risk factors, impairments, and causes of death. GBD 2021 estimated rheumatoid arthritis mortality, incidence, prevalence, and associated disability by year, age, and sex for 204 countries and territories, using a Bayesian meta-regression tool, DisMod-MR 2.1. Data are reported by country, region, and super-region, with super-regions based on epidemiological similarity and geographical closeness. GBD estimated rheumatoid arthritis prevalence in all countries. For most disease models in GBD, input data were not available for every location where we estimated prevalence. In these cases, prevalence estimates in DisMod-MR 2.1 were made through two main mechanisms: (1) an analytical cascade with initial models at more aggregate levels (global, super-region, and region), with information from such models passed as priors to models at the geographical level below; and (2) predictive covariates. In regions with no data, estimates were informed by super-region priors. GBD adheres to the GATHER statement.^14^ This Article was produced as part of the GBD Collaborator Network and in accordance with the GBD protocol.

### Input data

An initial systematic review of rheumatoid arthritis incidence and prevalence in population-representative data sources throughout the world was done for GBD 2010.^10^ The systematic review was updated for GBD 2017,^11^ with six additional studies encountered opportunistically during data review added for GBD 2021 (appendix p 2). In addition, health insurance claims data from the USA for 2000, 2010–12, and 2014–16 by state and claims data from Taiwan for 2016 were included (International Classification of Diseases codes listed in the appendix [p 2]). Each data source was given a unique identifier and catalogued in the Global Health Data Exchange.

### Case definition

The reference case definition for rheumatoid arthritis was based on the 1987 American College of Rheumatology (ACR) classification criteria,^15^ as the majority of population-based data available used this case definition. These criteria stipulate that rheumatoid arthritis is defined by the presence of four of seven criteria: morning stiffness; arthritis of three or more joint areas; symmetric arthritis; arthritis of hand joints; rheumatoid nodules; serum rheumatoid factor; and radiographical changes. The first four criteria must be present for at least 6 weeks to fulfil the case definition.

Data sources using diagnostic criteria other than the reference criteria, such as the 2010 ACR–European League Against Rheumatism (EULAR) criteria,^16^ were adjusted using a meta-regression tool, MR-BRT (Meta-Regression–Bayesian Regularised Trimmed), as described elsewhere.^17^ Claims data from the USA from 2010 onward and from Taiwan were additionally treated as reference case definition data, as it was assumed that most cases of rheumatoid arthritis would intersect with the health system in these countries. Adjustment factors were derived by pairing data sources with different case definitions by age, sex, year, and location, then running an MR-BRT model meta-analysis on the logit difference between the prevalence of alternative and reference case definitions (appendix p 3). After adjusting data for case definition, data were considered to be outliers if they had an age-standardised mean of two or more median absolute deviations above the median by sex, year, and location.

### Cause of death modelling

Data from vital registration systems were used to estimate mortality due to rheumatoid arthritis. The standard Cause of Death Ensemble model (CODEm), a highly automated tool that selects an ensemble of model types and predictive covariates based on out-of-sample predictive validity, was used to estimate deaths due to rheumatoid arthritis by location, year, age, and sex (appendix pp 5–6). Mortality rates due to rheumatoid arthritis and all other causes were scaled to all-cause mortality estimated from demographic data sources, mainly vital registration systems, surveys, and censuses. YLLs were calculated by multiplying the estimated number of deaths due to rheumatoid arthritis in each age group by the remaining life expectancy, as derived from the standard GBD life table.^17^

### Data processing and disease modelling

Before fitting models, prevalence data reported with wide age ranges and male and female sexes combined were split by age and sex. For data sources that reported prevalence by age and sex separately, the ratio of male-to-female prevalence was applied to age-specific datapoints to split into age-specific and sex-specific datapoints. Subsequently, we ran an MR-BRT model on the log-transformed ratio of male-to-female prevalence for sex-specific data sources, then used the pooled sex ratio to split remaining combined sex datapoints into sex-specific data. For data sources that reported estimates across age groups spanning 25 years or more, we applied the global prevalence age pattern estimated by DisMod-MR 2.1 in the GBD 2017 round to split data into 5-year age groups. It was assumed that there was no incidence or prevalence of rheumatoid arthritis before 5 years of age.

Rheumatoid arthritis prevalence was modelled with DisMod-MR 2.1, drawing on data from 45 countries and using mean BMI as a predictive covariate to help predict estimates in countries with no primary data. Data on excess mortality were first calculated by dividing available prevalence datapoints by corresponding cause-specific mortality data by age, sex, year, and location. These derived excess mortality data were modelled in MR-BRT by age and sex with a prior on the Healthcare Access and Quality Index,^18^ such that as this index increased excess mortality decreased, and were then included in the model (appendix pp 5–6). Uncertainty was propagated by sampling 100 model runs (draws) at each computational step and combining uncertainty from multiple data sources and data adjustments. Uncertainty intervals (UIs) were defined as the 2·5th and 97·5th percentiles of the ordered draws from 100 model runs.

Data from seven countries (France, Lebanon, Spain, Finland, USA, Canada, and Sri Lanka) classifying severity according to Health Assessment Questionnaire scores were meta-analysed and scaled to fit to 1 for mild, moderate, and severe disease. This process involved normalising the disability weights derived from Health Assessment Questionnaire scores such that the maximum disability weight was equal to 1. Cutoff scores were set as less than 1 for mild, 1 to less than 2 for moderate, and 2 or greater for severe rheumatoid arthritis (appendix p 6). YLDs were calculated by multiplying the prevalence of each severity category by the severity-specific disability weights. Disability weights in GBD range from 0 (equivalent to perfect health) to 1 (signifying full loss of health; details in appendix p 6).^19^ YLDs were corrected for independent co-occurrence with any other condition for each age, sex, year, and location category.^17^ DALYs were calculated by summing YLLs and YLDs.

### Risk estimation

Smoking was the only risk factor included in GBD 2021 for rheumatoid arthritis. GBD 2021 included risk factors for which there was probable evidence of a risk–outcome relationship, included more than one study type, at least two cohorts, no substantial and unexplained heterogeneity, low risk of confounding and selection bias, and biologically acceptable dose–response gradients. Relative risk data for smoking and rheumatoid arthritis were derived from six published cohort or case-control studies. Additional information can be found in the GBD 2019 risk factor publication.^20^

### Estimate projections

Forecasted global and regional cases of rheumatoid arthritis to the year 2050 were computed by forecasting prevalence rates and population estimates.^21^ Health outcomes are closely tied to Socio-demographic Index (SDI), a compound indicator of income per capita, years of schooling, and total fertility in women younger than 25 years.^17^ After forecasting mortality,^22^ the following regression was used to forecast the ratio of mortality to prevalence: logit(*R*_y,a,s,l_) = (β_1_ + δ_a,s,l_)SDI_y,l_ + β_0_ + γ_a,s,l_ + ɛ_y,a,s,l_, In this equation *R*_y,a,s,l_ is the year-age-sex-location-specific ratio for a given cause, the covariate SDI_y,l_ is the location-year-specific SDI, γ_a,s,l_ is the age-sex-location-specific random intercept, δ_a,s,l_ is the age-sex-location-specific slope on SDI, and ɛ_y,a,s,l_ is the residual term. Prevalence was then calculated by dividing forecasted mortality by the forecasted mortality-to-prevalence ratio after transforming values back into normal space. Results were truncated by calculating the mean absolute deviation across dimensions of age, sex, and location, then finding floor and ceiling values based on a multiplier for the mean absolute deviation that covers 97·5% of the results across all dimensions. This is a more flexible truncation method than a hard cutoff, as it adapts to the bounds of the data and allows elimination of extreme values obtained in the division of mortality by the mortality-to-prevalence ratio. The prevalence rates were shifted to align with the draws in the last year of GBD data in logit space. To obtain forecasted cases, forecasted rates were multiplied by forecasted population values.^21^ Validation testing was conducted with estimates from 1990 to 2010 to project prevalence from 2010 to 2019 by age, sex, location, and year. The projections were then compared to the GBD prevalence results for this period by calculating the root mean squared error and bias (calculated as the median value of all predicted minus observed values by age, sex, location, and year). In all the four tests the model root mean squared error was less than 0·0001 and bias less than 0·0001. A Das Gupta decomposition analysis^23^ was done to determine the relative contributions of population growth, population ageing, and changes in prevalence unrelated to demographics to the change in case numbers between 2020 and 2050.

### Role of the funding source

The funder of the study had no role in the study design, data collection, data analysis, data interpretation, or writing of the report.

## Results

Non-fatal estimates were based on input of 98 prevalence and 25 incidence studies in addition to claims data, providing data from 45 countries covering all seven GBD super-regions and 16 of the 21 GBD regions. Subnational data were available from 20 countries (appendix p 5). Fatal estimates were derived from a total of 2595 sources with cause of death data, from 120 countries.

In 2020, there were an estimated 17·6 million (95% UI 15·8–20·3) people (all ages) living with rheumatoid arthritis globally, representing an increase of 121% (117–125) since 1990. The age-standardised global prevalence rate was 208·8 cases (186·8–241·1) per 100 000 population (table), representing an increase of 14·1% (12·7–15·4) since 1990 (appendix pp 8–20).

**Table:** Prevalence and burden of DALYs and deaths due to rheumatoid arthritis in 2020, and changes from 1990 to 2020, by sex and region

	**Number of prevalent cases, 2020**	**Age-standardised prevalence rate**		**Number of deaths, 2020**	**Age-standardised death rate**		**Number of DALYs**		**Age-standardised DALY rate**	
			Rate per 100 000, 2020	Percentage change, 1990–2020	Rate per 100 000, 2020	Percentage change, 1990–2020	Number, 2020	Percentage change, 1990–2020	Rate per 100 000, 2020	Percentage change, 1990–2020
**Global**	**17 600 000 (15 800 000 to 20 300 000)**	**208·8 (186·8 to 241·1)**	**14·1% (12·7 to 15·4)**	**38 300 (33 500 to 44 000)**	**0·5 (0·4 to 0·5)**	**−23·8% (−29·3 to −17·5)**	**3 060 000 (2 320 000 to 3 860 000)**	**96·2% (88·6 to 102·6)**	**36·4 (27·6 to 45·9)**	**−1·0% (−5·5 to 2·2)**
Males	4 870 000 (4 310 000 to 5 710 000)	119·8 (106·3 to 140·0)	17·2% (15·6 to 18·7)	12 400 (9200 to 14 300)	0·3 (0·3 to 0·4)	−16·5% (−30·9 to −6·3)	894 000 (679 000 to 1 130 000)	106·7% (94·6 to 116·2)	22·3 (17·0 to 28·0)	2·4% (−4·7 to 8·0)
Females	12 700 000 (11 400 000 to 14 500 000)	293·5 (262·7 to 336·3)	13·6% (12·0 to 15·0)	25 900 (22 200 to 30 400)	0·6 (0·5 to 0·7)	−25·9% (−32·3 to −18·5)	2 170 000 (1 650 000 to 2 740 000)	92·2% (84·2 to 99·5)	49·7 (37·6 to 63·0)	−1·6% (−6·5 to 2·3)
**Central Europe, eastern Europe, and central Asia**	**1 030 000 (914 000 to 1 180 000)**	**186·9 (162·8 to 215·7)**	**24·2% (21·7 to 26·7)**	**1900 (1770 to 2040)**	**0·3 (0·3 to 0·3)**	**−19·1% (−25·0 to −12·6)**	**183 000 (139 000 to 228 000)**	**30·7% (24·8 to 35·8)**	**32·4 (24·2 to 40·9)**	**8·1% (2·7 to 12·5)**
Central Asia	171 000 (149 000 to 197 000)	182·7 (160·0 to 208·0)	41·7% (36·9 to 47·6)	124 (112 to 139)	0·2 (0·2 to 0·2)	530·6% (388·9 to 714·0)	26 500 (19 100 to 34 500)	166·9% (146·6 to 187·7)	28·9 (21·1 to 37·3)	59·0% (48·4 to 71·5)
Central Europe	349 000 (313 000 to 397 000)	202·1 (177·6 to 232·9)	20·0% (17·7 to 22·7)	488 (434 to 533)	0·2 (0·2 to 0·2)	−57·8% (−62·5 to −54·7)	56 100 (41 600 to 72 800)	12·0% (4·1 to 17·8)	31·7 (22·8 to 42·1)	−8·5% (−16·6 to −2·4)
Eastern Europe	514 000 (450 000 to 588 000)	176·6 (152·3 to 204·0)	22·0% (19·1 to 25·3)	1290 (1190 to 1400)	0·4 (0·3 to 0·4)	6·4% (−2·4 to 18·2)	100 000 (78 000 to 123 000)	25·5% (21·0 to 30·5)	33·4 (25·5 to 41·3)	12·7% (8·1 to 17·1)
**High income**	**4 920 000 (4 520 000 to 5 450 000)**	**288·1 (262·6 to 324·0)**	**11·3% (9·6 to 12·8)**	**9640 (8260 to 10 400)**	**0·4 (0·3 to 0·4)**	**−43·8% (−47·4 to −40·9)**	**798 000 (602 000 to 992 000)**	**46·1% (40·3 to 49·8)**	**45·4 (33·5 to 57·6)**	**−5·7% (−10·5 to −2·6)**
Australasia	158 000 (143 000 to 178 000)	355·6 (318·1 to 406·9)	13·0% (7·2 to 18·4)	291 (251 to 322)	0·5 (0·5 to 0·6)	−45·8% (−52·0 to −40·4)	25 400 (18 900 to 32 500)	83·6% (68·4 to 93·0)	55·9 (41·1 to 72·6)	−5·7% (−13·9 to −0·7)
High-income Asia Pacific	910 000 (810 000 to 1 040 000)	266·9 (236·7 to 312·9)	−8·1% (−11·1 to −5·1)	2420 (1940 to 2710)	0·4 (0·3 to 0·5)	−53·7% (−58·4 to −50·9)	156 000 (118 000 to 196 000)	35·4% (29·6 to 39·3)	43·3 (31·4 to 56·4)	−23·8% (−29·3 to −19·6)
High-income North America	1 620 000 (1 490 000 to 1 750 000)	300·5 (278·5 to 322·3)	18·2% (16·7 to 19·6)	2500 (2180 to 2700)	0·4 (0·3 to 0·4)	−29·3% (−33·8 to −26·0)	254 000 (191 000 to 319 000)	72·5% (67·8 to 77·1)	46·3 (34·4 to 58·9)	4·3% (0·8 to 7·4)
Southern Latin America	210 000 (187 000 to 237 000)	266·1 (235·3 to 302·1)	62·0% (54·5 to 72·3)	416 (378 to 450)	0·5 (0·4 to 0·5)	−11·1% (−19·1 to −2·3)	36 300 (26 900 to 46 000)	126·7% (108·9 to 140·5)	45·5 (33·5 to 58·0)	32·9% (22·5 to 41·9)
Western Europe	2 020 000 (1 840 000 to 2 290 000)	284·9 (252·4 to 328·7)	11·0% (9·3 to 12·5)	4010 (3490 to 4350)	0·4 (0·3 to 0·4)	−49·9% (−53·6 to −46·6)	327 000 (245 000 to 410 000)	28·5% (22·0 to 33·4)	44·5 (32·4 to 57·2)	−7·1% (−12·8 to −2·9)
**Latin America and Caribbean**	**1 690 000 (1 500 000 to 1 930 000)**	**269·7 (238·8 to 306·6)**	**24·2% (21·2 to 26·5)**	**3560 (3210 to 3850)**	**0·6 (0·5 to 0·7)**	**−28·2% (−34·3 to −23·4)**	**304 000 (229 000 to 384 000)**	**153·7% (144·0 to 164·4)**	**48·7 (37·0 to 61·4)**	**8·2% (3·6 to 12·6)**
Andean Latin America	268 000 (241 000 to 301 000)	427·8 (385·9 to 479·2)	63·1% (54·8 to 70·6)	363 (307 to 417)	0·7 (0·6 to 0·8)	−30·9% (−48·3 to −9·2)	42 200 (29 800 to 54 800)	227·0% (196·7 to 251·1)	68·2 (48·5 to 87·9)	34·5% (20·9 to 45·0)
Caribbean	79 800 (70 300 to 93 800)	153·4 (134·9 to 180·5)	32·3% (27·4 to 36·7)	250 (223 to 295)	0·5 (0·4 to 0·6)	−15·1% (−27·0 to −2·0)	15 100 (11 500 to 19 400)	114·4% (97·3 to 128·2)	29·1 (22·0 to 37·3)	14·2% (5·8 to 21·9)
Central Latin America	915 000 (809 000 to 1 020 000)	357·2 (316·8 to 398·0)	23·8% (20·6 to 26·9)	2310 (2030 to 2560)	1·0 (0·9 to 1·1)	−32·5% (−39·1 to −26·4)	173 000 (134 000 to 216 000)	170·0% (158·1 to 182·7)	68·2 (53·1 to 84·8)	3·5% (−2·2 to 9·1)
Tropical Latin America	430 000 (370 000 to 505 000)	169·0 (144·8 to 197·8)	0·8% (−2·0 to 3·9)	633 (567 to 678)	0·3 (0·2 to 0·3)	−8·5% (−15·5 to −2·1)	73 300 (54 200 to 97 600)	105·6% (96·4 to 116·6)	28·9 (21·4 to 38·5)	−1·3% (−4·9 to 4·1)
**North Africa and Middle East**	**683 000 (599 000 to 797 000)**	**116·8 (104·2 to 134·6)**	**50·1% (46·3 to 54·6)**	**546 (462 to 686)**	**0·1 (0·1 to 0·2)**	**−22·4% (−38·9 to 2·8)**	**107 000 (76 700 to 144 000)**	**216·1% (190·8 to 238·0)**	**18·8 (13·6 to 24·8)**	**27·8% (18·3 to 36·9)**
**South Asia**	**3 250 000 (2 850 000 to 3 820 000)**	**204·8 (181·7 to 238·1)**	**41·3% (37·8 to 44·5)**	**9930 (7490 to 14 300)**	**0·8 (0·6 to 1·2)**	**−21·6% (−34·9 to −7·5)**	**614 000 (466 000 to 766 000)**	**180·7% (161·9 to 199·7)**	**40·6 (31·2 to 49·6)**	**10·2% (0·4 to 20·1)**
**Southeast Asia, east Asia, and Oceania**	**5 360 000 (4 750 000 to 6 280 000)**	**195·2 (171·4 to 228·9)**	**15·4% (12·5 to 18·0)**	**12 300 (10 200 to 14 800)**	**0·5 (0·4 to 0·6)**	**−8·7% (−30·3 to 13·6)**	**959 000 (730 000 to 1 230 000)**	**109·3% (91·9 to 127·4)**	**35·2 (26·7 to 44·7)**	**1·1% (−8·3 to 9·1)**
East Asia	4 830 000 (4 290 000 to 5 630 000)	238·4 (208·8 to 279·4)	16·6% (13·2 to 19·5)	11 300 (9320 to 13 600)	0·6 (0·5 to 0·7)	−11·7% (−33·9 to 11·3)	866 000 (660 000 to 1 110 000)	105·4% (86·9 to 123·5)	42·6 (32·4 to 54·4)	1·5% (−9·3 to 9·8)
Oceania	5460 (4470 to 6710)	50·5 (42·1 to 60·7)	18·0% (13·8 to 22·4)	0·101 (0·0477 to 0·248)	0·0 (0·0 to 0·0)	−12·2% (−39·6 to 35·5)	774 (511 to 1 120)	175·7% (155·8 to 204·5)	7·1 (4·8 to 10·1)	16·2% (7·4 to 27·6)
Southeast Asia	525 000 (446 000 to 638 000)	74·9 (64·2 to 90·2)	34·8% (31·1 to 39·0)	1030 (746 to 1190)	0·2 (0·1 to 0·2)	−5·2% (−23·2 to 10·3)	92 700 (66 200 to 118 000)	154·1% (135·4 to 173·4)	13·8 (10·1 to 17·3)	17·1% (7·2 to 24·7)
**Sub-Saharan Africa**	**641 000 (538 000 to 765 000)**	**96·3 (83·8 to 113·2)**	**3·8% (2·0 to 5·4)**	**446 (378 to 930)**	**0·1 (0·1 to 0·2)**	**−31·6% (−45·4 to −7·5)**	**98 700 (68 100 to 131 000)**	**120·4% (109·3 to 130·7)**	**15·2 (10·9 to 19·5)**	**−5·8% (−10·8 to −0·5)**
Central Sub-Saharan Africa	78 900 (66 300 to 93 100)	103·5 (89·9 to 118·3)	19·4% (15·3 to 23·4)	19·6 (5·64 to 205)	0·0 (0·0 to 0·5)	−31·4% (−61·3 to −17·6)	11 100 (7 420 to 16 300)	189·7% (159·2 to 221·6)	14·6 (10·0 to 23·2)	13·5% (0·6 to 26·8)
Eastern Sub-Saharan Africa	197 000 (164 000 to 236 000)	85·5 (74·6 to 98·9)	11·7% (9·6 to 13·3)	35·5 (12·6 to 381)	0·0 (0·0 to 0·3)	−38·0% (−58·7 to −26·3)	28 000 (17 800 to 39 800)	156·8% (133·3 to 169·3)	12·0 (8·0 to 17·0)	7·6% (−0·6 to 12·4)
Southern Sub-Saharan Africa	188 000 (163 000 to 215 000)	260·5 (227·9 to 296·1)	−10·0% (−12·2 to −8·2)	379 (327 to 429)	0·7 (0·6 to 0·8)	−23·1% (−38·0 to 7·2)	34 800 (26 500 to 43 500)	58·5% (47·4 to 72·8)	50·5 (39·0 to 61·9)	−18·8% (−24·5 to −9·9)
Western Sub-Saharan Africa	177 000 (144 000 to 220 000)	61·3 (51·6 to 75·1)	25·9% (22·5 to 29·4)	12·6 (6·24 to 21·1)	0·0 (0·0 to 0·0)	19·4% (−21·1 to 101·6)	24 700 (16 000 to 35 100)	207·1% (196·3 to 222·4)	8·5 (5·7 to 11·9)	24·3% (19·9 to 29·8)

Values in parentheses are 95% uncertainty intervals. Super-region and region numbers do not sum to the global prevalence due to rounding and modelling adjustments for nations with populations below 50 000. DALY=disability-adjusted life-year.

**Figure 1:**
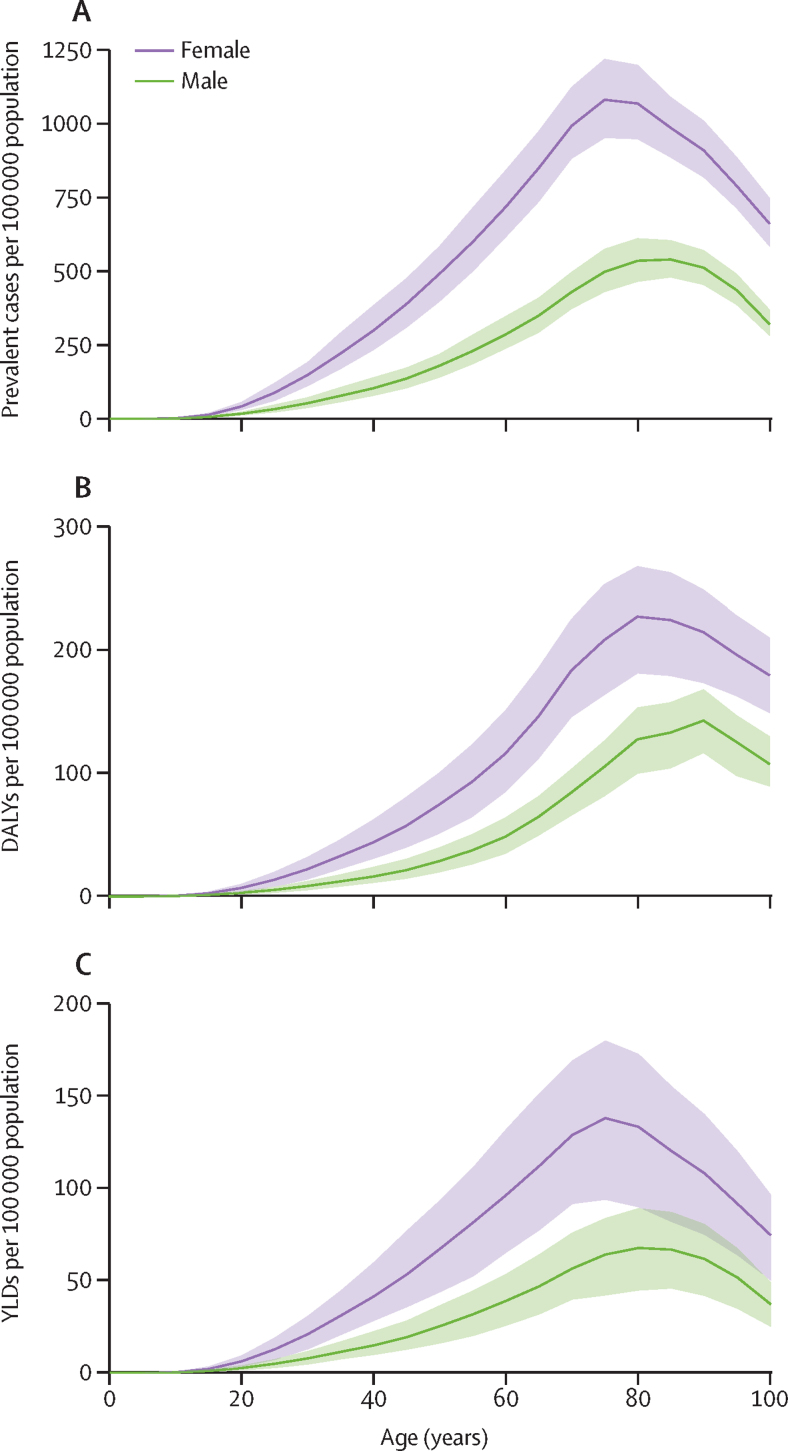
Global prevalence, DALY, and YLD rates of rheumatoid arthritis in 2020 by sex and age (A) Prevalent cases per 100 000 population. (B) DALYs per 100 000 population. (C) YLDs per 100 000 population. Shaded areas represent 95% uncertainty intervals. DALY=disability-adjusted life-year. YLD=years lived with disability.

Across all estimation years, prevalence of rheumatoid arthritis was more common in females than in males, with a 2020 global age-standardised prevalence rate of 293·5 (95% UI 262·7–336·3) per 100 000 population for females and 119·8 (106·3–140·0) per 100 000 for males (table). The age-standardised female-to-male prevalence ratio was 2·45 [2·40–2·47]). The age-specific prevalence rate of rheumatoid arthritis peaked in the 75–79 years age group in 2020, with 828·2 cases (730·3–934·0) per 100 000 population (figure 1).

**Figure 2:**
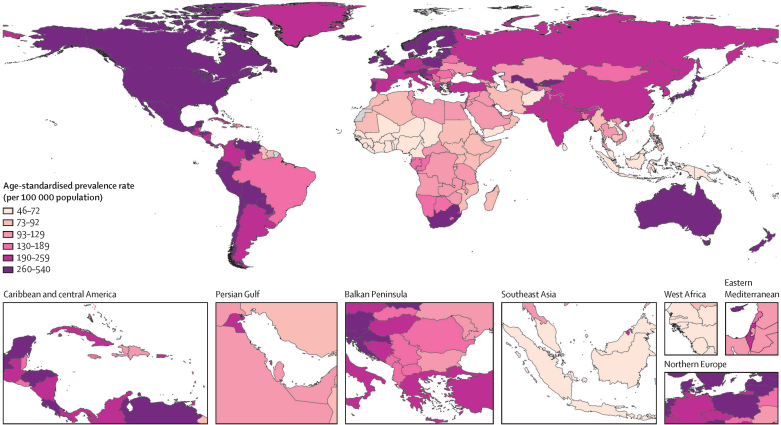
Age-standardised prevalence rate of rheumatoid arthritis for male and female sexes combined in 2020

Among GBD super-regions, the age-standardised prevalence rate of rheumatoid arthritis was highest in the high-income super-region (288·1 [262·6–324·0] per 100 000 population) and in Latin America and the Caribbean (269·7 [238·8–306·6] per 100 000), and lowest in sub-Saharan Africa (96·3 [83·8–113·2] per 100 000) and North Africa and the Middle East (116·8 [104·2–134·6] per 100 000). Regional variations ranged from 50·5 (42·1–60·7) per 100 000 in Oceania to 427·8 (385·9–479·2) per 100 000 in Andean Latin America (table, figure 2). Prevalence at the region and country levels is provided in the appendix (pp 8–20).

In 2020, there were an estimated 38 300 deaths (95% UI 33 500 to 44 000) due to rheumatoid arthritis globally, with an age-standardised death rate of 0·5 (0·4 to 0·5) per 100 000. Between 1990 and 2020, there was a decrease in global age-standardised death rates due to rheumatoid arthritis (–23·8% [–29·3 to –17·5]), with the largest decline in the high-income super-region (–43·8% [–47·4 to –40·9]) and the smallest in the southeast Asia, east Asia, and Oceania super-region (–8·7 [–30·3 to 13·6]; table). An increase in global age-standardised death rate was seen in three regions: central Asia (530·6% [388·9 to 714·0]), eastern Europe (6·4% [–2·4 to 18·2]), and western sub-Saharan Africa (19·4% [–21·1 to 101·6]).

Globally, in 2020, there were 719 000 YLLs (95% UI 644 000–828 000) due to rheumatoid arthritis for male and female sexes combined, with an age-standardised YLL rate of 8·6 (7·7–9·9) per 100 000 population. The age-standardised YLL rate for females (10·8 [9·5–12·8] per 100 000) was almost double that for males (6·2 [4·6–7·2] per 100 000).

Rheumatoid arthritis resulted in 3·06 million (95% UI 2·32 to 3·86) global DALYs (all ages) in 2020, accounting for 0·1% (0·1 to 0·1) of total global DALYs. The global age-standardised DALY rate for rheumatoid arthritis in 2020 was 36·4 (27·6 to 45·9) per 100 000 population (table; appendix p 8). There was no change in the global age-standardised DALY rate between 1990 and 2020 (–1·0% [–5·5 to 2·2]), although regional differences were observed; for example, North Africa and the Middle East had a 27·8% increase (18·3 to 36·9) from 1990 to 2020. Age-standardised DALY rates and prevalence by region are shown in the table and by country in the appendix (pp 8–20).

In 2020, 76·4% (95% UI 68·3–81·0) of rheumatoid arthritis DALYs were due to YLDs and the remainder were due to YLLs. Total YLD count for rheumatoid arthritis was 2 340 000 (1 590 000–3 130 000). Between 1990 and 2020, the global age-standardised YLD rate for rheumatoid arthritis increased by 13·8% (12·3–15·6), from 24·4 YLDs (16·5–32·8) to 27·8 YLDs (18·9–37·1) per 100 000 population (appendix p 8).

As with prevalence, YLD and DALY rates for females were considerably higher than for males across all age groups. YLDs for females peaked in the 70–74 years age group and DALYs around age 75–79 years, followed by a decrease. For males, the peak YLD rate was in the 75–79 years age group, and DALYs peaked around age 85–89 years (figure 1).

Smoking was the only risk factor for rheumatoid arthritis included in GBD 2021, accounting for 217 000 (103 000–320 000) or 7·1% (95% UI 3·6–10·3) of DALYs due to rheumatoid arthritis in 2020. Rheumatoid arthritis DALY rates attributable to smoking were considerably higher for males (3·5 [1·7–5·1] per 100 000 population) than for females (2·1 [1·0–3·2] per 100 000).

**Figure 3:**
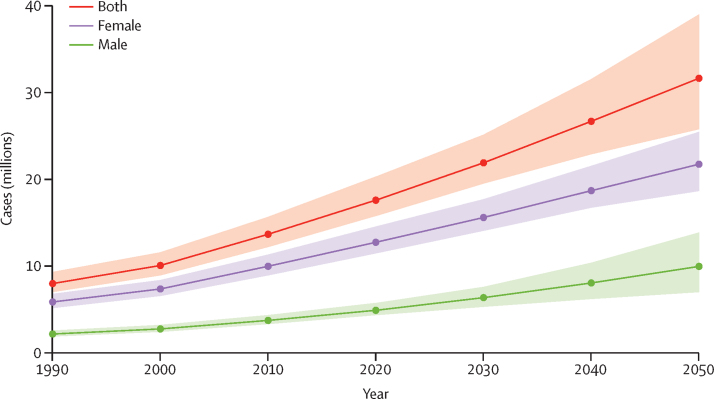
Total global cases of rheumatoid arthritis forecasted to the year 2050 Shaded areas represent 95% uncertainty intervals.

**Figure 4:**
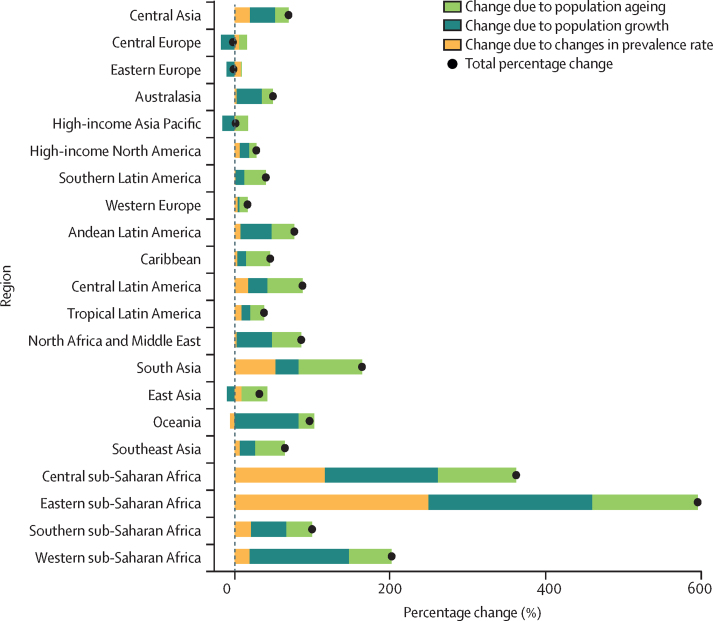
Decomposition of projected change in the number of prevalent rheumatoid arthritis cases by region, 2020–50

Based on forecasted changes in population, we estimate 31·7 million (95% UI 25·8–39·0) individuals worldwide will have rheumatoid arthritis in 2050 (figure 3), constituting an 80·2% (63·3–92·1) increase in the number of cases from 2020 to 2050. Of the total rheumatoid arthritis cases in 2050, we estimate that 68·7% (65·2–72·3) will be female (21·7 million [18·6–25·5]). The regions with no or little forecasted change in cases from 2020 to 2050 are central Europe, eastern Europe, and high-income Asia Pacific. The regions with a projected increase of over 200% are central, eastern, and western sub-Saharan Africa (figure 4; for age-standardised prevalence and for cases in 2050 see appendix p 22).

A decomposition analysis globally and by region shows the relative contribution of population growth, population ageing, and changes in prevalence rate to the forecasted increase in cases (figure 4). Population growth was the largest contributor in most locations. Regions with little change in forecasted case numbers between 2020 and 2050 also had negative population growth, including central and eastern Europe and the high-income Asia Pacific region. East Asia also showed negative population growth; however, population ageing contributed to the increase in forecasted prevalence of rheumatoid arthritis in that region. Changes in prevalence rate were the largest contributor to the increase in case numbers in eastern sub-Saharan Africa.

## Discussion

This study provides updated global estimates of mortality, prevalence, and disability (YLDs and DALYs) for rheumatoid arthritis, as well as providing forecasted estimates of rheumatoid arthritis disease burden to the year 2050 for the first time. Global age-standardised death rate decreased by around 23·8% between 1990 and 2020, and decreased in all super-regions, most prominently in the high-income super-region, where there was a decrease in deaths due to rheumatoid arthritis of approximately 43·8% over the same period. The decrease in mortality in high-income countries might reflect better disease control and outcomes following early intervention and treat-to-target treatment strategies in high-income but not lower-income countries over this time.^24^, ^25^

The GBD age-standardised global prevalence rate of rheumatoid arthritis was 208·8 cases per 100 000 (0·21%) in 2020. This rate is considerably lower than that found in a 2021 systematic review and meta-analysis by Almutairi and colleagues^26^ of papers published between January, 1980, and June, 2019, which reported the global pooled period-prevalence of rheumatoid arthritis to be 460 per 100 000 (0·46%). The reasons for this discrepancy are likely to be multifactorial, including different data sources due to different search strategies and different inclusion and exclusion criteria (123 sources in GBD *vs* 67 studies included in Almutairi and colleagues’ review), different case definitions, different meta-analysis models and different definitions of prevalence (ie, age-standardised prevalence reported in GBD). Another systematic review by the same group included 60 studies and also found a greater mean period prevalence of 51 cases per 100 000 (0·51%) between 1955 and 2015.^27^ Almutairi and colleagues hypothesised that the GBD use of modelling to account for data sparsity across under-represented regions under-estimates true prevalence. The accuracy of any future global prevalence estimates for rheumatoid arthritis will no doubt be improved by more data from currently under-represented regions. Our estimates show a lower prevalence in low-income and middle-income countries than high-income countries, a gradient that has been noted before for rheumatoid arthritis^28^ but is also seen in other autoimmune diseases such as multiple sclerosis and type 1 diabetes. Ignoring such a gradient in a meta-analysis dominated by studies from high-income countries would lead to an overestimate*.*

The non-fatal estimates generated in this study show that the number of cases of rheumatoid arthritis globally more than doubled from 1990 to 2020. This increase is in line with the increasing prevalence of other autoimmune diseases globally,^29^ and is likely to be multifactorial due to population ageing or unknown or unmeasured risk factors, but could also be confounded by methodological issues such as data sparsity. Also in line with other autoimmune diseases was the higher prevalence observed in females than in males across all age groups.^30^ Despite this observed increase in prevalence, cases of rheumatoid arthritis might be under-reported. Not all patients have access to health-care services, especially in low-income countries, where confirmation of a diagnosis of rheumatoid arthritis by a physician might not be available. Additionally, under-resourced populations from high-income countries might also be under-reported, as many migrant workers in these countries might not have access to health insurance or health-care services.

By 2050, we forecast that there will be 31·7 million individuals worldwide with rheumatoid arthritis, 68·7% of whom will be female. In most regions, the increase is mainly due to population growth and ageing, but in sub-Saharan Africa and south Asia, increases in prevalence rate, probably driven by increasing economic development of countries, also contribute to the forecasted increase. These forecasts are based on the reference definition in this study, the 1987 ACR criteria, which identify established rheumatoid arthritis, and thus only those with later disease are included. Data sources using diagnostic criteria other than the reference criteria, such as the 2010 ACR–EULAR criteria,^16^ were adjusted in the modelling process. As further studies use updated definitions of rheumatoid arthritis (which emphasise rheumatoid arthritis characteristics that emerge early in the course of the disease)^31^ and data become available for inclusion in the model, the prevalence of rheumatoid arthritis is likely to increase as those with early rheumatoid arthritis (who would not meet the criteria of established rheumatoid arthritis) would be included. This change in definition has not been included in the forecasted rates reported here. As population-based rheumatoid arthritis data using the 2010 ACR–EULAR criteria emerge, the reference case and data adjustments can be modified accordingly in future GBD iterations.

A strength of this study is that it provides a unique global perspective of disease burden that GBD provides over time. GBD has rigorous and standardised methodologies for obtaining fatal and non-fatal data and generating estimates. These methodologies are reviewed and, where necessary, updated at each GBD iteration. GBD methods aim to correct for changes in case definitions or other advancements in diagnosis over time through crosswalking. In addition, for the first time, the current GBD study included forecasted prevalence predictions to 2050.

This analysis reported on smoking as a risk factor for rheumatoid arthritis and strategies to reduce smoking globally should reduce rheumatoid arthritis prevalence, along with benefiting many other health conditions. We estimated the proportion of rheumatoid arthritis attributable to smoking in our risk factor analyses. These estimates changed by age, sex, and location according to the changes in risk exposure: a decrease in most parts of the world since 1990, with the exception of eastern Europe and central Asia, where the attributable DALYs continue to show some increase. There is a growing body of evidence addressing risk factors for rheumatoid arthritis and consensus building for both primary and secondary prevention.^32^, ^33^, ^34^, ^35^, ^36^ Based on currently available evidence for potentially modifiable risks for rheumatoid arthritis, a narrative review^32^ recommended cessation of smoking, reducing occupational exposure to silica and dusts, maintaining a healthy weight, maintaining good dental hygiene, maximising breast feeding, maximising dietary quality, avoidance of high-salt diets, increasing intake of omega-3 fatty acids and fish, reducing consumption of sugar-sweetened soft drinks, consuming moderate levels of alcohol, and remaining vitamin D replete. New evidence suggests that up to 40% of cases of rheumatoid arthritis are potentially modifiable through a range of lifestyle factors, including maintaining a healthy weight and healthy diet, maintaining good dental hygiene, and reducing exposure to occupational risks.^32^ These factors could vary by region. Only smoking has been included as a risk in GBD to date, and an opportunity exists to build on this in subsequent analyses. Screening first-degree relatives of people with early rheumatoid arthritis for their risk profile and implementing targeted lifestyle prevention strategies have potential to reduce the forecasted 2050 prevalence.

Although GBD provides estimates of global disease burden, data sparsity, particularly from low-income and middle-income regions, remains a significant limitation for rheumatoid arthritis. Non-fatal estimates are largely based on modelling, with actual non-fatal data from population-based studies or insurance claims records (from high-income countries only) available from 45 of 204 countries. Therefore, differences between regions should be interpreted with caution. For example, although prevalence was lowest in western sub-Saharan Africa, Oceania, and southeast Asia, these regions had limited sources for data inclusion. The inclusion of claims data from the USA and Taiwan, both high-income countries, might not represent the patients seen in all regions, although the GBD modelling methods apply adjustments to account for these alternate case definitions. In addition, the heterogeneity of the studies contributing data leads to greater uncertainty and requires methodological adjustments, including standardisation of case definitions. The uncertainty interval is tighter in locations with good data coverage, such as high-income countries, whereas in data-sparse locations (eg, countries in sub-Saharan Africa), the uncertainty interval tends to be wider. Currently, extra-articular manifestations of rheumatoid arthritis are not taken into account within the estimates, and such manifestations can occur in more than a third of people with rheumatoid arthritis, with inflammation in other parts of the body, including the eyes, lungs, heart, and other internal organs.^37^ Extra-articular involvement is associated with poorer outcomes and increased mortality in rheumatoid arthritis,^37^ although the incidence of severe extra-articular manifestations has declined over time with early, effective therapy.^38^

Outcomes in rheumatoid arthritis, including prevention of disability, are dependent on timely access to treatment, including conservative care, and adequate control of disease activity to maintain improved ability to undertake activities of daily living. Early intervention within the so-called window of opportunity and use of treat-to-target strategies have been shown to substantially reduce disease severity and subsequent disability and mortality in rheumatoid arthritis.^24^, ^25^ Therefore, global variation in access to treatment is expected to impact rheumatoid arthritis severity across regions; however, there are currently few studies reporting data on rheumatoid arthritis severity globally. Going forward, more epidemiological studies addressing risk factors, incidence, prevalence, severity, and mortality are required, particularly in low-income and middle-income regions, to better inform and improve the accuracy of GBD estimates. Work to identify barriers and enablers to epidemiological studies in regions of data-sparsity is required, in addition to better resources to support such studies. Severity distribution estimates are likely to vary considerably and the wholesale application of the estimates to all regions is a current limitation likely to lead to an underestimation of rheumatoid arthritis disease burden, particularly in countries with poor access to care. In future work, we plan to approximate a gradient in rheumatoid arthritis severity that is linked to access to treatment by analysing effect sizes of the main treatment options from Cochrane review libraries, and linking this to information on access to various treatments in locations with data on rheumatoid arthritis severity.

GBD cause of death data for rheumatoid arthritis are derived from civil registration and vital statistics registries and might under-represent rheumatoid arthritis as a cause of death, and regional variations might occur. A 2015 study looking at death certificates among patients with rheumatoid arthritis in the USA found that only 17·7% of deaths had rheumatoid arthritis mentioned on the death certificate.^39^ A similar study looking at a Finnish rheumatoid arthritis population from the 1980s found that the proportions were 53–65%.^40^ Thus, some deaths not assigned to rheumatoid arthritis as the underlying cause might be directly caused by rheumatoid arthritis. While GBD estimates mortality directly attributable to rheumatoid arthritis, it does not capture mortality that might be increased in association with rheumatoid arthritis and related treatments, such as cardiovascular disease, infection, malignancy, and respiratory disease. Fatal estimates for rheumatoid arthritis are also limited by a lack of strong predictive covariates. Therefore, the accuracy of estimates is limited in data-sparse locations, particularly sub-Saharan Africa and locations that only report verbal autopsy data, because rheumatoid arthritis cannot be captured as an underlying cause in verbal autopsy interviews.

Data forecasting has shown a substantial increase in rheumatoid arthritis prevalence is expected by 2050, highlighting the need for global projects to control the burden of rheumatoid arthritis, particularly in low-income and middle-income countries. These projections have not accounted for the effect of COVID-19 on the burden of rheumatoid arthritis, which could include reduced access to pharmaceutical and non-pharmaceutical treatment regimens and higher mortality in older adults. Addressing the unmet rehabilitation need, such as the WHO Rehabilitation 2030 call for action,^41^ is imperative if disability is to be minimised, and equitable rehabilitation services for people with rheumatoid arthritis are required globally. Adequate resourcing of programmes that target both prevention and modification of risk factors such as smoking^42^ and obesity will also be important globally. We found a higher prevalence of rheumatoid arthritis among females than males; however, case fatality was higher in males. The higher prevalence among females is in keeping with other autoimmune disorders and is hypothesised to be related to differences in the sex chromosomes and hormonal factors,^43^ and evidence suggests that higher case-fatality in males might be due to innate and adaptive immune responses in addition to environmental, dietary, and lifestyle factors.^44^

While use of biological or targeted synthetic DMARDs is now widespread in high-income regions, drugs such as methotrexate and sulfasalazine, used as monotherapy or in combinations, remain very effective and affordable conventional synthetic DMARD treatment options for patients with rheumatoid arthritis globally, and 2·5 mg methotrexate tablets are listed for inflammatory arthritis on the WHO Model List of Essential Medicines.^45^ Access to an effective dose of methotrexate (20 mg per week) early in the disease course of rheumatoid arthritis can significantly reduce disease burden; however in low-income and middle-income regions, access to medical professionals who can diagnose rheumatoid arthritis and prescribe and monitor methotrexate at an appropriate dose can be limited. A qualitative study of physicians from 29 African countries assessed barriers to methotrexate prescribing in low versus medium–high Human Development Index countries, and found methotrexate dosing to be similar. Barriers to methotrexate use included inconsistent supply, financial restrictions (such as cost of travelling to dispensing sites), patient hesitancy related to cultural beliefs and societal roles, few prescribers, prevalent infections (especially viral hepatitis, tuberculosis, and HIV), and availability and cost of safety monitoring.^46^ A clearer picture of how these potentially modifiable factors might influence outcomes could be gained if treatment data were collected by GBD in the future.

Rheumatoid arthritis prevalence is expected to increase to the year 2050, leading to greater burden on health systems. Although modelling suggests that this increase is primarily related to ageing and the growing population, addressing risk factors such as smoking cessation could reduce rheumatoid arthritis incidence and prevalence in some regions. Limitations in these estimates for rheumatoid arthritis include data sparsity, particularly from low-income and middle-income countries, for both fatal and non-fatal outcomes; the paucity of risk factor attribution, which was limited to smoking only, although a range of other modifiable risk factors are being increasingly recognised; and the application of a standard high-income region-derived severity distribution to all countries. GBD estimates have traditionally not addressed access to health care and treatments; however, rheumatoid arthritis is a key example of a condition in which timely access to medical care for early diagnosis, treatment, and monitoring has a crucial role in health outcomes, and access varies substantially by region. Future GBD estimates for rheumatoid arthritis should aim to address regional variations in all these factors. Although some proven interventions such as biological or targeted synthetic DMARDs are costly, making them inaccessible to low-income and middle-income countries, increasing uptake of low-cost conventional synthetic DMARDs has the potential to considerably reduce the burden of rheumatoid arthritis in all regions. Increasing global awareness of the importance of early diagnosis and treatment of rheumatoid arthritis will help to reduce the future impact of the disease.

## Data sharing

The findings of this study are supported by data available in public online repositories, data publicly available upon request of the data provider, and data not publicly available due to restrictions by the data provider. Non-publicly available data were used under license for the current study, but can be made available by the authors upon reasonable request and with permission of the data provider. Data sources used in this analysis are listed in the appendix (pp 24–28).

## Declaration of interests

B Antony reports grants or contracts from Rebecca Cooper Foundation, and a Nat Rem grant for an investigator-initiated trial biomarkers assessment support; payment or honoraria for lectures, presentations, speakers bureaus, manuscript writing or educational events from Nat Rem through a speaker fee and IRACON through travel support; all outside the submitted work. A M Briggs reports grants or contracts from Bone and Joint Decade Foundation, AO Alliance, Canadian Memorial Chiropractic College, Australian Rheumatology Association, Pan-American League of Associations for Rheumatology, World Federation of Chiropractic, and Asia Pacific League of Associations for Rheumatology, all as payments to their institution; consulting fees from WHO as personal payments; payment or honoraria for lectures, presentations, speakers bureaus, manuscript writing or educational events from the American College of Rheumatology (ACR) as personal payments; all outside the submitted work. R Buchbinder reports grants from the Australian National Health and Medical Research Council (NHMRC), HCF Foundation, Cabrini Foundation, Arthritis Australia, and Federal Department of Health to their institutions; royalties or licenses from UpToDate for plantar fasciitis authorship; all outside the submitted work. I Fillip and A Radfar report payment or honoraria for lectures, presentations, speakers’ bureaus, manuscript writing or educational events from Avicenna Medical and Clinical Research Institute, outside the submitted work. C L Hill reports grants or contracts from NHMRC, Vifor Pharmaceuticals, and Arthritis Australia; leadership or fiduciary role in other board, society, committee or advocacy group, paid or unpaid, with the Australian Rheumatology Association, Australian & New Zealand Vasculitis Society, Arthritis SA, and APLAR; all outside the submitted work. N Ismail reports leadership or fiduciary role in other board, society, committee, or advocacy group, unpaid, as Council Member and Bursar of the Malaysian Academy of Pharmacy outside the submitted work. K Krishan reports non-financial support from UGC Centre of Advanced Study, CAS II, Department of Anthropology, Panjab University, Chandigarh, India, outside the submitted work. A-F A Mentis reports grants or contracts from MilkSafe: a novel pipeline to enrich formula milk using omics technologies, a research co-financed by the European Regional Development Fund of the EU and Greek national funds through the Operational Program Competitiveness, Entrepreneurship and Innovation, under the call RESEARCH–CREATE–INNOVATE (project code T2EDK-02222), as well as from ELIDEK (Hellenic Foundation for Research and Innovation, MIMS-860); payment as an external peer reviewer for Fondazione Cariplo, Italy; serves as an editorial board member for the journals *Systematic Reviews* and *Annals of Epidemiology*, and is an associate editor for *Translational Psychiatry*; stocks in a family winery; other financial or non-financial support from the BGI group as a scientific officer; all outside the submitted work. V Shivarov reports stock or stock options with ICON; other financial or non-financial interests in ICON through salary payments; all outside the submitted work. J A Singh reports consulting fees from Crealta/Horizon, MediSys, Fidia Farmaceutici, PK Med, Two Labs, Adept Field Solutions, Clinical Care Options, Clearview Healthcare Partners, Putnam Associates, Focus Forward, Navigant Consulting, Spherix Global Insights, Mediq, Jupiter Life Science, UBM, Trio Health, Medscape, WebMD, and Practice Point Communications, the National Institutes of Health, and the ACR; payment or honoraria for speakers bureaus from Simply Speaking; support for attending meetings or travel from the steering committee of OMERACT; participation on a data safety monitoring board or advisory board with the US Food and Drug Administration Arthritis Advisory Committee; membership of the steering committee of OMERACT, a role as Chair (unpaid) of the Veterans Affairs Rheumatology Field Advisory Committee, and roles as Editor and Director (unpaid) with the UAB Cochrane Musculoskeletal Group Satellite Center on Network Metaanalysis; stock or stock options in TPT Global Tech, Vaxart Pharmaceuticals, Aytu BioPharma, Adaptimmune Therapeutics, GeoVax Labs, Pieris Pharmaceuticals, Enzolytics, Seres Therapeutics, Tonix Pharmaceuticals, and Charlotte's Web Holdings, and previous stock options in Amarin, Viking Therapeutics, and Moderna Pharmaceuticals; all outside the submitted work. H Slater reports grants from Australian Government Department of Health, Medical Research Future Fund, Western Australian Government Department of Health, Bone and Joint Decade Foundation (Sweden), Curtin University (Australia), Institute for Bone and Joint Research (Australia), Canadian Memorial Chiropractic College (Canada), all through their institution; support for attending meetings or travel from the Australian Pain Society; all outside the submitted work. All other authors declare no competing interests.
