# Supplementary material for: Global, regional, and national burden of rheumatoid arthritis, 1990–2020, and projections to 2050: a systematic analysis of the Global Burden of Disease Study 2021
Source: Lancet Rheumatol. 2023 Sep 25;5(10):e594–610. doi: 10.1016/S2665-9913(23)00211-4 (PMC10546867; doi:10.1016/S2665-9913(23)00211-4)
Supplement: Supplementary appendix [file mmc1.pdf]

THE LANCET
Rheumatology

Supplementary appendix

This appendix formed part of the original submission and has been peer reviewed. We post it as supplied by the authors.

Supplement to: GBD 2021 Rheumatoid Arthritis Collaborators. Global, regional, and national burden of rheumatoid arthritis, 1990–2020, and projections to 2050: a systematic analysis of the Global Burden of Disease Study 2021. *Lancet Rheumatol* 2023; **5:** e594–610.

The global burden of rheumatoid arthritis: estimates from the Global Burden of Disease 2021 Study

CONTENTS

	Page
**Section 1: Non-fatal data seeking**	**1**
Supplemental Figure 1. Identification of new studies via databases and registers	2
Supplemental Table 1: MR-BRT crosswalk adjustment factors for rheumatoid arthritis	3
Supplemental Table 2: Covariates	3
Supplemental Figure 2: Distribution of data sources for rheumatoid arthritis	4
Supplemental Table 3: National and subnational sources in countries with subnational estimation	5
**Section 2. Excess mortality rate (EMR) modelling**	**5**
Supplemental Table 4: Covariates used in rheumatoid arthritis mortality modelling	6
**Section 3. Severity distribution meta-analysis**	**6**
Supplemental Table 5: Severity distribution	6
Supplemental Figure 3a. Severity distribution meta-analysis of mild rheumatoid arthritis	7
Supplemental Figure 3b. Severity distribution meta-analysis of moderate rheumatoid arthritis	7
Supplemental Figure 3c. Severity distribution meta-analysis of severe rheumatoid arthritis	7
**Section 4. Results**	**8**
Supplemental Table 6: Rheumatoid arthritis prevalence and age-standardised DALY rate by region and country	8
Supplemental Figure 4: Age-standardised disability-adjusted life-years (DALYs) for rheumatoid arthritis per 100,000 population by location, 2020	21
Supplemental Table 7: Forecast of rheumatoid arthritis age-standardised prevalence and total cases globally and by region, both sexes, from 2030 to 2050	22
Data input sources included in GBD 2021 for rheumatoid arthritis modelling	24
Authors' contributions	29

This appendix provides more detailed methodology and supplemental figures, tables, and results for "**The global burden of rheumatoid arthritis: estimates from the Global Burden of Disease 2021 Study**". Portions of this appendix have been adapted from Vos et al. References are provided for adapted sections.

Vos T, Lim S, Abbafati C, et al. Global burden of 369 diseases and injuries in 204 countries and territories, 1990–2019: a systematic analysis for the Global Burden of Disease Study 2019. *The Lancet* 2020; **396**(10258): 1204-22.

Section 1. Non-fatal data seeking

For GBD 2010, a systematic review of the prevalence of RA throughout the world was conducted. Ovid MEDLINE, EMBASE, CINAHL, CAB abstracts, WHOLIS, and SIGLE databases were searched using the following search terms: (rheumatoid arthritis OR rheumatic disease* OR rheumatism) AND (prevalen* OR inciden* OR cross-sectional OR cross sectional OR epidemiol* OR survey OR population-based OR population based OR population study OR population sample OR cohort OR follow-up OR follow up OR longitudinal OR regist* OR data collection). Opportunistically encountered scientific literature and population surveys were added for GBD 2015 and GBD 2016.

A further search was conducted for GBD 2017 using the following search terms: ("Arthritis, Rheumatoid"[Mesh] AND ("Prevalence"[Mesh] OR "Incidence"[Mesh])) NOT (Meta-Analysis[ptyp] OR Letter[ptyp] OR Editorial[ptyp] OR Case Reports[ptyp] OR Review[ptyp] OR Controlled Clinical Trial[ptyp]) AND ("2013/01/01"[PDAT] : "2018/1/10"[PDAT].

For GBD 2021, we conducted a further systematic review of rheumatoid arthritis prevalence using the following search string:

("Arthritis, Rheumatoid"[Mesh] OR arthrit*) AND (prevalen*[Mesh] OR inciden*[Mesh]) AND ("2017/12/18"[PDAT] : "2019/10/18"[PDAT])

The exclusion criteria were:

1. Studies clearly not representative of the national population
2. Studies that were not population-based, eg, hospital or clinic-based studies
3. Studies that did not provide primary data on epidemiological parameters, eg, a commentary piece
4. Studies of a specific type of RA, eg, seropositive RA
5. Studies with a sample size of less than 150
6. Reviews

Opportunistically, six additional studies encountered during data review were added for GBD 2021:

1. Cook MJ, Bellou E, Bowes J, Sergeant JC, O'Neill TW, Barton A, Verstappen SMM. The prevalence of comorbidities and their impact on physical activity in people with inflammatory rheumatic diseases compared with the general population: results from the UK Biobank. *Rheumatology (Oxford)*. 2018; 57(12): 2172-2182.
2. Jeong HS, Hong SJ, Choi SJ, Kim JH, Song GG, Jung JH. Effects of oral contraceptives on rheumatoid arthritis in Korean menopausal women: A nationwide cross-sectional study. *Maturitas*. 2018; 112: 24-28.
3. Klinaki E, Katsoulis M, La Vecchia C, Trichopoulou A. Rheumatoid arthritis and cancer risk: results from the Greek European prospective investigation into cancer and nutrition cohort. *Eur J Cancer Prev*. 2018; 27(5): 502506.
4. Kumar P, Alok R, Das SK, Srivastava R, Agarwal GG. Distribution of rheumatological diseases in rural and urban areas: An adapted COPCORD Stage I Phase III survey of Lucknow district in north India. *Int J Rheum Dis*. 2018; 21(11): 1894-1899.
5. Li X, Sjöstedt C, Sundquist J, Zöller B, Sundquist K. Familial association of attention-deficit hyperactivity disorder with autoimmune diseases in the population of Sweden. *Psychiatr Genet*. 2019; 29(2): 37-43.
6. Vega-Hinojosa O, Cardiel MH, Ochoa-Miranda P. Prevalence of musculoskeletal manifestations and related disabilities in a Peruvian urban population living at high altitude. COPCORD Study. Stage I. *Reumatol Clin*. 2018; 14(5): 278-284.

In addition, data from USA claims data for 2000, 2010–2012, and 2014–2016 by state and Taiwan claims for 2016 were included. We decided not to use hospital inpatient data as we considered they would not be representative of true prevalence and that variation between countries in the proportion of true prevalent cases captured in hospital inpatient data systems would likely vary more than can be captured by a single crosswalk. We compared the rates of RA in the outpatient data from Norway, Sweden, Canada, and the USA and found implausibly large differences with the rates from the claims data. The USA outpatient rates were half the value of the claims data, and those for the other countries much lower still. For those reasons we decided not to use outpatient data. The reference ICD-10 codes for rheumatoid arthritis are M05, M06, and M08. The ICD-9 codes for rheumatoid arthritis are 714.0–714.9.

For fatal data, datapoints that were implausibly high or low globally or regionally, conflicted with established age or temporal patterns, conflicted with other data sources from similar locations, or came from verbal autopsy sources were excluded.

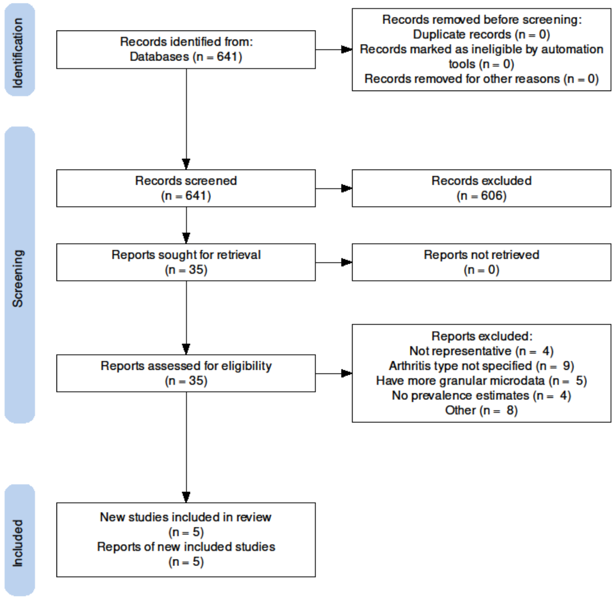

Supplemental Figure 1. Identification of new studies via databases and registers

Supplemental Table 1: MR-BRT crosswalk adjustment factors for rheumatoid arthritis

Data input	Reference or alternative case definition	Gamma	Beta coefficient, log (95% CI)	Adjustment factor*
ACR 1987	Ref	0.09	---	---
RA criteria other than RA 1987**	Alt		0.23 (–0.47 to 0.94)	1.26 (0.63 to 1.56)
USA claims data – 2000	Alt		0.69 (–0.46 to 1.84)	1.72 (0.63 to 6.30)

**Adjustment factor is the transformed beta coefficient in normal space and can be interpreted as the factor by which the alternative case definition is adjusted to reflect what it would have been if measured as the reference.*
*** Other criteria include ACR/EULAR 2010, American Rheumatism Association 1958, Rome 1961, and general physician diagnosis.*

Supplemental Table 2: Covariates. Summary of covariates used in the rheumatoid arthritis DisMod-MR metaregression model

Covariate	Type	Parameter	Exponentiated beta (95% uncertainty interval)
Healthcare Access and Quality Index	Country-level	Excess mortality rate	0.98 (0.98–0.98)
Mean BMI	Country-level	Prevalence	1.16 (1.12–1.18)

Supplemental Figure 2: Distribution of data sources for rheumatoid arthritis

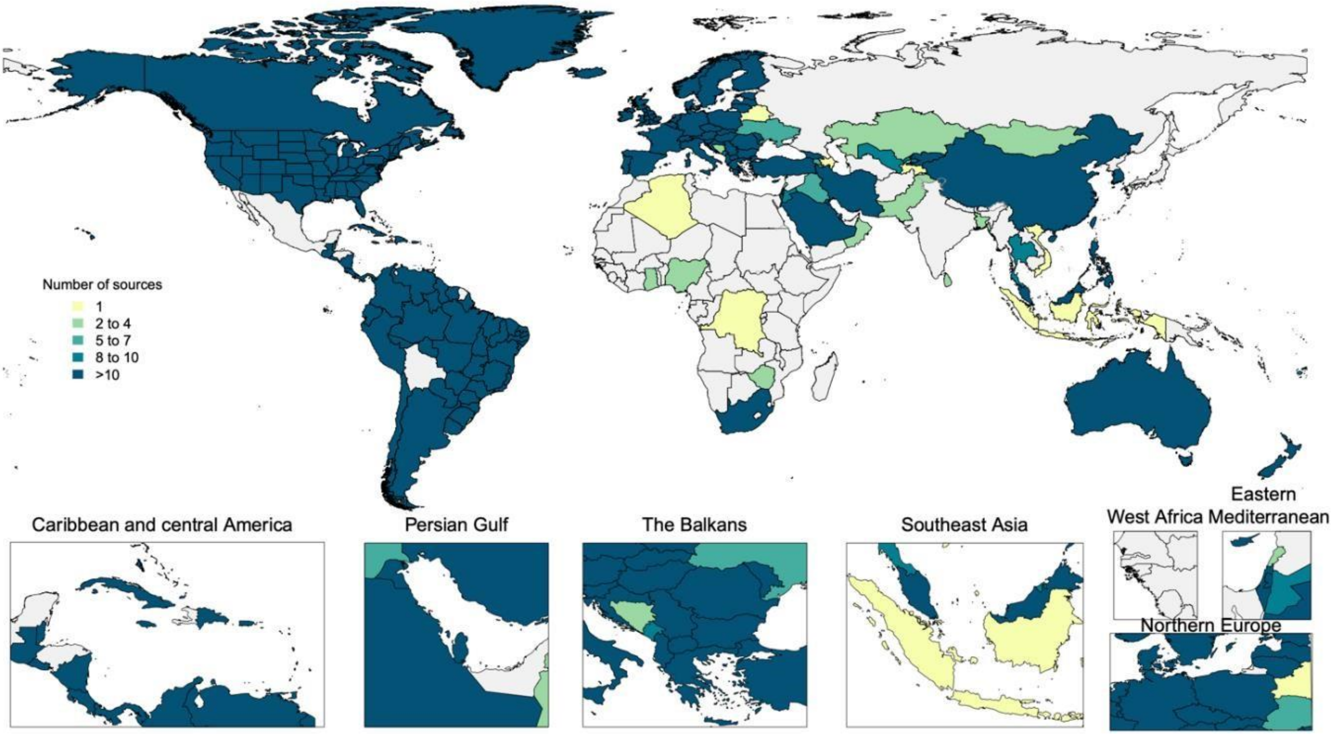

Supplemental Table 3: National and subnational sources in countries with subnational estimation

Location	National source counts	Subnational source counts	Total source counts
Brazil	0	870	870
China	0	832	832
Indonesia	1	0	1
India	0	11	11
Iran	0	436	436
Italy	3	801	804
Japan	0	1788	1788
Mexico	1	1250	1251
New Zealand	0	72	72
Nigeria	0	2	2
Norway	0	365	365
Pakistan	0	3	3
Philippines	1	868	869
Poland	0	592	592
Russia	0	2508	2508
South Africa	0	180	180
Sweden	4	71	75
UK	130	5706	5836
Ukraine	7	0	7
USA	4	4653	4657

Section 2. Excess mortality rate (EMR) modelling

In previous rounds, priors on excess mortality rate (EMR) were estimated in DisMod by matching prevalence datapoints with their corresponding CSMR values within the same age, sex, year, location (by dividing CSMR by prevalence). For short duration conditions (remission >1), the corresponding prevalence was derived by running an initial model and then applying the same CSMR/prevalence method. However, for many causes, DisMod estimated a rather unrealistic pattern of EMR compared to an expected pattern of decreasing EMR with greater access to quality health care. Such unexpected patterns often signal inconsistencies between CSMR estimates and the measures of prevalence and/or incidence. In an effort to provide greater guidance to DisMod on the expected pattern of EMR, EMR data generated in the previous round were modelled using the MR-BRT approach by age and sex with a prior on Healthcare Access and Quality (HAQ) Index having a negative coefficient. Results from MR-BRT were then predicted for each location year, sex, and for ages 0, 10, 20 ….100. We included HAQ Index as a country-level covariate to inform EMR with a mean and standard deviation produced from MR-BRT. However, even without this setting, DisMod would tend to estimate a coefficient that was consistent with the MRBRT analysis.

Supplemental Table 4: Covariates used in rheumatoid arthritis mortality modelling

Level	Covariate	Direction
1	Cumulative cigarettes (10 years)	+
	Cumulative cigarettes (5 years)	+
	Smoking prevalence	+
	Milk (g), unadjusted	+
	Healthcare Access and Quality Index	-
2	Mean BMI	+
	Mean cholesterol	+
3	Education (years per capita)	+
	Log-transformed LDI: lag-distributed income ($ per capita)	+

Section 3. Severity distribution meta-analysis

To determine the proportion of people with RA within each of the severity levels, seven studies from three regions provided information on the severity of RA. Severity was classified according to Health Assessment Questionnaire scores, with the cutoff for each severity level: <1 mild; 1–1.99 moderate; and ≥2 severe. Estimates were across studies. We used a random effects meta-analysis model. The pooled percentages were mild 48.8% (37.9–59.6), moderate 37.6% (29.3–46.2), and severe 12.2% (7.8–17.4).

Supplemental Table 5: Severity distribution, details on the severity levels for rheumatoid arthritis in GBD 2021 and the associated disability weight (DW) with that severity

Severity level	Lay description	DW (95% CI)
Mild	This person has moderate pain and stiffness in the arms and hands which causes difficulty lifting, carrying, and holding things, and trouble sleeping because of the pain.	0.117 (0.080–0.163)
Moderate	This person has pain and deformity in most joints, causing difficulty moving around, getting up and down, and using the hands for lifting and carrying. The person often feels fatigue.	0.317 (0.216–0.440)
Severe	This person has severe, constant pain, and deformity in most joints, causing difficulty moving around, getting up and down, eating, dressing, lifting, carrying, and using the hands. The person often feels sadness, anxiety, and extreme fatigue.	0.581 (0.403–0.739)

Supplemental Figure 3a. Severity distribution meta-analysis of mild rheumatoid arthritis

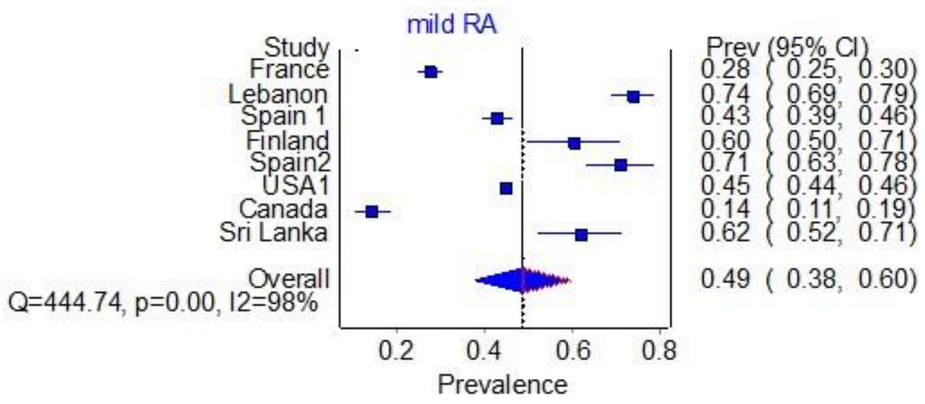

Details of the studies/locations included in the meta-analysis calculating the proportion of mild RA.

Supplemental Figure 3b. Severity distribution meta-analysis of moderate rheumatoid arthritis

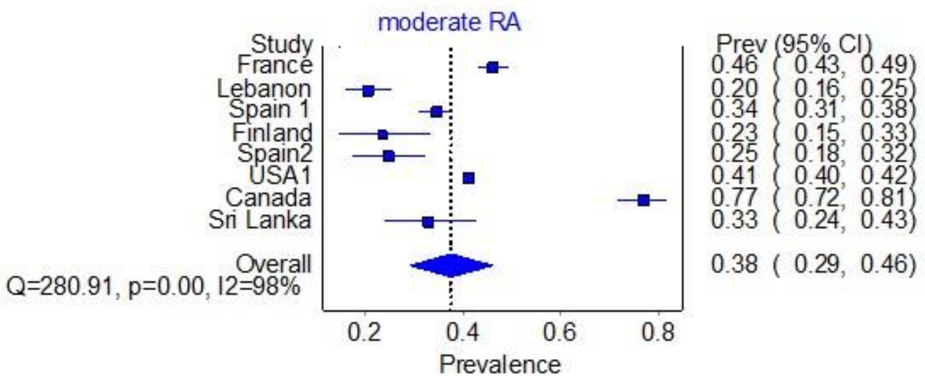

Details of the studies/locations included in the meta-analysis calculating the proportion of moderate RA.

Supplemental Figure 3c. Severity distribution meta-analysis of severe rheumatoid arthritis

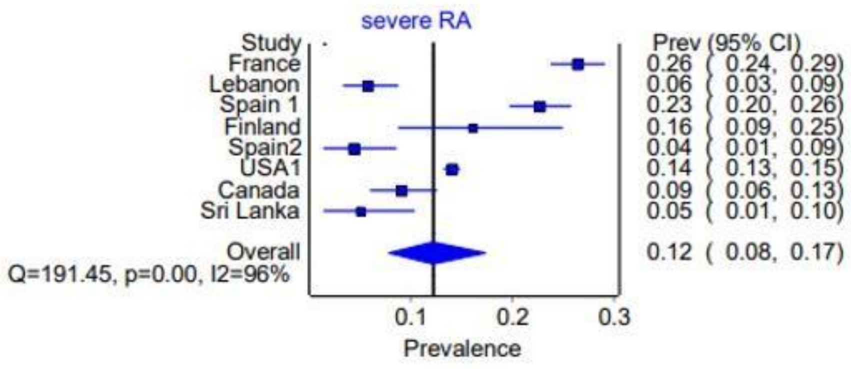

Details of the studies/locations included in the meta-analysis calculating the proportion of severe RA.

Supplemental Table 6: Rheumatoid arthritis prevalence and age-standardised DALY rate by super-region, region, and country

Location	Number of prevalent cases (95% UI)	Agestandardised prevalence rate per 100 000 (95% UI)	% change in age-standardised prevalence rate from 1990 to 2020 (95% UI)	Agestandardised rate of YLDs per 100 000 in 2020 (95% UI)	% change in age-standardised rate of YLDs per 100 000 from 1990 to 2020 (95% UI)	Agestandardised rate of YLLs per 100 000 (95% UI)	Agestandardised rate of DALYs per 100 000 (95% UI)	% change in age-standardised rate of DALYs per 100 000 from 1990 to 2020 (95% UI)
Global	17 600 000 (15 800 000–20 300 000)	208·8 (186·8–241·1)	14·1 (12·7–15·4)	27·8 (18·9–37·1)	13·8 (12·3–15·6)	8·6 (7·7–9·9)	36·4 (27·6–45·9)	-1·0 (-5·5–2·2)
**Central Europe, eastern Europe, and central Asia**	**1 030 000 (914 000–1 180 000)**	**186·9 (162·8–215·7)**	**24·2 (21·7–26·7)**	**25·2 (17·1–33·9)**	**24·5 (20·4–29·0)**	**7·3 (6·7–7·8)**	**32·4 (24·2–40·9)**	**8·1 (2·7–12·5)**
**Central Asia**	**171 000 (149 000–197 000)**	**182·7 (160·0–208·0)**	**41·7 (36·9–47·6)**	**24·9 (17·0–33·3)**	**41·5 (32·1–50·9)**	**4·1 (3·7–4·6)**	**28·9 (21·1–37·3)**	**59·0 (48·4–71·5)**
Armenia	4370 (3730–5270)	114·9 (96·1–139·3)	51·5 (42·4–70·1)	15·7 (10·4–22·0)	50·7 (25·8–79·2)	1·9 (1·7–2·1)	17·6 (12·4–23·8)	67·0 (40·8–95·0)
Azerbaijan	10 000 (8370–12 200)	85·2 (71·5–103·3)	27·4 (19·9–35·1)	11·8 (7·6–16·3)	26·2 (11·9–40·9)	0·3 (0·1–0·4)	12·0 (7·9–16·5)	28·5 (14·5–45·2)
Georgia	4630 (3900–5520)	97·7 (81·1–117·3)	18·5 (11·3–24·7)	13·2 (8·7–18·7)	16·8 (0·5–36·8)	1·0 (0·9–1·1)	14·2 (9·6–19·5)	24·6 (7·3–46·3)
Kazakhstan	20 500 (17 300–25 200)	104·7 (88·2–127·5)	37·9 (28·8–48·3)	14·2 (9·4–20·0)	37·0 (17·7–59·1)	4·1 (2·9–5·2)	18·3 (13·5–24·2)	73·6 (50·0–118·3)
Kyrgyzstan	18 500 (16 300–20 700)	330·7 (295·8–368·0)	45·9 (36·4–54·4)	44·4 (30·0–62·9)	45·6 (28·8–61·9)	9·2 (7·3–11·7)	53·5 (39·0–71·7)	50·3 (36·1–69·0)
Mongolia	4790 (4090–5710)	144·8 (125·3–168·3)	49·9 (41·5–62·6)	19·6 (13·2–27·4)	49·2 (29·2–74·5)	7·3 (4·5–11·7)	27·0 (19·4–34·9)	56·3 (4·2–94·6)
Tajikistan	11 400 (9630–13 000)	148·1 (128·3–166·7)	27·7 (19·4–36·5)	20·2 (13·1–28·4)	28·4 (14·5–44·5)	2·8 (1·1–4·4)	23·0 (15·7–31·1)	41·0 (20·1–61·5)
Turkmenistan	4380 (3690–5290)	88·0 (74·8–105·3)	34·5 (26·4–44·4)	12·2 (8·1–16·4)	33·9 (16·7–62·3)	1·6 (1·2–2·2)	13·8 (9·7–18·0)	51·4 (34·9–80·5)
Uzbekistan	92 800 (81 000–105 000)	295·0 (260·8–329·1)	23·0 (15·5–31·4)	40·1 (27·3–54·5)	22·9 (9·2–35·5)	7·7 (6·7–9·0)	47·8 (34·7–62·1)	45·2 (28·9–61·3)
**Central Europe**	**349 000 (313 000–397 000)**	**202·1 (177·6–232·9)**	**20·0 (17·7–22·7)**	**27·2 (18·3–37·2)**	**20·6 (16·0–24·1)**	**4·5 (3·9–5·0)**	**31·7 (22·8–42·1)**	**-8·5 (-16·6–-2·4)**
Albania	5780 (5030–6740)	156·0 (134·2–183·0)	64·6 (54·6–76·8)	21·0 (14·7–28·6)	63·1 (42·5–85·9)	3·4 (2·1–5·3)	24·5 (17·0–32·0)	19·5 (-1·1–40·5)

Bosnia and Herzegovina	12 500 (11 000–13 800)	247·7 (218·2–275·5)	46·5 (36·9–54·4)	33·2 (22·4–45·6)	46·2 (27·6–65·9)	4·9 (3·5–6·9)	38·1 (27·1–49·7)	26·6 (9·4–44·0)
Bulgaria	12 900 (11 300–15 200)	122·6 (104·7–145·3)	24·8 (17·1–34·4)	16·7 (10·8–23·1)	24·9 (10·6–44·7)	1·5 (1·2–1·8)	18·2 (12·3–24·8)	12·1 (0·3–27·9)
Croatia	19 000 (16 900–21 100)	271·3 (237·6–309·8)	55·1 (42·9–69·0)	36·2 (24·0–49·6)	54·8 (37·9–73·7)	5·4 (4·3–6·2)	41·6 (29·5–54·7)	27·2 (14·4–39·4)
Czechia	29 400 (26 100–34 200)	187·5 (161·3–219·2)	38·7 (32·0–46·1)	25·3 (16·6–35·4)	38·7 (20·6–58·1)	2·7 (2·2–3·2)	27·9 (19·2–38·0)	18·7 (1·6–31·6)
Hungary	35 200 (32 100–38 600)	227·4 (203·8–255·0)	24·1 (15·9–31·0)	30·5 (20·7–41·9)	25·6 (12·6–38·3)	6·3 (4·9–7·7)	36·8 (27·4–48·4)	-4·9 (-17·0–6·8)
Montenegro	1390 (1220–1640)	162·1 (140·3–192·2)	24·2 (18·9–30·8)	21·9 (14·0–29·4)	23·8 (7·6–41·0)	3·6 (2·7–4·8)	25·4 (17·5–33·1)	17·3 (2·2–32·7)
North Macedonia	3730 (3250–4480)	125·3 (107·4–151·2)	39·6 (31·6–48·7)	17·0 (11·1–23·5)	38·2 (21·8–57·5)	0·9 (0·6–1·2)	17·8 (12·0–24·7)	27·4 (9·6–43·5)
Poland	148 000 (131 000–170 000)	256·7 (224·1–296·4)	-6·6 (-10·6–-3·5)	34·5 (23·1–47·0)	-5·8 (-11·2–-1·5)	7·9 (6·6–8·8)	42·4 (31·1–55·3)	-34·0 (-40·8–-28·0)
Romania	37 300 (32 400–44 700)	130·8 (110·9–158·6)	49·7 (41·5–58·8)	17·8 (12·3–24·7)	49·3 (34·3–69·7)	0·0 (0·0–0·1)	17·8 (12·3–24·8)	48·9 (34·1–69·1)
Serbia	22 000 (19 800–25 200)	167·6 (145·9–193·4)	52·2 (40·6–67·1)	22·4 (15·3–30·5)	50·3 (31·0–69·7)	4·2 (3·2–5·6)	26·6 (18·9–34·9)	25·3 (9·9–45·7)
Slovakia	11 000 (9 730–12 700)	138·9 (120·1–162·5)	39·3 (29·2–50·3)	18·9 (12·3–25·6)	38·8 (21·4–59·2)	3·0 (2·2–4·0)	21·9 (15·0–28·6)	13·1 (-1·0–26·1)
Slovenia	11 200 (10 200–12 600)	314·2 (281·0–356·6)	40·6 (30·9–52·8)	41·7 (28·3–56·8)	40·6 (27·1–59·6)	6·9 (5·1–8·7)	48·6 (34·9–62·7)	1·1 (-11·3–10·5)
**Eastern Europe**	**514 000 (450 000–588 000)**	**176·6 (152·3–204·0)**	**22·0 (19·1–25·3)**	**23·7 (15·9–31·8)**	**22·1 (18·3–26·4)**	**9·7 (8·9–10·5)**	**33·4 (25·5–41·3)**	**12·7 (8·1–17·1)**
Belarus	17 400 (15 300–20 800)	127·2 (109·7–154·3)	53·2 (39·6–64·9)	17·1 (11·3–23·2)	53·0 (32·7–74·5)	0·6 (0·4–0·7)	17·7 (11·9–23·8)	51·3 (31·6–71·4)
Estonia	7110 (6520–7710)	360·3 (323·2–396·8)	28·7 (19·3–38·6)	48·3 (33·7–64·2)	29·2 (18·2–43·8)	17·4 (14·3–20·9)	65·7 (50·6–80·9)	10·7 (0·3–23·0)
Latvia	7720 (6990–8520)	266·5 (236·6–302·2)	24·7 (16·7–36·0)	35·8 (24·1–48·1)	25·1 (10·3–42·4)	15·3 (12·5–18·3)	51·1 (40·1–63·8)	12·6 (0·4–25·8)
Lithuania	13 300 (12 200–14 500)	315·0 (284·3–347·3)	20·0 (10·8–28·8)	42·1 (28·3–57·5)	19·7 (7·6–35·2)	21·9 (18·0–25·3)	64·0 (50·7–79·5)	15·7 (4·2–29·5)
Republic of Moldova	6420 (5580–7460)	124·7 (106·4–144·7)	43·6 (33·1–57·4)	16·9 (11·5–23·9)	43·0 (22·6–63·3)	5·5 (4·8–6·4)	22·4 (16·7–29·9)	41·8 (26·2–57·5)
Russia	403 000 (352 000–458 000)	200·5 (172·7–229·9)	17·5 (14·5–20·9)	26·9 (18·0–35·9)	17·6 (13·9–21·3)	11·2 (10·1–12·3)	38·1 (29·1–47·2)	8·2 (2·7–12·3)

Ukraine	59 500 (51 400–71 900)	95·3 (79·8–116·5)	27·4 (20·5–34·3)	12·9 (8·7–18·1)	27·8 (10·6–47·9)	6·2 (4·6–7·6)	19·1 (14·7–24·1)	20·3 (3·4–39·5)
**HIGH INCOME**	**4 920 000 (4 520 000–5 450 000)**	**288·1 (262·6–324·0)**	**11·3 (9·6–12·8)**	**38·3 (26·3–50·2)**	**10·8 (9·3–12·6)**	**7·1 (6·4–7·5)**	**45·4 (33·5–57·6)**	**-5·7 (-10·5–-2·6)**
**Australasia**	**158 000 (143 000–178 000)**	**355·6 (318·1–406·9)**	**13·0 (7·2–18·4)**	**47·1 (32·1–63·3)**	**13·3 (4·2–20·7)**	**8·8 (7·8–9·5)**	**55·9 (41·1–72·6)**	**-5·7 (-13·9–-0·7)**
Australia	129 000 (117 000–145 000)	347·2 (309·7–396·4)	17·0 (9·8–24·3)	46·0 (31·2–62·1)	17·1 (6·2–26·3)	8·7 (7·7–9·5)	54·6 (40·1–71·1)	-3·1 (-12·7–3·2)
New Zealand	29 000 (26 000–33 300)	397·9 (352·9–460·3)	-1·8 (-6·5–4·0)	52·8 (36·0–70·9)	-1·0 (-8·0–5·0)	9·6 (8·3–10·6)	62·3 (45·5–80·8)	-15·9 (-24·2–-11·2)
**High income Asia Pacific**	**910 000 (810 000–1 040 000)**	**266·9 (236·7–312·9)**	**-8·1 (-11·1–-5·1)**	**36·1 (24·1–48·9)**	**-7·5 (-11·0–-3·5)**	**7·1 (6·1–7·8)**	**43·3 (31·4–56·4)**	**-23·8 (-29·3–-19·6)**
Brunei	962 (841–1130)	230·6 (205·6–264·8)	29·0 (22·9–36·6)	30·8 (21·3–41·7)	27·9 (16·3–41·1)	20·5 (14·9–26·5)	51·3 (38·5–63·6)	-3·8 (-19·4–12·9)
Japan	696 000 (621 000–799 000)	281·8 (245·9–335·7)	-10·4 (-13·2–-7·0)	38·2 (25·1–51·9)	-9·7 (-12·5–-6·2)	8·2 (7·1–8·8)	46·3 (33·6–60·3)	-23·6 (-28·0–-19·5)
South Korea	202 000 (183 000–227 000)	236·0 (213·5–267·0)	40·7 (35·1–47·1)	31·9 (21·9–43·0)	39·7 (25·3–54·5)	3·8 (2·9–5·2)	35·7 (25·4–47·9)	-5·0 (-21·5–7·2)
Singapore	12 000 (10 100–14 300)	147·8 (123·6–175·2)	33·1 (26·4–40·9)	20·4 (13·6–28·0)	34·6 (18·4–54·2)	0·9 (0·8–1·1)	21·3 (14·7–29·0)	15·1 (1·7–30·2)
**High income North America**	**1 620 000 (1 490 000–1 750 000)**	**300·5 (278·5–322·3)**	**18·2 (16·7–19·6)**	**39·3 (27·5–51·8)**	**16·7 (14·3–20·1)**	**7·0 (6·3–7·4)**	**46·3 (34·4–58·9)**	**4·3 (0·8–7·4)**
Canada	193 000 (187 000–198 000)	332·5 (324·1–341·1)	34·6 (31·2–37·2)	44·4 (29·8–58·6)	33·9 (23·9–44·9)	7·5 (6·7–8·2)	51·9 (37·0–66·1)	14·4 (5·1–22·4)
Greenland	157 (140–178)	217·8 (194·3–247·6)	57·1 (47·3–65·8)	29·1 (19·4–38·3)	57·4 (38·9–80·1)	8·8 (6·0–14·6)	37·9 (26·8–49·8)	37·9 (23·4–54·7)
USA	1 440 000 (1 310 000–1 560 000)	296·8 (272·0–321·0)	16·4 (14·6–17·7)	38·7 (27·1–51·1)	14·8 (12·3–18·2)	6·9 (6·2–7·3)	45·6 (34·0–57·9)	3·0 (-0·2–5·7)
**Southern Latin America**	**210 000 (187 000–237 000)**	**266·1 (235·3–302·1)**	**62·0 (54·5–72·3)**	**35·5 (23·5–48·1)**	**61·5 (49·7–73·6)**	**10·0 (9·2–10·7)**	**45·5 (33·5–58·0)**	**32·9 (22·5–41·9)**
Argentina	112 000 (97 800–128 000)	219·4 (190·8–251·3)	48·5 (39·8–61·2)	29·4 (19·5–39·9)	48·7 (34·4–64·9)	8·7 (8·0–9·4)	38·2 (28·3–49·0)	31·0 (20·0–42·6)
Chile	88 200 (79 300–97 800)	377·5 (336·2–420·4)	73·8 (62·3–87·0)	50·0 (33·3–67·3)	72·6 (57·7–89·1)	11·7 (10·2–12·7)	61·7 (45·2–78·9)	23·5 (10·6–34·8)
Uruguay	9890 (8700–11 200)	218·3 (189·9–252·8)	40·7 (32·4–47·5)	29·2 (19·3–39·8)	40·4 (26·9–55·4)	15·4 (14·2–16·6)	44·5 (34·4–54·9)	28·9 (19·4–38·8)
**Western Europe**	**2 020 000 (1 840 000–2 290 000)**	**284·9 (252·4–328·7)**	**11·0 (9·3–12·5)**	**38·0 (25·7–50·5)**	**10·9 (8·4–13·3)**	**6·5 (5·9–7·0)**	**44·5 (32·4–57·2)**	**-7·1 (-12·8–-2·9)**

Andorra	210 (179–249)	168·5 (142·6–197·8)	13·5 (7·5–19·2)	22·8 (14·9–31·5)	12·0 (-0·8–26·8)	0·8 (0·6–1·1)	23·6 (15·7–32·3)	8·2 (-3·5–21·2)
Austria	37 700 (33 600–43 000)	270·7 (234·2–311·2)	4·8 (-0·5–10·7)	36·3 (23·9–49·8)	5·1 (-6·6–15·6)	4·5 (4·0–5·0)	40·9 (28·4–54·0)	-11·0 (-20·5–-3·6)
Belgium	46 400 (41 000–53 200)	263·3 (226·3–308·2)	13·1 (7·7–18·6)	35·1 (23·7–46·9)	12·7 (1·8–22·2)	5·2 (4·4–5·9)	40·3 (29·0–52·0)	0·1 (-7·7–7·8)
Cyprus	7170 (6440–8100)	379·4 (336·4–433·1)	27·1 (20·1–34·5)	50·3 (33·6–67·3)	26·4 (17·9–37·3)	12·3 (9·5–15·5)	62·7 (44·9–80·2)	-8·1 (-26·4–3·6)
Denmark	35 800 (32 800–39 500)	356·8 (319·3–402·7)	34·2 (26·5–42·1)	47·4 (32·3–62·3)	35·3 (23·3–48·8)	9·3 (8·1–10·5)	56·7 (41·7–71·9)	18·8 (8·7–30·9)
Finland	45 000 (40 600–49 500)	457·8 (412·4–513·6)	25·5 (20·0–34·1)	60·8 (41·2–83·3)	25·5 (14·4–37·4)	14·8 (13·3–16·0)	75·6 (56·0–98·7)	-8·2 (-17·6–3·3)
France	235 000 (212 000–268 000)	218·0 (192·1–253·2)	21·5 (14·0–27·4)	28·9 (19·8–38·5)	21·6 (11·9–31·8)	4·7 (4·1–5·3)	33·6 (24·4–43·3)	-1·1 (-10·4–9·3)
Germany	327 000 (281 000–381 000)	237·4 (197·5–279·0)	17·0 (9·8–23·7)	31·9 (20·9–42·5)	16·4 (4·7–26·0)	4·7 (4·1–5·3)	36·6 (25·8–47·6)	1·8 (-7·5–10·8)
Greece	35 800 (31 100–40 900)	226·0 (194·5–256·2)	17·2 (12·2–23·7)	30·6 (20·0–40·8)	17·2 (6·4–29·1)	3·0 (2·7–3·3)	33·6 (23·1–44·0)	15·4 (5·7–25·6)
Iceland	1300 (1140–1470)	274·5 (235·3–315·4)	16·6 (11·0–23·2)	37·1 (24·7–50·0)	16·8 (4·8–31·6)	4·6 (4·0–5·2)	41·7 (29·2–54·6)	1·4 (-7·1–11·4)
Ireland	37 800 (34 700–41 900)	538·7 (485·3–601·1)	21·5 (14·5–28·3)	71·4 (47·6–95·7)	20·9 (12·3–29·8)	13·9 (11·8–15·3)	85·3 (61·7–109·2)	-2·8 (-13·7–4·0)
Israel	22 200 (19 300–26 300)	212·5 (183·1–253·7)	28·1 (19·8–36·9)	28·6 (18·7–38·5)	26·0 (10·3–44·7)	3·2 (2·8–3·5)	31·7 (22·1–41·8)	17·2 (4·5–31·8)
Italy	263 000 (230 000–304 000)	241·2 (209·4–288·3)	-1·8 (-4·1–0·2)	32·2 (21·4–43·6)	-1·3 (-4·2–1·4)	6·3 (5·7–6·7)	38·5 (28·0–50·0)	-6·6 (-10·0–-4·2)
Luxembourg	2130 (1890–2450)	238·2 (209·4–276·8)	16·4 (10·5–21·9)	31·8 (20·7–42·6)	15·8 (5·0–26·5)	4·0 (3·5–4·5)	35·7 (24·5–46·3)	-0·9 (-9·6–7·1)
Malta	1590 (1400–1840)	226·1 (193·8–266·1)	18·3 (11·9–27·3)	30·5 (19·6–41·0)	17·6 (5·6–31·3)	4·2 (3·5–4·8)	34·7 (24·0–45·0)	1·1 (-10·1–11·2)
Monaco	101 (87.1–118)	170·5 (144·2–202·2)	10·0 (5·4–16·5)	23·2 (15·5–31·1)	9·6 (-2·6–20·3)	0·4 (0·3–0·6)	23·6 (15·9–31·5)	8·9 (-2·9–19·3)
Netherlands	109 000 (97 900–122 000)	375·2 (332·1–425·9)	2·9 (-2·3–8·5)	49·7 (33·8–67·0)	2·3 (-6·5–13·2)	10·3 (8·9–11·2)	60·0 (44·5–77·7)	-16·7 (-24·6–-8·7)
Norway	32 200 (28 200–36 800)	382·5 (336·3–445·6)	-11·5 (-15·5–-6·9)	50·8 (33·8–66·1)	-10·8 (-14·7–-5·9)	8·0 (7·0–8·8)	58·8 (42·0–74·4)	-29·7 (-35·9–-24·2)
Portugal	46 900 (42 300–53 500)	270·4 (239·7–313·9)	45·0 (34·3–54·3)	36·2 (24·0–48·0)	44·7 (26·7–60·2)	5·9 (4·9–6·6)	42·2 (30·2–54·3)	17·6 (4·4–29·2)

San Marino	74.8 (62.8–88.2)	162·3 (132·7–193·3)	9·9 (5·2–14·3)	22·1 (14·2–30·2)	11·2 (-0·0–23·0)	2·1 (1·5–3·0)	24·3 (16·5–32·4)	7·9 (-2·5–19·2)
Spain	177 000 (165 000–188 000)	233·5 (216·5–248·5)	20·5 (13·8–25·9)	31·2 (21·2–40·4)	20·0 (9·4–29·4)	4·1 (3·4–4·7)	35·3 (25·3–44·8)	1·9 (-7·8–10·3)
Sweden	55 600 (50 000–62 200)	327·8 (294·8–371·6)	-10·3 (-13·3–-7·1)	43·9 (29·2–58·2)	-10·1 (-15·7–-5·9)	7·4 (6·7–8·2)	51·3 (37·0–66·0)	-20·9 (-26·0–-16·1)
Switzerland	43 600 (38 900–50 100)	300·2 (262·1–353·7)	9·8 (4·5–15·1)	39·9 (26·2–53·5)	9·4 (1·8–19·4)	4·9 (4·1–5·5)	44·8 (31·4–58·8)	-6·3 (-14·1–1·2)
UK	458 000 (414 000–512 000)	446·7 (405·1–509·7)	7·7 (6·4–8·9)	59·5 (40·4–78·2)	7·4 (6·0–8·9)	12·3 (11·2–13·0)	71·8 (53·1–90·8)	-16·2 (-22·4–-11·8)
**Latin America and Caribbean**	**1 690 000 (1 500 000–1 930 000)**	**269·7 (238·8–306·6)**	**24·2 (21·2–26·5)**	**36·1 (24·5–48·7)**	**24·0 (20·7–27·8)**	**12·7 (11·5–13·7)**	**48·7 (37·0–61·4)**	**8·2 (3·6–12·6)**
**Andean Latin America**	**268 000 (241 000–301 000)**	**427·8 (385·9–479·2)**	**63·1 (54·8–70·6)**	**57·5 (38·3–77·5)**	**61·6 (50·5–73·6)**	**10·7 (8·8–12·2)**	**68·2 (48·5–87·9)**	**34·5 (20·9–45·0)**
Bolivia	31 700 (27 500–36 100)	305·9 (267·9–345·4)	59·2 (48·0–69·3)	40·7 (27·7–55·3)	57·6 (41·6–75·3)	16·8 (13·0–22·2)	57·4 (44·1–71·8)	22·7 (4·1–40·9)
Ecuador	54 100 (47 100–60 900)	326·9 (286·7–366·1)	63·2 (53·3–75·0)	43·5 (28·5–59·0)	61·6 (47·8–79·0)	13·0 (9·6–15·3)	56·5 (41·3–72·0)	21·9 (7·2–33·4)
Peru	182 000 (160 000–207 000)	517·8 (456·6–586·6)	66·7 (54·7–78·0)	69·9 (46·7–94·4)	65·3 (52·1–83·6)	8·4 (6·6–10·7)	78·3 (54·0–102·4)	45·1 (27·7–58·8)
Caribbean	79 800 (70 300–93 800)	153·4 (134·9–180·5)	32·3 (27·4–36·7)	20·5 (14·0–27·5)	30·8 (23·4–40·6)	8·6 (7·4–10·6)	29·1 (22·0–37·3)	14·2 (5·8–21·9)
Antigua and Barbuda	154 (133–182)	146·9 (127·0–172·2)	38·7 (28·4–47·3)	19·6 (12·7–26·9)	36·2 (20·0–57·3)	8·3 (7·2–9·1)	27·9 (20·8–34·9)	33·8 (20·6–50·7)
Bahamas	647 (553–775)	151·7 (130·6–180·8)	27·9 (19·7–36·7)	20·3 (14·0–28·3)	27·1 (12·2–47·8)	8·4 (7·0–9·7)	28·7 (22·2–37·1)	9·8 (-0·0–24·2)
Barbados	977 (875–1110)	224·6 (196·7–259·4)	36·6 (28·5–44·3)	30·1 (19·7–41·5)	36·2 (20·2–54·1)	13·8 (11·5–16·9)	43·9 (33·6–55·4)	29·3 (16·6–46·0)
Belize	558 (466–651)	155·0 (132·5–178·5)	39·7 (31·8–49·5)	20·9 (12·8–28·1)	38·7 (21·6–57·9)	8·4 (6·6–9·8)	29·3 (21·5–36·3)	33·0 (15·8–49·9)
Bermuda	191 (169–224)	180·2 (154·7–217·2)	36·5 (28·5–43·3)	24·1 (16·1–32·2)	35·3 (22·3–50·6)	4·8 (3·9–5·7)	28·9 (20·6–37·1)	16·5 (4·6–28·2)
Cuba	33 500 (29 800–39 100)	204·3 (177·8–242·2)	41·9 (29·9–52·2)	27·5 (19·0–37·6)	41·4 (27·0–61·0)	8·7 (7·5–10·2)	36·2 (27·5–47·1)	28·1 (14·9–41·1)
Dominica	110 (94.9–126)	133·1 (113·7–153·0)	24·2 (16·6–31·9)	17·9 (11·9–25·0)	23·4 (8·9–40·0)	8·6 (6·7–10·8)	26·5 (19·9–33·4)	18·8 (7·1–31·4)
Dominican Republic	10 000 (8490–12 000)	97·6 (83·6–115·8)	29·8 (25·0–34·5)	13·0 (8·7–17·0)	28·0 (11·2–48·5)	7·3 (5·3–9·3)	20·3 (15·5–25·3)	-2·4 (-22·2–13·8)

Grenada	121 (102–143)	105·8 (89·3–124·7)	50·4 (41·0–59·3)	14·2 (9·5–18·9)	47·9 (31·7–74·7)	3·7 (3·2–4·2)	18·0 (13·2–22·9)	31·1 (18·9–49·8)
Guyana	631 (529–749)	85·9 (72·7–101·3)	35·3 (28·5–43·9)	11·5 (7·7–15·7)	33·2 (15·8–54·9)	3·8 (3·0–4·8)	15·3 (11·5–19·6)	69·6 (45·9–103·8)
Haiti	9610 (7940–11 400)	98·9 (84·2–114·3)	24·9 (17·7–31·0)	13·1 (8·9–18·3)	22·7 (7·9–37·9)	12·5 (7·1–21·0)	25·7 (18·2–36·6)	-4·0 (-17·6–15·3)
Jamaica	3950 (3380–4560)	129·2 (110·6–149·1)	40·3 (33·0–50·9)	17·5 (11·4–24·2)	38·8 (24·5–58·2)	6·3 (5·0–8·1)	23·8 (17·7–29·8)	35·3 (20·5–50·2)
Puerto Rico	11 600 (10 100–13 400)	216·6 (185·8–256·7)	56·5 (47·0–68·7)	29·0 (18·9–40·0)	54·4 (36·9–73·4)	6·0 (5·1–6·9)	35·0 (25·0–46·0)	27·9 (13·4–40·3)
Saint Kitts and Nevis	122 (107–141)	171·2 (150·9–196·0)	41·1 (31·5–50·7)	22·9 (14·8–30·4)	40·8 (21·6–58·7)	12·9 (10·4–15·0)	35·7 (27·5–43·7)	15·2 (0·6–31·9)
Saint Lucia	333 (290–385)	154·2 (134·7–178·4)	36·8 (29·9–44·7)	20·5 (13·6–27·4)	35·6 (19·3–57·9)	9·4 (7·7–11·1)	29·9 (22·7–37·6)	13·7 (2·8–27·1)
Saint Vincent and the Grenadines	127 (108–153)	96·7 (81·7–116·5)	28·4 (21·2–36·3)	13·0 (8·8–18·7)	28·0 (7·5–43·9)	3·8 (3·3–4·3)	16·8 (12·3–22·8)	8·5 (-5·8–21·8)
Suriname	530 (452–641)	84·9 (72·3–102·5)	28·0 (19·8–35·7)	11·5 (7·6–16·3)	26·4 (12·2–43·4)	2·5 (1·9–3·1)	14·0 (9·9–18·7)	16·2 (4·0–32·0)
Trinidad and Tobago	3690 (3270–4100)	208·1 (183·2–232·2)	33·8 (27·2–42·5)	27·7 (18·8–38·1)	33·1 (18·8–48·7)	14·4 (11·5–18·1)	42·2 (33·4–51·8)	4·6 (-7·0–17·0)
Virgin Islands	190 (164–222)	132·8 (112·1–157·9)	33·3 (26·8–40·9)	17·9 (11·6–24·1)	33·1 (18·2–51·1)	3·7 (2·9–5·2)	21·6 (15·5–28·5)	19·7 (2·0–37·0)
**Central Latin America**	**915 000 (809 000–1 020 000)**	**357·2 (316·8–398·0)**	**23·8 (20·6–26·9)**	**47·5 (32·9–64·1)**	**24·0 (19·8–28·6)**	**20·6 (18·2–23·2)**	**68·2 (53·1–84·8)**	**3·5 (-2·2–9·1)**
Colombia	129 000 (116 000–145 000)	241·3 (216·0–270·8)	51·2 (39·9–64·8)	32·6 (22·5–45·0)	51·1 (35·1–69·2)	10·1 (8·6–12·0)	42·8 (32·3–55·2)	10·7 (-2·6–23·0)
Costa Rica	19 300 (17 200–21 700)	359·8 (319·2–404·9)	63·6 (51·4–78·6)	47·9 (32·3–64·4)	61·4 (46·2–80·5)	13·8 (11·2–16·4)	61·6 (46·6–78·5)	39·7 (28·8–51·5)
El Salvador	9190 (7820–10 900)	150·8 (128·3–179·4)	57·3 (47·2–66·7)	20·2 (13·5–26·6)	55·9 (37·8–73·0)	4·5 (3·4–5·8)	24·7 (17·8–31·8)	35·1 (17·7–52·6)
Guatemala	27 000 (23 200–30 300)	197·2 (171·4–221·0)	79·4 (68·1–91·7)	26·2 (17·1–35·0)	77·4 (59·0–105·0)	12·9 (11·1–14·6)	39·0 (30·0–48·0)	35·1 (18·1–48·1)
Honduras	27 000 (24 200–29 900)	362·6 (329·6–399·5)	55·8 (47·4–67·1)	48·1 (32·2–63·8)	53·4 (39·4–73·6)	32·5 (24·3–46·3)	80·6 (60·3–100·7)	35·4 (10·0–58·5)
Mexico	584 000 (506 000–662 000)	453·0 (393·8–512·7)	13·2 (9·4–17·1)	60·0 (40·9–81·4)	13·4 (9·6–18·5)	28·0 (24·0–32·7)	88·0 (67·3–109·2)	-2·9 (-9·7–2·9)
Nicaragua	12 900 (11 400–15 000)	240·0 (214·3–274·3)	45·2 (38·4–52·3)	31·8 (21·3–41·9)	43·5 (30·1–57·1)	13·0 (10·6–16·0)	44·8 (33·5–54·8)	24·2 (12·3–37·4)

Panama	8060 (6980–9500)	186·6 (161·8–220·0)	36·5 (29·0–44·3)	24·9 (16·7–34·0)	34·6 (18·7–56·4)	7·9 (6·7–9·4)	32·8 (24·1–41·9)	20·7 (7·9–34·4)
Venezuela	98 100 (88 100–110 000)	315·9 (284·4–354·1)	43·3 (33·2–54·3)	42·3 (28·9–59·3)	42·3 (26·9–62·8)	18·4 (14·9–22·9)	60·7 (47·4–75·6)	19·4 (4·6–31·6)
**Tropical Latin America**	**430 000 (370 000–505 000)**	**169·0 (144·8–197·8)**	**0·8 (-2·0–3·9)**	**22·8 (15·0–32·3)**	**0·7 (-3·9–6·6)**	**6·2 (5·6–6·6)**	**28·9 (21·4–38·5)**	**-1·3 (-4·9–4·1)**
Brazil	410 000 (350 000–481 000)	165·6 (141·2–193·9)	-1·0 (-3·7–1·9)	22·3 (14·7–31·7)	-1·1 (-5·8–4·8)	5·9 (5·4–6·3)	28·2 (20·8–37·8)	-3·4 (-7·3–1·8)
Paraguay	20 100 (17 600–23 500)	306·9 (270·6–354·7)	68·9 (56·8–83·7)	41·2 (28·2–57·3)	67·2 (49·9–87·7)	16·0 (11·8–20·8)	57·2 (43·5–72·1)	72·0 (46·3–97·7)
**NORTH AFRICA AND MIDDLE EAST**	**683 000 (599 000–797 000)**	**116·8 (104·2–134·6)**	**50·1 (46·3–54·6)**	**15·7 (10·7–21·5)**	**48·2 (41·1–57·4)**	**3·0 (2·6–3·9)**	**18·8 (13·6–24·8)**	**27·8 (18·3–36·9)**
North Africa and Middle East	683 000 (599 000–797 000)	116·8 (104·2–134·6)	50·1 (46·3–54·6)	15·7 (10·7–21·5)	48·2 (41·1–57·4)	3·0 (2·6–3·9)	18·8 (13·6–24·8)	27·8 (18·3–36·9)
Afghanistan	16 700 (13 600–19 700)	67·9 (57·3–78·6)	25·4 (18·7–33·4)	9·1 (6·0–12·6)	23·1 (5·5–43·4)	8·7 (4·4–14·9)	17·8 (13·1–24·0)	34·3 (7·9–66·7)
Algeria	33 800 (28 300–40 600)	79·2 (66·7–94·6)	55·0 (45·7–66·4)	10·8 (7·0–14·5)	53·8 (25·6–78·4)	1·9 (1·4–2·5)	12·6 (8·8–16·4)	50·8 (30·0–74·3)
Bahrain	3620 (3030–4310)	203·2 (177·9–231·2)	67·2 (53·3–83·5)	27·0 (18·2–37·0)	64·3 (45·8–84·8)	4·2 (3·2–5·3)	31·3 (22·5–40·5)	81·0 (62·8–110·1)
Egypt	97 600 (80 600–116 000)	108·5 (90·8–128·2)	60·4 (50·8–72·2)	14·6 (9·8–19·8)	57·9 (34·2–84·2)	1·5 (1·2–1·9)	16·1 (11·3–21·3)	43·8 (25·1–65·8)
Iran	82 700 (69 100–100 000)	89·0 (75·5–107·0)	39·8 (35·6–44·5)	12·0 (8·0–16·5)	39·1 (31·2–46·7)	1·4 (1·2–1·8)	13·4 (9·5–18·0)	34·8 (26·2–43·2)
Iraq	37 500 (31 100–45 500)	104·5 (89·3–125·0)	39·7 (29·7–50·9)	14·0 (9·5–19·6)	38·5 (18·0–61·7)	0·7 (0·5–1·0)	14·8 (10·2–20·2)	29·4 (10·5–52·6)
Jordan	11 600 (9550–14 000)	108·5 (91·6–129·0)	52·3 (42·0–64·1)	14·6 (9·7–20·6)	50·5 (28·3–75·1)	1·0 (0·8–1·3)	15·6 (10·6–21·5)	43·3 (22·5–68·4)
Kuwait	13 200 (11 000–15 700)	233·7 (200·6–271·9)	63·0 (48·4–76·6)	31·4 (21·5–43·0)	60·8 (44·5–81·2)	4·2 (3·5–5·2)	35·7 (25·7–47·1)	74·5 (57·9–97·4)
Lebanon	9020 (7630–10 600)	163·8 (139·0–191·3)	46·5 (38·9–56·3)	21·7 (14·7–29·8)	44·4 (25·1–64·8)	1·1 (0·8–1·3)	22·8 (15·7–30·8)	29·0 (10·7–50·9)
Libya	7290 (6050–8760)	98·1 (82·5–116·3)	48·7 (40·9–59·4)	13·3 (8·7–18·8)	46·6 (27·7–67·5)	3·4 (2·3–4·8)	16·7 (12·1–22·7)	59·4 (40·9–84·5)
Morocco	33 600 (28 800–40 300)	88·9 (76·6–106·5)	50·2 (39·7–61·8)	11·9 (8·2–16·6)	46·0 (25·4–68·5)	3·7 (1·6–7·0)	15·6 (10·6–21·9)	48·5 (24·8–70·3)
Oman	3660 (2980–4570)	84·2 (71·8–101·8)	78·1 (69·6–88·5)	11·4 (7·5–16·0)	75·9 (54·4–103·3)	1·4 (0·8–3·0)	12·8 (8·7–17·8)	62·6 (40·2–90·9)

Palestine	3940 (3300–4750)	104·0 (87·8–124·7)	37·4 (28·0–45·2)	14·0 (9·3–20·3)	36·4 (16·4–58·6)	1·4 (1·1–1·8)	15·4 (10·6–21·7)	22·1 (5·6–39·2)
Qatar	3330 (2680–4240)	108·7 (91·1–132·7)	60·7 (52·1–73·6)	14·6 (9·7–20·0)	57·5 (43·3–74·6)	0·7 (0·5–1·1)	15·4 (10·3–21·0)	48·5 (35·3–63·2)
Saudi Arabia	37 800 (31 100–46 500)	94·7 (80·2–113·9)	73·5 (62·9–85·5)	12·7 (8·3–17·7)	69·2 (49·2–95·4)	1·7 (1·2–2·6)	14·4 (9·9–19·6)	61·5 (38·9–86·6)
Sudan	23 800 (19 500–28 700)	75·7 (64·0–91·0)	52·2 (43·3–62·6)	10·3 (6·8–14·0)	49·9 (27·0–76·4)	3·1 (2·1–5·1)	13·3 (9·5–18·4)	44·8 (22·7–75·2)
Syria	14 700 (12 300–18 000)	103·6 (86·5–126·5)	56·4 (45·0–69·0)	14·0 (8·9–20·5)	53·3 (32·4–80·2)	2·3 (1·7–3·1)	16·3 (11·3–23·0)	47·0 (27·8–70·6)
Tunisia	13 100 (11 000–15 800)	98·1 (82·0–118·5)	50·5 (40·6–61·9)	13·2 (8·7–18·4)	47·1 (27·6–68·8)	1·6 (0·8–2·3)	14·8 (10·2–19·5)	46·7 (26·5–68·5)
Türkiye	210 000 (189 000–231 000)	218·9 (197·5–240·5)	58·8 (50·3–71·6)	29·5 (20·1–40·7)	57·6 (40·7–76·0)	6·3 (4·8–8·5)	35·8 (26·7–46·3)	15·5 (-0·4–31·9)
United Arab Emirates	11 700 (9510–14 400)	100·1 (85·3–120·5)	49·4 (43·0–58·3)	13·7 (9·2–18·3)	49·3 (34·3–68·5)	2·5 (1·9–3·4)	16·2 (11·5–21·2)	15·9 (-1·1–36·1)
Yemen	13 700 (11 300–16 400)	59·9 (50·5–70·8)	29·5 (20·6–39·0)	8·2 (5·3–11·5)	29·4 (10·0–52·5)	3·1 (2·1–4·8)	11·3 (7·9–15·2)	35·5 (17·1–62·6)
**SOUTH ASIA**	**3 250 000 (2 850 000–3 820 000)**	**204·8 (181·7–238·1)**	**41·3 (37·8–44·5)**	**26·7 (18·2–35·5)**	**40·9 (37·2–44·7)**	**13·9 (10·4–19·9)**	**40·6 (31·2–49·6)**	**10·2 (0·4–20·1)**
**South Asia**	**3 250 000 (2 850 000–3 820 000)**	**204·8 (181·7–238·1)**	**41·3 (37·8–44·5)**	**26·7 (18·2–35·5)**	**40·9 (37·2–44·7)**	**13·9 (10·4–19·9)**	**40·6 (31·2–49·6)**	**10·2 (0·4–20·1)**
Bangladesh	212 000 (179 000–248 000)	138·0 (117·1–160·5)	57·5 (48·0–65·6)	18·5 (12·3–25·0)	55·4 (35·4–77·1)	10·1 (6·8–16·4)	28·5 (20·8–37·5)	2·5 (-17·2–23·2)
Bhutan	1400 (1200–1650)	208·5 (180·5–241·2)	54·4 (46·2–62·7)	27·7 (18·7–38·2)	54·3 (39·4–74·8)	14·3 (9·0–20·2)	42·0 (30·8–54·7)	14·7 (-2·5–36·0)
India	2 670 000 (2 350 000–3 140 000)	212·2 (186·9–246·8)	42·3 (38·5–45·6)	27·5 (18·8–36·7)	42·4 (38·4–45·8)	13·8 (10·4–19·7)	41·4 (31·7–50·8)	9·7 (-2·4–20·2)
Nepal	48 600 (41 000–57 100)	187·4 (161·3–217·9)	62·3 (55·8–71·5)	24·6 (16·5–33·1)	61·1 (41·1–80·8)	13·7 (8·9–21·6)	38·3 (28·6–48·6)	27·6 (3·8–55·8)
Pakistan	319 000 (273 000–380 000)	190·2 (165·6–225·5)	3·5 (0·0–7·8)	25·5 (16·8–34·3)	3·0 (-4·7–10·4)	19·6 (12·2–28·0)	45·1 (32·7–56·9)	8·4 (-7·0–24·6)
**SOUTHEAST ASIA, EAST ASIA, AND OCEANIA**	**5 360 000 (4 750 000–6 280 000)**	**195·2 (171·4–228·9)**	**15·4 (12·5–18·0)**	**26·4 (17·7–35·9)**	**15·7 (12·4–19·0)**	**8·8 (7·2–10·7)**	**35·2 (26·7–44·7)**	**1·1 (-8·3–9·1)**
East Asia	4 830 000 (4 290 000–5 630 000)	238·4 (208·8–279·4)	16·6 (13·2–19·5)	32·3 (21·6–44·1)	17·1 (13·7–20·9)	10·3 (8·5–12·6)	42·6 (32·4–54·4)	1·5 (-9·3–9·8)
China	4 700 000 (4 170 000–5 490 000)	240·3 (210·0–282·1)	16·6 (13·1–19·5)	32·6 (21·8–44·5)	17·2 (13·5–21·1)	10·3 (8·5–12·7)	42·9 (32·5–54·8)	1·5 (-9·6–10·2)

North Korea	68 900 (61 800–78 600)	207·9 (187·4–237·4)	30·7 (23·1–40·7)	28·2 (19·4–39·0)	30·0 (15·5–45·3)	15·7 (12·3–20·5)	44·0 (34·6–57·1)	18·5 (-2·7–38·3)
Taiwan (province of China)	63 300 (61 900–64 900)	166·7 (163·1–170·9)	3·1 (-8·3–18·6)	22·5 (15·7–29·6)	2·1 (-12·9–23·1)	6·9 (5·7–8·2)	29·4 (23·0–35·8)	-15·4 (-24·8–-3·7)
**Oceania**	**5460 (4470–6710)**	**50·5 (42·1–60·7)**	**18·0 (13·8–22·4)**	**7·0 (4·7–10·0)**	**16·3 (7·6–27·7)**	**0·0 (0·0–0·1)**	**7·1 (4·8–10·1)**	**16·2 (7·4–27·6)**
American Samoa	45.7 (38.1–55)	85·9 (72·0–103·7)	25·4 (18·9–34·5)	11·8 (7·7–16·3)	23·7 (7·8–45·7)	0·0 (0·0–0·1)	11·8 (7·8–16·4)	23·0 (7·3–44·2)
Cook Islands	15.5 (12.7–19.2)	73·2 (60·0–90·8)	41·8 (34·2–49·4)	10·0 (6·7–14·0)	38·2 (21·8–59·5)	0·0 (0·0–0·0)	10·0 (6·7–14·0)	38·2 (21·8–59·6)
Fiji	467 (376–573)	51·4 (41·6–62·9)	23·5 (17·9–30·1)	7·3 (4·6–10·0)	22·3 (7·1–38·4)	0·0 (0·0–0·0)	7·3 (4·6–10·0)	22·2 (7·0–38·2)
Guam	144 (120–178)	78·1 (65·0–97·0)	34·5 (25·5–42·8)	10·8 (7·1–15·7)	32·4 (14·8–52·9)	0·0 (0·0–0·0)	10·8 (7·1–15·7)	32·2 (14·6–52·6)
Kiribati	50 (41–61.2)	49·1 (41·2–59·2)	18·1 (12·7–24·7)	6·9 (4·5–9·7)	17·0 (3·7–31·6)	0·0 (0·0–0·0)	6·9 (4·5–9·7)	16·9 (3·6–31·5)
Marshall Islands	33.2 (27.4–39.7)	66·1 (55·8–78·3)	30·0 (22·9–37·3)	9·1 (6·0–12·7)	28·9 (11·1–46·4)	0·0 (0·0–0·1)	9·1 (6·1–12·7)	28·8 (11·3–46·5)
Micronesia (Federated States of)	73.8 (61.3–88.9)	79·9 (67·0–96·3)	41·2 (33·0–50·3)	11·0 (7·1–15·0)	39·8 (18·9–63·7)	0·1 (0·0–0·1)	11·1 (7·2–15·1)	39·5 (18·5–63·2)
Nauru	6.32 (5.13–7.71)	81·8 (69·4–97·7)	29·9 (22·9–35·5)	11·1 (7·3–15·5)	28·7 (10·5–49·6)	0·1 (0·0–0·1)	11·2 (7·3–15·6)	29·0 (10·9–49·6)
Niue	1.55 (1.33–1.85)	79·9 (68·2–95·3)	36·8 (29·0–43·3)	10·9 (6·8–15·5)	33·4 (16·1–52·5)	0·0 (0·0–0·0)	10·9 (6·8–15·5)	33·3 (16·1–52·2)
Northern Mariana Islands	47.9 (40.7–58.5)	85·9 (71·6–104·5)	17·7 (12·1–23·9)	11·8 (7·8–16·9)	17·2 (2·7–34·6)	0·3 (0·2–0·7)	12·3 (8·1–17·5)	18·2 (3·3–36·3)
Palau	20.6 (17.5–24.6)	87·5 (73·6–103·8)	31·5 (25·4–40·1)	11·9 (7·4–17·0)	29·2 (12·7–48·2)	0·1 (0·0–0·1)	12·0 (7·5–17·1)	28·5 (12·2–46·8)
Papua New Guinea	3620 (2940–4460)	46·1 (38·4–55·6)	17·6 (12·1–23·3)	6·4 (4·3–9·3)	15·8 (4·3–31·1)	0·0 (0·0–0·1)	6·5 (4·3–9·3)	15·7 (4·2–30·9)
Samoa	137 (112–165)	76·5 (63·5–91·8)	24·3 (18·0–32·1)	10·6 (6·9–14·5)	23·7 (8·7–41·5)	0·0 (0·0–0·1)	10·6 (6·9–14·5)	23·6 (8·8–41·3)
Solomon Islands	332 (265–398)	66·9 (55·6–79·7)	34·3 (28·4–42·2)	9·3 (5·9–13·0)	32·9 (17·2–54·9)	0·1 (0·0–0·1)	9·3 (5·9–13·0)	32·7 (17·4–54·3)
Tokelau	0.994 (0.826–1.2)	70·7 (58·6–86·2)	37·7 (28·6–45·5)	9·7 (6·4–13·3)	36·2 (16·9–52·8)	0·0 (0·0–0·0)	9·8 (6·5–13·4)	36·9 (17·4–53·4)
Tonga	64.7 (53.1–78.4)	74·0 (61·2–89·4)	28·4 (21·7–37·0)	10·3 (6·6–14·2)	26·9 (6·6–46·5)	0·0 (0·0–0·1)	10·3 (6·6–14·2)	26·7 (6·3–46·3)

Tuvalu	7.63 (6.45–9.19)	66·7 (56·5–80·2)	35·5 (28·6–45·5)	9·2 (6·1–12·9)	34·1 (17·6–55·7)	0·0 (0·0–0·1)	9·2 (6·1–12·9)	33·7 (17·2–55·3)
Vanuatu	140 (115–167)	59·5 (49·9–71·1)	25·2 (17·2–32·6)	8·3 (5·4–11·5)	25·4 (13·2–40·5)	0·0 (0·0–0·1)	8·4 (5·4–11·6)	25·4 (13·1–40·4)
**Southeast Asia**	**525 000 (446 000–638 000)**	**74·9 (64·2–90·2)**	**34·8 (31·1–39·0)**	**10·3 (6·8–13·9)**	**33·6 (27·8–40·2)**	**3·6 (2·6–4·1)**	**13·8 (10·1–17·3)**	**17·1 (7·2–24·7)**
Cambodia	10 700 (9 050–12 900)	73·6 (63·7–87·5)	53·8 (45·3–65·8)	10·0 (6·7–14·0)	52·4 (31·4–73·4)	6·2 (3·4–7·9)	16·3 (11·8–20·2)	19·9 (1·3–41·8)
Indonesia	121 000 (101 000–151 000)	45·7 (38·1–55·9)	26·1 (22·4–30·2)	6·4 (4·2–8·8)	24·3 (18·4–30·5)	2·6 (1·0–3·6)	9·0 (6·5–11·5)	21·3 (9·3–34·4)
Laos	4140 (3500–4960)	70·8 (61·1–83·6)	46·8 (39·2–55·9)	9·7 (6·4–13·2)	46·4 (28·3–69·1)	5·3 (2·8–7·5)	15·0 (10·9–19·9)	9·0 (-8·5–32·8)
Malaysia	15 900 (13 400–19 500)	50·4 (42·5–61·1)	35·0 (27·7–42·2)	6·9 (4·5–9·7)	32·5 (17·1–49·6)	1·0 (0·8–1·6)	8·0 (5·5–10·9)	15·0 (-1·3–31·3)
Maldives	485 (395–595)	104·5 (89·6–122·8)	38·4 (28·0–47·8)	14·2 (9·4–19·3)	38·2 (20·5–55·0)	4·1 (3·2–5·1)	18·4 (13·4–23·9)	1·5 (-20·3–22·8)
Mauritius	1630 (1390–1940)	94·8 (80·8–113·1)	50·5 (39·2–61·0)	12·6 (8·3–17·9)	46·4 (26·3–68·0)	6·1 (5·2–6·9)	18·7 (14·3–23·9)	108·8 (79·5–154·7)
Myanmar	40 400 (34 700–48 700)	75·4 (65·4–89·8)	53·4 (44·7–63·5)	10·2 (6·8–14·6)	49·8 (33·5–70·1)	4·7 (2·7–6·0)	14·9 (10·8–19·6)	14·7 (-1·3–34·1)
Philippines	86 000 (71 800–104 000)	89·5 (76·4–108·2)	-10·2 (-11·5–-9·0)	12·1 (8·1–16·4)	-9·9 (-12·8–-6·8)	6·0 (4·5–7·0)	18·1 (13·8–22·5)	-10·1 (-15·7–-3·2)
Seychelles	77.8 (65.3–93.8)	64·2 (53·8–76·9)	33·8 (25·5–42·8)	8·9 (5·7–12·5)	32·3 (13·6–51·7)	0·6 (0·5–0·8)	9·6 (6·3–13·1)	27·4 (10·4–43·7)
Sri Lanka	14 100 (11 700–17 300)	55·2 (45·8–67·7)	36·2 (28·8–42·5)	7·6 (4·8–10·4)	32·8 (13·1–51·1)	0·6 (0·5–0·8)	8·2 (5·4–11·1)	26·4 (10·6–42·4)
Thailand	101 000 (84 300–118 000)	100·6 (83·1–119·2)	49·6 (41·8–57·3)	13·7 (9·0–18·7)	49·8 (28·8–66·9)	3·9 (3·0–5·0)	17·6 (12·8–22·8)	14·4 (-5·0–33·7)
Timor-Leste	622 (521–748)	64·1 (54·7–76·4)	41·4 (34·9–50·1)	8·7 (5·9–11·8)	39·7 (23·5–62·9)	4·6 (2·4–6·2)	13·3 (9·7–17·5)	21·5 (4·1–41·8)
Viet Nam	128 000 (108 000–153 000)	120·5 (103·0–142·1)	63·5 (54·2–73·6)	16·3 (10·3–21·8)	61·7 (42·2–85·7)	4·1 (2·3–5·5)	20·4 (14·6–26·1)	39·7 (18·1–59·4)
**Sub-Saharan Africa**	**641 000 (538 000–765 000)**	**96·3 (83·8–113·2)**	**3·8 (2·0–5·4)**	**12·9 (8·7–17·4)**	**3·3 (1·0–6·1)**	**2·3 (1·9–4·7)**	**15·2 (10·9–19·5)**	**-5·8 (-10·8–-0·5)**
**Central sub-Saharan Africa**	**78 900 (66 300–93 100)**	**103·5 (89·9–118·3)**	**19·4 (15·3–23·4)**	**13·7 (9·5–18·4)**	**19·4 (10·9–33·5)**	**0·9 (0·3–9·5)**	**14·6 (10·0–23·2)**	**13·5 (0·6–26·8)**
Angola	18 200 (15 300–21 400)	109·6 (95·4–124·9)	28·9 (23·6–34·3)	14·6 (9·8–19·5)	27·7 (11·6–41·5)	0·9 (0·2–9·7)	15·5 (10·3–24·1)	20·9 (2·6–35·6)

Central African Republic	3080 (2630–3590)	96·4 (85·6–109·5)	3·3 (-3·0–8·8)	12·7 (8·6–17·2)	3·0 (-9·4–16·5)	1·2 (0·4–13·8)	13·9 (9·2–25·8)	-1·1 (-13·8–11·4)
Congo	5380 (4650–6210)	144·3 (128·7–159·8)	24·8 (17·6–32·0)	19·1 (13·3–26·1)	24·0 (9·4–35·2)	0·9 (0·3–10·4)	20·0 (13·7–29·3)	17·2 (-2·8–29·6)
Democratic Republic of the Congo	48 900 (40 900–57 700)	96·4 (83·4–110·8)	15·2 (9·7–22·0)	12·7 (8·7–17·0)	15·5 (4·5–37·0)	0·9 (0·3–9·1)	13·6 (9·2–21·9)	10·2 (-3·8–29·3)
Equatorial Guinea	1130 (942–1340)	153·8 (133·6–174·4)	76·9 (67·2–88·4)	20·2 (13·1–27·0)	77·1 (53·3–100·3)	0·7 (0·2–8·6)	20·9 (13·4–29·5)	59·6 (2·2–91·0)
Gabon	2220 (1900–2590)	164·9 (145·5–188·3)	41·9 (34·7–50·8)	21·8 (14·9–30·4)	41·2 (27·5–56·1)	0·8 (0·3–9·2)	22·6 (15·4–32·2)	33·5 (6·8–50·5)
**Eastern sub-Saharan Africa**	**197 000 (164 000–236 000)**	**85·5 (74·6–98·9)**	**11·7 (9·6–13·3)**	**11·5 (7·6–15·3)**	**11·7 (8·5–14·8)**	**0·5 (0·2–5·5)**	**12·0 (8·0–17·0)**	**7·6 (-0·6–12·4)**
Burundi	5090 (4280–6060)	77·6 (67·9–89·6)	7·6 (3·4–12·9)	10·6 (7·2–14·4)	8·5 (-3·5–26·9)	0·5 (0·1–5·3)	11·1 (7·5–15·8)	3·7 (-11·4–20·7)
Comoros	530 (453–629)	92·2 (80·0–107·1)	18·4 (12·9–24·8)	12·4 (8·4–16·8)	17·6 (2·9–38·8)	0·5 (0·2–5·7)	12·9 (8·7–19·2)	13·8 (-0·3–33·1)
Djibouti	777 (641–921)	89·5 (76·6–103·3)	24·3 (18·1–32·5)	11·9 (7·7–15·7)	22·9 (8·7–40·2)	0·5 (0·1–4·6)	12·4 (8·0–17·5)	19·2 (4·0–37·2)
Eritrea	3290 (2800–3880)	85·4 (76·3–98·2)	18·1 (13·0–24·3)	11·3 (7·2–15·5)	16·9 (3·3–34·0)	0·6 (0·2–6·1)	11·9 (7·5–17·5)	12·4 (-3·9–27·3)
Ethiopia	45 800 (37 800–55 700)	76·6 (66·7–90·2)	-6·3 (-9·5–-3·4)	10·3 (6·8–14·1)	-5·2 (-10·6–0·8)	0·4 (0·1–4·8)	10·8 (7·1–15·2)	-9·6 (-16·9–-2·2)
Kenya	27 600 (23 000–33 500)	88·2 (76·6–104·0)	10·3 (8·3–12·3)	11·8 (7·9–16·0)	10·2 (6·9–14·1)	0·5 (0·2–5·0)	12·3 (8·2–17·7)	8·5 (4·4–12·5)
Madagascar	11 800 (9780–14 100)	74·2 (63·3–85·3)	17·1 (11·3–23·4)	10·0 (6·5–13·1)	16·5 (1·7–32·2)	0·4 (0·1–3·8)	10·4 (6·7–14·3)	12·9 (-1·9–29·2)
Malawi	9420 (7780–11 200)	93·3 (80·3–107·3)	21·7 (16·8–27·4)	12·4 (8·2–16·4)	21·4 (5·9–37·1)	0·6 (0·2–7·3)	13·1 (8·4–20·3)	17·2 (1·1–31·9)
Mozambique	13 100 (10 900–15 600)	86·5 (74·9–100·8)	21·0 (15·5–27·0)	11·4 (7·5–15·8)	19·9 (3·5–36·5)	0·7 (0·2–6·7)	12·2 (7·8–17·9)	16·0 (-0·0–32·0)
Rwanda	8280 (6970–9760)	102·2 (88·4–117·1)	29·5 (23·0–37·3)	13·7 (9·1–18·7)	28·1 (13·2–48·4)	0·5 (0·2–5·4)	14·2 (9·3–19·7)	20·0 (-0·6–42·8)
Somalia	8080 (6760–9520)	80·5 (70·8–91·7)	2·3 (-2·3–7·5)	10·8 (6·9–14·5)	1·8 (-10·8–15·7)	1·0 (0·3–9·0)	11·7 (7·5–20·2)	-1·8 (-13·2–11·7)
South Sudan	4280 (3560–5080)	80·3 (69·6–93·1)	16·7 (10·9–22·4)	10·7 (7·2–14·5)	17·0 (2·3–35·5)	0·6 (0·2–5·7)	11·3 (7·5–16·6)	13·7 (-0·4–31·1)
Uganda	18 400 (15 400–21 800)	90·7 (79·1–104·1)	25·0 (18·1–32·0)	12·1 (8·1–16·6)	23·8 (9·5–39·3)	0·5 (0·2–4·9)	12·6 (8·4–17·5)	20·5 (5·9–36·0)

Tanzania	31 000 (25 700–36 400)	94·2 (81·2–108·3)	21·9 (16·0–28·4)	12·7 (8·2–16·6)	22·0 (9·4–38·3)	0·4 (0·1–4·6)	13·1 (8·5–18·4)	17·3 (4·0–34·2)
Zambia	9690 (7810–11 600)	94·8 (80·7–110·0)	27·1 (19·6–33·2)	12·7 (8·3–17·1)	26·5 (13·5–43·6)	0·5 (0·2–4·9)	13·2 (8·7–17·7)	20·8 (2·7–37·4)
**Southern sub-Saharan Africa**	**188 000 (163 000–215 000)**	**260·5 (227·9–296·1)**	**-10·0 (-12·2–-8·2)**	**34·6 (23·4–46·8)**	**-11·0 (-14·5–-7·4)**	**15·9 (13·8–18·2)**	**50·5 (39·0–61·9)**	**-18·8 (-24·5–-9·9)**
Botswana	3720 (3140–4340)	184·2 (160·4–210·6)	33·2 (27·1–40·8)	24·4 (16·4–32·7)	31·1 (17·4–45·5)	9·0 (2·5–12·5)	33·4 (24·8–42·6)	-0·0 (-20·1–19·6)
Eswatini	1640 (1400–1890)	198·4 (173·6–224·0)	13·0 (6·3–18·9)	26·2 (17·7–35·4)	10·8 (-2·1–25·4)	17·1 (4·4–31·0)	43·3 (29·8–59·6)	-1·0 (-16·0–22·8)
Lesotho	2810 (2380–3250)	167·2 (144·7–188·6)	19·1 (12·7–26·6)	22·0 (14·4–30·6)	16·7 (5·6–30·1)	16·5 (5·4–24·3)	38·5 (27·7–49·6)	21·3 (-0·7–52·2)
Namibia	2990 (2480–3490)	163·2 (137·2–187·6)	26·9 (21·2–33·6)	21·7 (13·7–29·4)	25·1 (10·7–41·6)	10·6 (3·2–16·3)	32·3 (22·9–42·4)	2·6 (-15·2–16·6)
South Africa	166 000 (144 000–189 000)	299·5 (262·3–340·7)	-13·6 (-16·0–-11·6)	39·8 (27·0–54·1)	-14·5 (-18·4–-11·0)	16·8 (14·5–19·7)	56·6 (43·0–69·9)	-23·2 (-28·6–-14·3)
Zimbabwe	10 800 (9030–12 600)	102·4 (87·5–118·1)	1·2 (-4·0–7·3)	13·8 (9·4–19·5)	0·9 (-12·2–17·0)	12·4 (6·4–17·3)	26·2 (19·2–33·5)	12·1 (-3·4–35·7)
**Western sub-Saharan Africa**	**177 000 (144 000–220 000)**	**61·3 (51·6–75·1)**	**25·9 (22·5–29·4)**	**8·4 (5·5–11·7)**	**24·7 (19·9–30·2)**	**0·1 (0·1–0·2)**	**8·5 (5·7–11·9)**	**24·3 (19·9–29·8)**
Benin	4800 (3890–5900)	63·7 (52·9–77·5)	25·3 (18·6–32·9)	8·6 (5·8–12·1)	23·1 (7·8–44·8)	0·1 (0·0–0·1)	8·7 (5·9–12·2)	22·0 (7·4–43·8)
Burkina Faso	8130 (6510–10 100)	59·3 (49·6–72·0)	22·6 (15·1–33·1)	8·1 (5·4–11·3)	21·8 (4·5–39·3)	0·1 (0·0–0·2)	8·2 (5·5–11·4)	19·8 (2·2–37·7)
Cabo Verde	369 (298–452)	71·6 (58·7–86·6)	34·7 (28·6–42·0)	9·7 (6·4–13·4)	31·8 (13·5–54·3)	0·0 (0·0–0·1)	9·7 (6·4–13·4)	30·6 (12·8–53·4)
Cameroon	13 600 (10 900–16 800)	71·9 (60·1–87·8)	20·1 (14·2–26·2)	9·7 (6·1–12·7)	19·7 (2·5–39·6)	0·1 (0·0–0·2)	9·8 (6·2–12·8)	18·3 (1·7–37·3)
Chad	4210 (3360–5240)	48·6 (40·5–58·8)	11·3 (6·0–18·0)	6·6 (4·4–8·8)	10·3 (-0·7–25·0)	0·1 (0·0–0·2)	6·7 (4·5–9·0)	9·5 (-1·4–24·1)
Côte d’Ivoire	11 100 (8820–13 700)	64·8 (54·5–78·2)	25·8 (18·4–32·5)	8·8 (5·9–12·5)	26·8 (10·2–45·4)	0·1 (0·0–0·1)	8·9 (5·9–12·6)	25·7 (9·4–43·9)
Gambia	899 (709–1110)	63·6 (52·2–78·1)	27·7 (19·9–36·9)	8·5 (5·7–11·5)	24·6 (9·3–44·6)	0·1 (0·0–0·2)	8·6 (5·8–11·6)	23·4 (8·4–42·9)
Ghana	15 800 (12 800–19 800)	67·2 (55·8–82·3)	42·9 (34·8–53·4)	9·1 (6·1–12·7)	40·2 (20·5–67·2)	0·7 (0·3–1·4)	9·9 (6·9–13·5)	49·2 (28·5–75·2)
Guinea	4340 (3590–5300)	56·1 (47·4–67·4)	16·9 (10·7–23·5)	7·6 (4·8–10·6)	14·5 (-2·0–30·6)	0·1 (0·0–0·1)	7·7 (4·9–10·7)	13·7 (-2·5–29·3)

Guinea-Bissau	669 (536–827)	57·3 (48·8–68·8)	19·4 (12·8–27·4)	7·8 (5·1–10·8)	17·8 (3·3–36·0)	0·1 (0·0–0·2)	7·9 (5·2–10·9)	16·3 (1·3–33·5)
Liberia	2190 (1750–2660)	66·1 (54·7–79·4)	26·3 (19·4–34·6)	8·8 (5·8–12·5)	24·1 (8·7–42·6)	0·1 (0·0–0·2)	8·9 (5·9–12·6)	22·8 (6·7–41·2)
Mali	6800 (5400–8380)	53·5 (44·2–65·2)	20·7 (15·1–28·3)	7·3 (4·9–10·4)	20·6 (5·4–40·2)	0·1 (0·0–0·2)	7·4 (4·9–10·6)	18·8 (4·6–36·7)
Mauritania	2160 (1760–2640)	78·4 (65·1–95·1)	40·2 (31·8–48·2)	10·6 (6·7–14·9)	37·5 (21·8–53·9)	0·1 (0·0–0·1)	10·7 (6·8–15·0)	35·5 (20·1–51·2)
Niger	6020 (4830–7510)	50·3 (42·0–61·2)	17·7 (11·8–25·5)	6·9 (4·5–9·7)	15·8 (0·2–34·0)	0·1 (0·0–0·2)	7·0 (4·6–9·8)	14·3 (-1·2–32·7)
Nigeria	83 100 (67 800–104 000)	60·6 (51·4–74·9)	25·9 (22·0–30·5)	8·3 (5·5–11·6)	24·7 (19·3–30·2)	0·1 (0·0–0·1)	8·4 (5·6–11·7)	23·7 (18·5–28·9)
São Tomé and Príncipe	110 (89–137)	73·0 (61·4–89·0)	30·5 (21·4–36·4)	9·9 (6·3–13·8)	28·6 (16·2–49·6)	0·1 (0·0–0·1)	10·0 (6·3–13·9)	27·7 (15·5–48·1)
Senegal	6540 (5260–8180)	64·3 (53·0–79·4)	24·1 (18·7–33·2)	8·6 (5·7–12·2)	22·4 (7·9–39·5)	0·1 (0·0–0·1)	8·7 (5·8–12·3)	21·2 (7·1–39·2)
Sierra Leone	3030 (2460–3770)	56·2 (46·9–67·1)	16·5 (10·3–23·6)	7·7 (5·0–11·0)	17·7 (2·9–37·3)	0·1 (0·0–0·1)	7·8 (5·1–11·1)	16·8 (2·3–35·5)
Togo	3400 (2740–4090)	62·2 (51·6–73·8)	23·2 (17·0–30·7)	8·5 (5·5–11·7)	22·8 (6·0–37·7)	0·1 (0·0–0·1)	8·6 (5·6–11·8)	21·9 (5·4–36·5)

YLD = Years lived with disability; DALY = Disability Adjusted Life Years

Capitalised = GBD Super-regions; Bold = GBD Regions

Note: Super-region and region numbers do not sum to the global prevalence due to rounding and modelling adjustments for nations with populations below 50,000

Supplemental Figure 4: Age-standardised disability-adjusted life-years (DALYs) for rheumatoid arthritis per 100,000 population by location, 2020

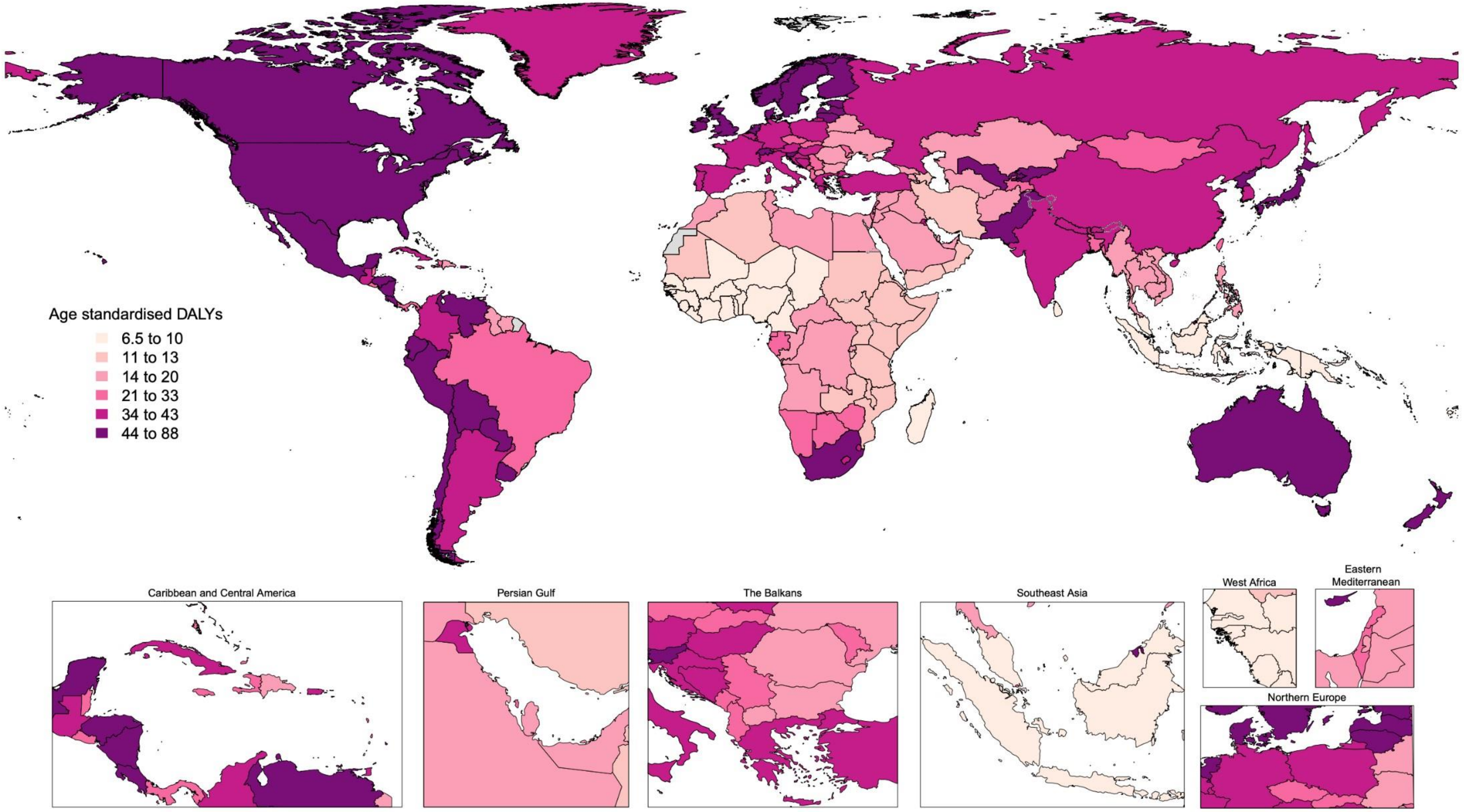

Supplemental Table 7: **Forecast of rheumatoid arthritis age-standardised prevalence and total cases globally and by region, both sexes, from 2020 to 2050**

	**Age-standardised prevalence (%)**			**Cases (millions)**		
**Region**	**2030**	**2040**	**2050**	**2030**	**2040**	**2050**
Global	0·212 (0·186–0·247)	0·220 (0·188–0·265)	0·230 (0·189–0·289)	21·9 (19·5–25·1)	26·7 (22·9–31·6)	31·7 (25·8–39·0)
Andean Latin America	0·427 (0·382–0·484)	0·435 (0·382–0·506)	0·445 (0·387–0·533)	0·324 (0·291–0·362)	0·388 (0·343–0·449)	0·453 (0·383–0·544)
Australasia	0·346 (0·295–0·412)	0·346 (0·286–0·442)	0·353 (0·272–0·481)	0·178 (0·151–0·209)	0·202 (0·164–0·253)	0·229 (0·172–0·304)
Caribbean	0·152 (0·125–0·188)	0·152 (0·115–0·207)	0·153 (0·105–0·222)	0·0931 (0·0778–0·113)	0·106 (0·0834–0·140)	0·117 (0·0849–0·166)
Central Asia	0·192 (0·152–0·238)	0·199 (0·138–0·276)	0·206 (0·128–0·312)	0·234 (0·192–0·282)	0·294 (0·214–0·394)	0·349 (0·236–0·508)
Central Europe	0·199 (0·161–0·251)	0·199 (0·135–0·285)	0·199 (0·113–0·319)	0·355 (0·280–0·435)	0·359 (0·252–0·497)	0·346 (0·205–0·546)
Central Latin America	0·372 (0·317–0·441)	0·386 (0·313–0·492)	0·396 (0·301–0·535)	1·20 (1·05–1·40)	1·50 (1·25–1·86)	1·77 (1·39–2·26)
Central sub-Saharan Africa	0·127 (0·112–0·148)	0·151 (0·131–0·180)	0·174 (0·145–0·211)	0·149 (0·126–0·169)	0·266 (0·219–0·308)	0·439 (0·351–0·538)
East Asia	0·236 (0·199–0·281)	0·241 (0·196–0·293)	0·250 (0·198–0·327)	5·62 (4·85–6·60)	6·18 (5·10–7·51)	6·40 (5·20–8·24)
Eastern Europe	0·180 (0·151–0·227)	0·181 (0·140–0·248)	0·182 (0·116–0·265)	0·524 (0·445–0·645)	0·517 (0·412–0·684)	0·508 (0·353–0·722)
Eastern sub-Saharan Africa	0·123 (0·107–0·144)	0·163 (0·136–0·198)	0·207 (0·164–0·269)	0·473 (0·398–0·540)	0·991 (0·806–1·22)	1·87 (1·46–2·43)
High income Asia Pacific	0·256 (0·214–0·307)	0·255 (0·211–0·322)	0·258 (0·189–0·343)	0·909 (0·786–1·06)	0·909 (0·753–1·11)	0·877 (0·670–1·13)
High income North America	0·305 (0·262–0·355)	0·305 (0·238–0·395)	0·302 (0·209–0·431)	1·88 (1·62–2·19)	2·03 (1·62–2·68)	2·13 (1·53–3·16)
North Africa and Middle East	0·116 (0·102–0·138)	0·117 (0·101–0·144)	0·119 (0·0963–0·156)	0·881 (0·772–1·03)	1·10 (0·939–1·33)	1·31 (1·05–1·70)
Oceania	0·0506 (0·0410–0·0658)	0·0504 (0·0385–0·0688)	0·0503 (0·0338–0·0766)	0·00714 (0·00577–0·00911)	0·00917 (0·00684–0·0125)	0·0116 (0·00764–0·0171)
South Asia	0·231 (0·206–0·269)	0·255 (0·227–0·299)	0·277 (0·243–0·324)	4·88 (4·40–5·54)	6·98 (6·26–7·89)	9·43 (8·17–10·8)

Southeast Asia	0·0758 (0·0622–0·0936)	0·0764 (0·0615–0·0977)	0·0774 (0·0574–0·105)	0·680 (0·569–0·826)	0·817 (0·658–1·02)	0·922 (0·698–1·23)
Southern Latin America	0·262 (0·226–0·307)	0·261 (0·203–0·334)	0·260 (0·190–0·348)	0·241 (0·208–0·278)	0·272 (0·214–0·346)	0·295 (0·212–0·406)
Southern sub-Saharan Africa	0·277 (0·247–0·319)	0·290 (0·247–0·354)	0·302 (0·240–0·387)	0·256 (0·225–0·294)	0·335 (0·286–0·400)	0·416 (0·332–0·503)
Tropical Latin America	0·173 (0·136–0·225)	0·176 (0·118–0·268)	0·175 (0·101–0·308)	0·527 (0·422–0·682)	0·604 (0·405–0·888)	0·646 (0·370–1·07)
Western Europe	0·276 (0·239–0·326)	0·276 (0·219–0·346)	0·279 (0·199–0·378)	2·13 (1·86–2·49)	2·23 (1·81–2·77)	2·29 (1·71–3·10)
Western sub-Saharan Africa	0·0639 (0·0531–0·0776)	0·0664 (0·0542–0·0808)	0·0693 (0·0577–0·0848)	0·264 (0·221–0·318)	0·392 (0·331–0·476)	0·568 (0·465–0·704)

Data input sources

Chaiamnuay P, Darmawan J, Muirden KD, Assawatanabodee P. Epidemiology of rheumatic disease in rural Thailand: a WHO-ILAR COPCORD study. Community Oriented Programme for the Control of Rheumatic Disease. J Rheumatol. 1998; 25(7): 1382-7.

Davatchi F, Jamshidi A-R, Banihashemi AT, Gholami J, Forouzanfar MH, Akhlaghi M, Barghamdi M, Noorolahzadeh E, Khabazi A-R, Salesi M, Salari A-H, Karimifar M, Essalat-Manesh K, Hajialiloo M, Soroosh M, Farzad F, Moussavi H-R, Samadi F, Ghaznavi K, Asgharifard H, Zangiabadi A-H, Shahram F, Nadji A, Akbarian M, Gharibdoost F. WHO-ILAR COPCORD Study (Stage 1, Urban Study) in Iran. J Rheumatol. 2008; 35(7): 1384.

Hanova P, Pavelka K, Dostal C, Holcatova I, Pikhart H. Epidemiology of rheumatoid arthritis, juvenile idiopathic arthritis and gout in two regions of the Czech Republic in a descriptive population-based survey in 2002-2003. Clin Exp Rheumatol. 2006; 24(5): 499-507.

Al-Awadhi AM, Olusi SO, Moussa M, Shehab D, Al-Zaid N, Al-Herz A, Al-Jarallah K. Musculoskeletal pain, disability and health-seeking behavior in adult Kuwaitis using a validated Arabic version of the WHO-ILAR COPCORD Core Questionnaire. Clin Exp Rheumatol. 2004; 22(2): 177-83.

Andrianakos A, Trontzas P, Christoyannis F, Dantis P, Voudouris C, Georgountzos A, Kaziolas G, Vafiadou E, Pantelidou K, Karamitsos D, Kontelis L, Krachtis P, Nikolia Z, Kaskani E, Tavaniotou E, Antoniades C, Karanikolas G, Kontoyanni A, ESORDIG Study. Prevalence of rheumatic diseases in Greece: a cross-sectional population based epidemiological study. The ESORDIG Study. J Rheumatol. 2003; 30(7): 1589-601.

Cardiel MH, Rojas-Serrano J. Community based study to estimate prevalence, burden of illness and help seeking behavior in rheumatic diseases in Mexico City. A COPCORD study. Clin Exp Rheumatol. 2002; 20(5): 617-24.

Haq SA, Darmawan J, Islam MN, Uddin MZ, Das BB, Rahman F, Chowdhury MAJ, Alam MN, Mahmud TAK, Chowdhury MR, Tahir M. Prevalence of rheumatic diseases and associated outcomes in rural and urban communities in Bangladesh: a COPCORD study. J Rheumatol. 2005; 32(2): 348-53.

Chou CT, Pei L, Chang DM, Lee CF, Schumacher HR, Liang MH. Prevalence of rheumatic diseases in Taiwan: a population study of urban, suburban, rural differences. J Rheumatol. 1994; 21(2): 302-6.

Dai S-M, Han X-H, Zhao D-B, Shi Y-Q, Liu Y, Meng J-M. Prevalence of rheumatic symptoms, rheumatoid arthritis, ankylosing spondylitis, and gout in Shanghai, China: a COPCORD study. J Rheumatol. 2003; 30(10): 2245-51.

Farooqi A, Gibson T. Prevalence of the major rheumatic disorders in the adult population of north Pakistan. Br J Rheumatol. 1998; 37(5): 491-5.

Reyes-Llerena GA, Guibert-Toledano M, Penedo-Coello A, Pérez-Rodríguez A, Baez-Dueñas RM, Charnicharo-Vidal R, Cardiel MH. Community-based study to estimate prevalence and burden of illness of rheumatic diseases in Cuba: a COPCORD study. J Clin Rheumatol. 2009; 15(2): 51-5.

Wigley R, Manahan L, Muirden KD, Caragay R, Pinfold B, Couchman KG, Valkenburg HA. Rheumatic disease in a Philippine village. II: a WHO-ILAR-APLAR COPCORD study, phases II and III. Rheumatol Int. 1991; 11(4-5): 157-61.

Minh Hoa TT, Darmawan J, Chen SL, Van Hung N, Thi Nhi C, Ngoc An T, Damarwan J, Shun Le C. Prevalence of the rheumatic diseases in urban Vietnam: a WHO-ILAR COPCORD study. J Rheumatol. 2003; 30(10): 2252-6.

Salaffi F, De Angelis R, Grassi W, MArche Pain Prevalence, INvestigation Group (MAPPING) study. Prevalence of musculoskeletal conditions in an Italian population sample: results of a regional community-based study. I. The MAPPING study. Clin Exp Rheumatol. 2005; 23(6): 819-28.

Bennett K, Cardiel MH, Ferraz MB, Riedemann P, Goldsmith CH, Tugwell P. Community screening for rheumatic disorder: cross cultural adaptation and screening characteristics of the COPCORD Core Questionnaire in Brazil, Chile, and Mexico. J Rheumatol. 1997; 24(1): 160-8.

Dans LF, Tankeh-Torres S, Amante CM, Penserga EG. The prevalence of rheumatic diseases in a Filipino urban population: a WHO-ILAR COPCORD Study. World Health Organization. International League of Associations for Rheumatology. Community Oriented Programme for the Control of the Rheumatic Diseases. J Rheumatol. 1997; 24(9): 1814-9.

Joshi VL, Chopra A. Is there an urban-rural divide? Population surveys of rheumatic musculoskeletal disorders in the Pune region of India using the COPCORD Bhigwan model. J Rheumatol. 2009; 36(3): 614-22.

Veerapen K, Wigley RD, Valkenburg H. Musculoskeletal pain in Malaysia: a COPCORD survey. J Rheumatol. 2007; 34(1): 207-13.

Watson DJ, Rhodes T, Guess HA. All-cause mortality and vascular events among patients with rheumatoid arthritis, osteoarthritis, or no arthritis in the UK General Practice Research Database. J Rheumatol. 2003; 30(6): 1196-202.

Eriksson JK, Neovius M, Ernestam S, Lindblad S, Simard JF, Askling J. Incidence of rheumatoid arthritis in Sweden: a nationwide population-based assessment of incidence, its determinants, and treatment penetration. Arthritis Care Res (Hoboken). 2013; 65(6): 870-8.

Malemba JJ, Mbuyi-Muamba JM, Mukaya J, Bossuyt X, Verschueren P, Westhovens R. The epidemiology of rheumatoid arthritis in Kinshasa, Democratic Republic of Congo--a population-based study. Rheumatology (Oxford). 2012; 51(9): 1644-7.

Sardu C, Cocco E, Mereu A, Massa R, Cuccu A, Marrosu MG, Contu P. Population based study of 12 autoimmune diseases in Sardinia, Italy: prevalence and comorbidity. PLoS One. 2012; 7(3): e32487.

Crowson CS, Matteson EL, Myasoedova E, Michet CJ, Ernste FC, Warrington KJ, Davis JM 3rd, Hunder GG, Therneau TM, Gabriel SE. The lifetime risk of adult-onset rheumatoid arthritis and other inflammatory autoimmune rheumatic diseases. Arthritis Rheum. 2011; 63(3): 633-9.

Neovius M, Simard JF, Askling J, ARTIS study group. Nationwide prevalence of rheumatoid arthritis and penetration of disease-modifying drugs in Sweden. Ann Rheum Dis. 2011; 70(4): 624-9.

Anagnostopoulos I, Zinzaras E, Alexiou I, Papathanasiou AA, Davas E, Koutroumpas A, Barouta G, Sakkas LI. The prevalence of rheumatic diseases in central Greece: a population survey. BMC Musculoskelet Disord. 2010; 98.

Li R, Sun J, Ren L-M, Wang H-Y, Liu W-H, Zhang X-W, Chen S, Mu R, He J, Zhao Y, Long L, Liu Y-Y, Liu X, Lu X-L, Li Y-H, Wang S-Y, Pan S-S, Li C, Wang H-Y, Li Z-G. Epidemiology of eight common rheumatic diseases in China: a large-scale cross-sectional survey in Beijing. Rheumatology (Oxford). 2012; 51(4): 721-9.

Duncan R, Francis RM, Collerton J, Davies K, Jagger C, Kingston A, Kirkwood T, Robinson L, Birrell F. Prevalence of arthritis and joint pain in the oldest old: findings from the Newcastle 85+ study. Age Ageing. 2011; 40(6): 752-5.

Carmona L, Ballina J, Gabriel R, Laffon A, EPISER Study Group. The burden of musculoskeletal diseases in the general population of Spain: results from a national survey. Ann Rheum Dis. 2001; 60(11): 1040-5.

Vandenbroucke JP, Hazevoet HM, Cats A. Survival and cause of death in rheumatoid arthritis: a 25-year prospective followup. J Rheumatol. 1984; 11(2): 158-61.

Allebeck P, Ahlbom A, Allander E. Increased mortality among persons with rheumatoid arthritis, but where RA does not appear on death certificate: eleven-year follow-up of an epidemiological study. Scand J Rheumatol. 1981; 10(4): 301-6.

Gonzalez A, Maradit Kremers H, Crowson CS, Nicola PJ, Davis JM 3rd, Therneau TM, Roger VL, Gabriel SE. The widening mortality gap between rheumatoid arthritis patients and the general population. Arthritis Rheum. 2007; 56(11): 3583-7.

Wolfe F, Mitchell DM, Sibley JT, Fries JF, Bloch DA, Williams CA, Spitz PW, Haga M, Kleinheksel SM, Cathey MA. The mortality of rheumatoid arthritis. Arthritis Rheum. 1994; 37(4): 481-94.

Gabriel SE, Crowson CS, O'Fallon WM. Mortality in rheumatoid arthritis: have we made an impact in 4 decades?. J Rheumatol. 1999; 26(12): 2529-33.

Uddin J, Kraus Fapha ASS, Garfield Kelly H. Survivorship and Death in Rheumatoid Arthritis. Arthritis Rheum. 1970; 13(2): 125-30.

Chehata JC, Hassell AB, Clarke SA, Mattey DL, Jones MA, Jones PW, Dawes PT. Mortality in rheumatoid arthritis: relationship to single and composite measures of disease activity. Rheumatology (Oxford). 2001; 40(4): 447-52.

Allebeck P. Increased mortality in rheumatoid arthritis: The use of a medical information system for assessment of death risks. Scand J Rheumatol. 1982; 11(2): 81-6.

Björnådal L, Baecklund E, Yin L, Granath F, Klareskog L, Ekbom A. Decreasing mortality in patients with rheumatoid arthritis: results from a large population based cohort in Sweden, 1964-95. J Rheumatol. 2002; 29(5): 906-12.

Carmona L, Descalzo MÁ, Perez-Pampin E, Ruiz-Montesinos D, Erra A, Cobo T, Gómez-Reino JJ. All-cause and cause-specific mortality in rheumatoid arthritis are not greater than expected when treated with tumour necrosis factor antagonists. Ann Rheum Dis. 2007; 66(7): 880-5.

Goodson N, Marks J, Lunt M, Symmons D. Cardiovascular admissions and mortality in an inception cohort of patients with rheumatoid arthritis with onset in the 1980s and 1990s. Ann Rheum Dis. 2005; 64(11): 1595-601.

Thomas E, Symmons DPM, Brewster DH, Black RJ, Macfarlane GJ. National study of cause-specific mortality in rheumatoid arthritis, juvenile chronic arthritis, and other rheumatic conditions: a 20 year followup study. J Rheumatol. 2003; 30(5): 958-65.

Young A, Koduri G, Batley M, Kulinskaya E, Gough A, Norton S, Dixey J. Mortality in rheumatoid arthritis. Increased in the early course of disease, in ischaemic heart disease and in pulmonary fibrosis. Rheumatology (Oxford). 2007; 46(2): 350-7.

Mutru O, Laakso M, Isomäki H, Koota K. Ten year mortality and causes of death in patients with rheumatoid arthritis. Br Med J (Clin Res Ed). 1985; 290(6484): 1797-9.

Silman AJ, Ollier W, Holligan S, Birrell F, Adebajo A, Asuzu MC, Thomson W, Pepper L. Absence of rheumatoid arthritis in a rural Nigerian population. J Rheumatol. 1993; 20(4): 618-22.

Englund M, Jöud A, Geborek P, Felson DT, Jacobsson LT, Petersson IF. Prevalence and incidence of rheumatoid arthritis in southern Sweden 2008 and their relation to prescribed biologics. Rheumatology (Oxford). 2010; 49(8): 1563-9.

Stojanović R, Vlajinac H, Palić-Obradović D, Janosević S, Adanja B. Prevalence of rheumatoid arthritis in Belgrade, Yugoslavia. Br J Rheumatol. 1998; 37(7): 729-32.

Cimmino MA, Parisi M, Moggiana G, Mela GS, Accardo S. Prevalence of rheumatoid arthritis in Italy: the Chiavari Study. Ann Rheum Dis. 1998; 57(5): 315-8.

Senna ER, De Barros ALP, Silva EO, Costa IF, Pereira LVB, Ciconelli RM, Ferraz MB. Prevalence of rheumatic diseases in Brazil: a study using the COPCORD approach. J Rheumatol. 2004; 31(3): 594-7.

Rasch EK, Hirsch R, Paulose-Ram R, Hochberg MC. Prevalence of rheumatoid arthritis in persons 60 years of age and older in the United States: effect of different methods of case classification. Arthritis Rheum. 2003; 48(4): 917-26.

Guillemin F, Saraux A, Guggenbuhl P, Roux CH, Fardellone P, Le Bihan E, Cantagrel A, Chary-Valckenaere I, Euller-Ziegler L, Flipo R-M, Juvin R, Behier J-M, Fautrel B, Masson C, Coste J. Prevalence of rheumatoid arthritis in France: 2001. Ann Rheum Dis. 2005; 64(10): 1427-30.

Shichikawa K, Inoue K, Hirota S, Maeda A, Ota H, Kimura M, Ushiyama T, Tsujimoto M. Changes in the incidence and prevalence of rheumatoid arthritis in Kamitonda, Wakayama, Japan, 1965-1996. Ann Rheum Dis. 1999; 58(12): 751-6.

Bjelle A, Allander E. Regional distribution of rheumatic complaints in Sweden. Scand J Rheumatol. 1981; 10(1): 9-15.

Gabriel SE, Crowson CS, O'Fallon WM. The epidemiology of rheumatoid arthritis in Rochester, Minnesota, 1955-1985. Arthritis Rheum. 1999; 42(3): 415-20.

Adomaviciute D, Pileckyte M, Baranauskaite A, Morvan J, Dadoniene J, Guillemin F. Prevalence survey of rheumatoid arthritis and spondyloarthropathy in Lithuania. Scand J Rheumatol. 2008; 37(2): 113-9.

Han C, Zhao N, Gaslightwala A, Bala M. An epidemiological and healthcare utilisation study of rheumatoid arthritis in an adult population in the US. J Med Econ. 2007; 10(4): 489-99.

Symmons D, Turner G, Webb R, Asten P, Barrett E, Lunt M, Scott D, Silman A. The prevalence of rheumatoid arthritis in the United Kingdom: new estimates for a new century. Rheumatology (Oxford). 2002; 41(7): 793-800.

Damjanović V, Vasilj I, Vlak T, Zelenika D. Prevalence and risk factors of the rheumatoid arthritis in Herzegovina region in 2003-2005. Coll Antropol. 2009; 73-7.

Kiss CG, Lövei C, Sütö G, Varjú C, Nagy Z, Füzesi Z, Illés T, Czirják L. Prevalence of rheumatoid arthritis in the South-Transdanubian region of Hungary based on a representative survey of 10,000 inhabitants. J Rheumatol. 2005; 32(9): 1688-90.

Zeng QY, Darmawan J, Xiao ZY, Chen SB, Chen R, Lin K, Wigley R, Chen SL, Zhang NZ. Risk factors associated with rheumatic complaints: a WHO-ILAR COPCORD study in Shantou, Southeast China. J Rheumatol. 2005; 32(5): 920-7.

Rodriguez-Amado J, Peláez-Ballestas I, Sanin LH, Esquivel-Valerio JA, Burgos-Vargas R, Pérez-Barbosa L, Riega-Torres J, Garza-Elizondo MA. Epidemiology of rheumatic diseases. A community-based study in urban and rural populations in the state of Nuevo Leon, Mexico. J Rheumatol Suppl. 2011; 9-14.

Peláez-Ballestas I, Sanin LH, Moreno-Montoya J, Alvarez-Nemegyei J, Burgos-Vargas R, Garza-Elizondo M, Rodríguez-Amado J, Goycochea-Robles M-V, Madariaga M, Zamudio J, Santana N, Cardiel MH, Grupo de Estudio Epidemiológico de Enfermedades Músculo Articulares (GEEMA). Epidemiology of the rheumatic diseases in Mexico. A study of 5 regions based on the COPCORD methodology. J Rheumatol Suppl. 2011; 86: 3-8.

Kaçar C, Gilgil E, Tuncer T, Bütün B, Urhan S, Arikan V, Dündar U, Oksüz MC, Sünbüloğlu G, Yildirim C, Tekeoğlu I, Yücel G. Prevalence of rheumatoid arthritis in Antalya, Turkey. Clin Rheumatol. 2005; 24(3): 212-4.

Akar S, Birlik M, Gurler O, Sari I, Onen F, Manisali M, Tirpan K, Demir T, Meral M, Akkoc N. The prevalence of rheumatoid arthritis in an urban population of Izmir-Turkey. Clin Exp Rheumatol. 2004; 22(4): 416-20.

Wigley RD, Zhang NZ, Zeng QY, Shi CS, Hu DW, Couchman K, Duff IF, Bennett PH. Rheumatic diseases in China: ILAR-China study comparing the prevalence of rheumatic symptoms in northern and southern rural populations. J Rheumatol. 1994; 21(8): 1484-90.

Darmawan J, Muirden KD, Valkenburg HA, Wigley RD. The epidemiology of rheumatoid arthritis in Indonesia. Br J Rheumatol. 1993; 32(7): 537-40.

Jacobsson L, Lindgärde F, Manthorpe R. The commonest rheumatic complaints of over six weeks' duration in a twelve-month period in a defined Swedish population. Prevalences and relationships. Scand J Rheumatol. 1989; 18(6): 353-60.

Lau E, Symmons D, Bankhead C, MacGregor A, Donnan S, Silman A. Low prevalence of rheumatoid arthritis in the urbanized Chinese of Hong Kong. J Rheumatol. 1993; 20(7): 1133-7.

Haq SA, Darmawan J, Islam MN, Ahmed M, Banik SK, Fazlur Rahman AKM, Alam MN, Tahir M, Rasker JJ. Incidence of musculoskeletal pain and rheumatic disorders in a Bangladeshi rural community: a WHO-APLAR-COPCORD study. Int J Rheum Dis. 2008; 11(3): 216-23.

Dugowson CE, Koepsell TD, Voigt LF, Bley L, Nelson JL, Daling JR. Rheumatoid arthritis in women. Incidence rates in Group Health Cooperative, Seattle, Washington, 1987-1989. Arthritis Rheum. 1991; 34(12): 1502-7.

Chan KW, Felson DT, Yood RA, Walker AM. Incidence of rheumatoid arthritis in central Massachusetts. Arthritis Rheum. 1993; 36(12): 1691-6.

Söderlin MK, Börjesson O, Kautiainen H, Skogh T, Leirisalo-Repo M. Annual incidence of inflammatory joint diseases in a population based study in southern Sweden. Ann Rheum Dis. 2002; 61(10): 911-5.

Rodríguez LAG, Tolosa LB, Ruigómez A, Johansson S, Wallander M-A. Rheumatoid arthritis in UK primary care: incidence and prior morbidity. Scand J Rheumatol. 2009; 38(3): 173-7.

Symmons DP, Barrett EM, Bankhead CR, Scott DG, Silman AJ. The incidence of rheumatoid arthritis in the United Kingdom: results from the Norfolk Arthritis Register. Br J Rheumatol. 1994; 33(8): 735-9.

Pedersen JK, Svendsen AJ, Hørslev-Petersen K. Incidence of Rheumatoid Arthritis in the Southern part of Denmark from 1995 to 2001. Open Rheumatol J. 2007; 18-23.

Myasoedova E, Crowson CS, Kremers HM, Therneau TM, Gabriel SE. Is the incidence of rheumatoid arthritis rising?: results from Olmsted County, Minnesota, 1955-2007. Arthritis Rheum. 2010; 62(6): 1576-82.

Wiles N, Symmons DP, Harrison B, Barrett E, Barrett JH, Scott DG, Silman AJ. Estimating the incidence of rheumatoid arthritis: trying to hit a moving target?. Arthritis Rheum. 1999; 42(7): 1339-46.

Odegård S, Kvien TK, Uhlig T. Incidence of clinically important 10-year health status and disease activity levels in population-based cohorts with rheumatoid arthritis. J Rheumatol. 2008; 35(1): 54-60.

Savolainen E, Kaipiainen-Seppänen O, Kröger L, Luosujärvi R. Total incidence and distribution of inflammatory joint diseases in a defined population: results from the Kuopio 2000 arthritis survey. J Rheumatol. 2003; 30(11): 2460-8.

Linos A, Worthington JW, O'fallon M, Kurland LT. The Epidemiology of Rheumatoid Arthritis in Rochester Minnesota: A Study of Incidence, Prevalence, and Mortality. Am J Epidemiol. 1980; 111(1): 87-98.

Alvarez-Nemegyei J, Pelaez-Ballestas I, Sanin LH, Cardiel MH, Ramirez-Angulo A, Goycochea-Robles M-V. Prevalence of musculoskeletal pain and rheumatic diseases in the southeastern region of Mexico. A COPCORD-based community survey. J Rheumatol. 2011; 86(Suppl): 21-5.

Cakır N, Pamuk ÖN, Derviş E, Imeryüz N, Uslu H, Benian Ö, Elelçi E, Erdem G, Sarvan FO, Senocak M. The prevalences of some rheumatic diseases in western Turkey: Havsa study. Rheumatol Int. 2012; 32(4): 895-908.

Chaaya M, Slim ZN, Habib RR, Arayssi T, Dana R, Hamdan O, Assi M, Issa Z, Uthman I. High burden of rheumatic diseases in Lebanon: a COPCORD study. Int J Rheum Dis. 2012; 15(2): 136-43.

American University of Beirut. Lebanon Community Oriented Programme for Control of Rheumatic Diseases Survey 2008.

Hameed K, Gibson T, Kadir M, Sultana S, Fatima Z, Syed A. The prevalence of rheumatoid arthritis in affluent and poor urban communities of Pakistan. Rheumatology (Oxford). 1995; 34(3): 252-6.

Riise T, Jacobsen BK, Gran J. Incidence and prevalence of rheumatoid arthritis in the county of Troms, northern Norway. J Rheumatol. 2000; 27(6): 1386-9.

Slimani S, Ladjouze-Rezig A. Prevalence of rheumatoid arthritis in an urban population of Algeria: a prospective study. Rheumatology (Oxford). 2014; 53(3): 571-3.

Widdifield J, Paterson JM, Bernatsky S, Tu K, Tomlinson G, Kuriya B, Thorne JC, Bombardier C. The epidemiology of rheumatoid arthritis (RA) in Ontario, Canada. Arthritis Rheum. 2014; 66(4): 786-93.

Zlatković-Švenda MI, Stojanović RM, B Šipetić-Grujičić S, Guillemin F. Prevalence of rheumatoid arthritis in Serbia. Rheumatol Int. 2014; 34(5): 649-58.

Yu K-H, See L-C, Kuo C-F, Chou I-J, Chou M-J. Prevalence and incidence in patients with autoimmune rheumatic diseases: a nationwide population-based study in Taiwan. Arthritis Care Res (Hoboken). 2013; 65(2): 244-50.

Book C, Saxne T, Jacobsson LTH. Prediction of mortality in rheumatoid arthritis based on disease activity markers. J Rheumatol. 2005; 32(3): 430-4.

Granados Y, Cedeno L, Rosillo C, Berbin S, Azocar M, Molina ME, Lara O, Sanchez G, Pelaez-Ballestas I. Prevalence of musculoskeletal disorders and rheumatic diseases in an urban community in Monagas State, Venezuela: a COPCORD study. Clin Rheumatol. 2015; 34(5): 871-7.

Indian Council of Medical Research (ICMR), Ministry of Health and Family Welfare (India), Vardhman Mahavir Medical College and Safdarjung Hospital (India). India Survey on Musculoskeletal Conditions 2007-2010.

Guevara-Pacheco S, Feicán-Alvarado A, Sanín LH, Vintimilla-Ugalde J, Vintimilla-Moscoso F, Delgado-Pauta J, Lliguisaca-Segarra A, Dután-Erráez H, Guevara-Mosquera D, Ochoa-Robles V, Cardiel MH, Peláez-Ballestas I. Prevalence of musculoskeletal disorders and rheumatic diseases in Cuenca, Ecuador: a WHO-ILAR COPCORD study. Rheumatol Int. 2016; 36(9): 1195-1204.

Courage UU, Stephen DP, Lucius IC, Ani C, Oche AO, Emmanuel AI, Olufemi AO. Prevalence of musculoskeletal diseases in a semi-urban Nigerian community: results of a cross-sectional survey using COPCORD methodology. Clin Rheumatol. 2017; 36(11): 2509-2516.

Paul BJ, Rahim AA, Bina T, Thekkekara RJ. Prevalence and factors related to rheumatic musculoskeletal disorders in rural south India: WHO-ILAR-COPCORD-BJD India Calicut study. Int J Rheum Dis. 2013; 16(4): 392-397.

Branco JC, Rodrigues AM, Gouveia N, Eusébio M, Ramiro S, Machado PM, da Costa LP, Mourão AF, Silva I, Laires P, Sepriano A, Araújo F, Gonçalves S, Coelho PS, Tavares V, Cerol J, Mendes JM, Carmona L, Canhão H, EpiReumaPt study group. Prevalence of rheumatic and musculoskeletal diseases and their impact on health-related quality of life, physical function and mental health in Portugal: results from EpiReumaPt- a national health survey. RMD Open. 2016; 2(1): e000166.

Zeng S-Y, Gong Y, Zhang Y-P, Chen S-B, Chen J-Y, Lin C-Q, Peng J-H, Hou Z-D, Zhong J-Q, Liang H-J, Huang G-H, Wang D-M, Lai H-Y, Li L-P, Zeng QY. Changes in the Prevalence of Rheumatic Diseases in Shantou, China, in the Past Three Decades: A COPCORD Study. PLoS One. 2015; 10(9): e0138492.

Abhishek A, Doherty M, Kuo C-F, Mallen CD, Zhang W, Grainge MJ. Rheumatoid arthritis is getting less frequent-results of a nationwide population-based cohort study. Rheumatology (Oxford). 2017; 56(5): 736–44.

Sun J, Liu D, Liu Z, Li W, Li G, Zhang J. [Epidemiology of rheumatoid arthritis in middle-aged and elderly population in Luohe City, Henan Province]. Chin Med J (Taipei). 2013; 93(41): 3309–11.

Widdifield J, Paterson JM, Bernatsky S, Tu K, Tomlinson G, Kuriya B, Thorne JC, Bombardier C. The Epidemiology of Rheumatoid Arthritis in Ontario, Canada. Arthritis Rheumatol. 2014; 66(4): 786–93.

Hense S, Ramos AL, Callhoff J, Albrecht K, Zink A, Hoffmann F. Prävalenz der rheumatoiden Arthritis in Deutschland auf Basis von Kassendaten. Z Rheumatol. 2016; 75(8): 819–27.

Crane MM, Juneja M, Allen J, Kurrasch RH, Chu ME, Quattrocchi E, Manson SC, Chang DJ. Epidemiology and Treatment of New-Onset and Established Rheumatoid Arthritis in an Insured US Population. Arthritis Care Res (Hoboken). 2015; 67(12): 1646–55.

Fina-Aviles F, Medina-Peralta M, Mendez-Boo L, Hermosilla E, Elorza JM, Garcia-Gil M, Ramos R, Bolibar B, Javaid MK, Edwards CJ, Cooper C, Arden NK, Prieto-Alhambra D. The descriptive epidemiology of rheumatoid arthritis in Catalonia: a retrospective study using routinely collected data. Clin Rheumatol. 2016; 35(3): 751–7.

Rossini M, Rossi E, Bernardi D, Viapiana O, Gatti D, Idolazzi L, Caimmi C, Derosa M, Adami S. Prevalence and incidence of rheumatoid arthritis in Italy. Rheumatol Int. 2014; 34(5): 659–64.

Sung Y-K, Cho S-K, Choi C-B, Bae S-C. Prevalence and incidence of rheumatoid arthritis in South Korea. Rheumatol Int. 2013; 33(6): 1525–32.

Otsa K, Tammaru M, Vorobjov S, Esko M, Pärsik E, Lang K. The prevalence of rheumatoid arthritis in Estonia: an estimate based on rheumatology patients’ database. Rheumatol Int. 2013; 33(4): 955–8.

Vega-Hinojosa O, Cardiel MH, Ochoa-Miranda P. Prevalence of musculoskeletal manifestations and related disabilities in a Peruvian urban population living at high altitude. COPCORD Study. Stage I. Reumatol Clin. 2018; 14(5): 278-284.

Kumar P, Alok R, Das SK, Srivastava R, Agarwal GG. Distribution of rheumatological diseases in rural and urban areas: An adapted COPCORD Stage I Phase III survey of Lucknow district in north India. Int J Rheum Dis. 2018; 21(11): 1894-1899.

Institute for Health Metrics and Evaluation (IHME). IHME Modeled Excess Mortality Rate (EMR).

Jeong HS, Hong SJ, Choi SJ, Kim JH, Song GG, Jung JH. Effects of oral contraceptives on rheumatoid arthritis in Korean menopausal women: A nationwide cross-sectional study. Maturitas. 2018; 112: 24-28.

Klinaki E, Katsoulis M, La Vecchia C, Trichopoulou A. Rheumatoid arthritis and cancer risk results from the Greek European prospective investigation into cancer and nutrition cohort. Eur J Cancer Prev. 2018; 27(5): 502-506.

Ministry of Health and Welfare (Taiwan). Taiwan National Health Insurance Claims Data 2016.

Authors' contributions

Providing data or critical feedback on data sources

Mitra Abbasifard, Ame Mehadi Abdurehman, Aidin Abedi, Hassan Abidi, Richard Gyan Aboagye, Hassan Abolhassani, Ahmed Abu-Zaid, Miracle Ayomikun Adesina, Qorinah Estiningtyas Sakilah Adnani, Muhammad Sohail Afzal, Ayman Ahmed, Janardhana P Aithala, Meisam Akhlaghdoust, Nelson J Alvis-Zakzuk, Hubert Amu, Jalal Arabloo, Judie Arulappan, Tahira Ashraf, Seyyed Shamsadin Athari, Alaa Badawi, Atif Amin Baig, Maciej Banach, Palash Chandra Banik, Amadou Barrow, Alehegn Bekele, Akshaya Srikanth Bhagavathula, Ajay Nagesh Bhat, Vijayalakshmi S Bhojaraja, Belay Boda Abule Bodicha, Peter M Brooks, Carlos A Castañeda-Orjuela, Periklis Charalampous, Vijay Kumar Chattu, Marita Cross, Natália Cruz-Martins, Garland T Culbreth, Xiaochen Dai, Fikadu Nugusu Dessalegn, Meghnath Dhimal, Paul Narh Doku, Karsten E Dreinhoefer, Michael Ekholuenetale, Takeshi Fukumoto, Balasankar Ganesan, Ahmad Ghashghaee, Vivek Kumar Gupta, Lydia M Haile, Rabih Halwani, Shafiul Haque, Netanja I Harlianto, Ahmed I Hasaballah, Simon I Hay, Nobuyuki Horita, Amir Human Hoveidaei, Salman Hussain, Sheikh Mohammed Shariful Islam, Nahlah Elkudssiah Ismail, Mihajlo Jakovljevic, Nitin Joseph, Vidya Kadashetti, Himal Kandel, Tesfaye K Kanko, Ibraheem M Karaye, Himanshu Khajuria, Moien AB Khan, Jagdish Khubchandani, Yun Jin Kim, Adnan Kisa, Kewal Krishan, Ambily Kuttikkattu, Stephen S Lim, Preetam Bhalchandra Mahajan, Mohammad Ali Mansournia, Lyn M March, Mohamed Kamal Mesregah, Erkin M Mirrakhimov, Awoke Misganaw, Shafiu Mohammed, Ali H Mokdad, Efrén Murillo-Zamora, Christopher J L Murray, Ghulam Mustafa, Mohsen Naghavi, Zuhair S Natto, Biswa Prakash Nayak, Cuong Tat Nguyen, Robina Khan Niazi, Ogochukwu Janet Nzoputam, Osaretin Christabel Okonji, Kanyin Liane Ong, Mayowa O Owolabi, Kevin Pacheco-Barrios, Jagadish Rao Padubidri, Jay Patel, Aslam Ramjan Pathan, Shrikant Pawar, Paolo Pedersini, Arokiasamy Perianayagam, Zubair Ahmed Ratan, Salman Rawaf, Elrashdy Moustafa Mohamed Redwan, Andre M N Renzaho, Aly M A Saad, Basema Saddik, Umar Saeed, Azam Safary, Allen Seylani, Masood Ali Shaikh, Adithi Shetty, Jeevan K Shetty, Parnian Shobeiri, Ambrish Singh, Jasvinder A Singh, Anna Aleksandrovna Skryabina, Yonatan Solomon, Mohammad Sadegh Soltani-Zangbar, Jaimie D Steinmetz, Mohammad Tabish, Ker-Kan Tan, Marcos Roberto Tovani-Palone, Sahel Valadan Tahbaz, Pascual R Valdez, Theo Vos, Dereje Y Yada, Sisay Shewasinad Yehualashet, Naohiro Yonemoto, Iman Zare, Zhi-Jiang Zhang, and Mohammad Zoladl.

Developing methods or computational machinery

Qorinah Estiningtyas Sakilah Adnani, Astawus Alemayehu, Hubert Amu, Aleksandr Y Aravkin, Akshaya Srikanth Bhagavathula, Kaleb Coberly, Garland T Culbreth, Xiaochen Dai, Fikadu Nugusu Dessalegn, Lydia M Haile, Simon I Hay, Adnan Kisa, Ali H Mokdad, Getaneh Baye Mulu, Christopher J L Murray, Mohsen Naghavi, Kanyin Liane Ong, Umar Saeed, Stein Emil Vollset, and Theo Vos.

Providing critical feedback on methods or results

Amirali Aali, Mitra Abbasifard, Ame Mehadi Abdurehman, Aidin Abedi, Hassan Abidi, Richard Gyan Aboagye, Hassan Abolhassani, Eman Abu-Gharbieh, Ahmed Abu-Zaid, Kidist Adamu, Isaac Yeboah Addo, Qorinah Estiningtyas Sakilah Adnani, Muhammad Sohail Afzal, Ayman Ahmed, Janardhana P Aithala, Meisam Akhlaghdoust, Astawus Alemayehu, Saba Alvand, Nelson J Alvis-Zakzuk, Hubert Amu, Jalal Arabloo, Judie Arulappan, Tahira Ashraf, Seyyed Shamsadin Athari, Sina Azadnajafabad, Alaa Badawi, Nayereh Baghcheghi, Atif Amin Baig, Asaminew Birhanu Balta, Maciej Banach, Palash Chandra Banik, Amadou Barrow, Lindsay M Bearne, Alehegn Bekele, Isabela M Bensenor, Alemshet Yirga Berhie, Akshaya Srikanth Bhagavathula, Pankaj Bhardwaj, Ajay Nagesh Bhat, Vijayalakshmi S Bhojaraja, Rachel J Black, Belay Boda Abule Bodicha, João Silva Botelho, Andrew M Briggs, Peter M Brooks, Rachelle Buchbinder, Carlos A Castañeda-Orjuela, Periklis Charalampous, Vijay Kumar Chattu, Marita Cross, Natália Cruz-Martins, Garland T Culbreth, Omid Dadras, Xiaochen Dai, Katie de Luca, Fikadu Nugusu Dessalegn, Gashaw Dessie, Meghnath Dhimal, Lankamo Ena Digesa, Mengistie Diress, Paul Narh Doku, Hisham Atan Edinur, Michael Ekholuenetale, Muhammed Elhadi, Yasser Mohamed El-Sherbiny, Farshid Etaee, Rana Ezzeddini, Shahriar Faghani, Irina Filip, Florian Fischer, Takeshi Fukumoto, Balasankar Ganesan, Mathewos Alemu Gebremichael, Motuma Erena Getachew, Ahmad Ghashghaee, Tiffany K Gill, Bhawna Gupta, Sapna Gupta, Vivek Kumar Gupta, Lydia M Haile, Rabih Halwani, Md Abdul Hannan, Shafiul Haque, Netanja I Harlianto, Mehdi Harorani, Ahmed I Hasaballah, Mohammed Bheser Hassen, Simon I Hay, Khezar Hayat, Golnaz Heidari, Kamal Hezam, Yuta Hiraike, Nobuyuki Horita, Amir Human Hoveidaei, Alexander Kevin Hsiao, Evelyn Hsieh, Salman Hussain, Irena M Ilic, Nahlah Elkudssiah Ismail, Masao Iwagami, Mihajlo Jakovljevic, Chinmay T Jani, Jayakumar Jeganathan, Nitin Joseph, Vidya Kadashetti, Himal Kandel, Tesfaye K Kanko, Ibraheem M Karaye, Himanshu Khajuria, Md Jobair Khan, Moien AB Khan, Javad Khanali, Moawiah Mohammad Khatatbeh, Jagdish Khubchandani, Yun Jin Kim, Adnan Kisa, Ali-Asghar Kolahi, Farzad Kompani, Hamid Reza Koohestani, Jacek A Kopec, Ai Koyanagi, Kewal Krishan, Mohammed Kuddus, Narinder Kumar, Ambily Kuttikkattu, Bagher Larijani, Stephen S Lim, Vanessa Sintra Machado, Preetam Bhalchandra Mahajan, Azeem Majeed, Elaheh Malakan Rad, Ahmad Azam Malik, Mohammad Ali Mansournia, Lyn M March, Elezebeth Mathews, José João Mendes, Alexios-Fotios A Mentis, Mohamed Kamal Mesregah, Tomislav Mestrovic, Seyed Peyman Mirghaderi, Erkin M Mirrakhimov, Ashraf Mohamadkhani, Shafiu Mohammed, Ali H Mokdad, Md Moniruzzaman, Ahmed Al Montasir, Getaneh Baye Mulu, Efrén Murillo-Zamora, Christopher J L Murray, Mohsen Naghavi, Tapas Sadasivan Nair, Atta Abbas Naqvi, Zuhair S Natto, Biswa Prakash Nayak, Subas Neupane, Cuong Tat Nguyen, Robina Khan Niazi, Ogochukwu Janet Nzoputam, Hassan Okati-Aliabad, Osaretin Christabel Okonji, Isaac Iyinoluwa Olufadewa, Kanyin Liane Ong, Mayowa O Owolabi, Kevin Pacheco-Barrios, Jagadish Rao Padubidri, Jay Patel, Aslam Ramjan Pathan, Shrikant Pawar, Paolo Pedersini, Arokiasamy Perianayagam, Ionela-Roxana Petcu,

Ibrahim Qattea, Amir Radfar, Alireza Rafiei, Vahid Rahmanian, Vahid Rashedi, Mohammad-Mahdi Rashidi, Zubair Ahmed Ratan, Salman Rawaf, Mohammad Sadegh Razeghinia, Elrashdy Moustafa Mohamed Redwan, Andre M N Renzaho, Nazila Rezaei, Nima Rezaei, Abanoub Riad, Aly M A Saad, Basema Saddik, Umar Saeed, Azam Safary, Maryam Sahebazzamani, Harihar Sahoo, Amir Salek Farrokhi, Muhammad Arif Nadeem Saqib, Saeed Shahabi, Masood Ali Shaikh, Bereket Beyene Shashamo, Jeevan K Shetty, Mika Shigematsu, Parnian Shobeiri, Migbar Mekonnen Sibhat, Ehsan Sinaei, Ambrish Singh, Jasvinder A Singh, Paramdeep Singh, Surjit Singh, Md Shahjahan Siraj, Anna Aleksandrovna Skryabina, Yonatan Solomon, Mohammad Sadegh Soltani-Zangbar, Jaimie D Steinmetz, Mohammad Tabish, Ker-Kan Tan, Samar Tharwat, Marcos Roberto Tovani-Palone, Biruk Shalmeno Tusa, Sahel Valadan Tahbaz, Pascual R Valdez, Rohollah Valizadeh, Siavash Vaziri, Stein Emil Vollset, Theo Vos, Ai-Min Wu, Dereje Y Yada, Naohiro Yonemoto, Yuyi You, Ismaeel Yunusa, Moein Zangiabadian, Armin Zarrintan, Zhi-Jiang Zhang, Chenwen Zhong, and Mohammad Zoladl.

Drafting the work or revising it critically for important intellectual content

Amirali Aali, Mohsen Abbasi-Kangevari, Ame Mehadi Abdurehman, Aidin Abedi, Hassan Abidi, Hassan Abolhassani, Eman Abu-Gharbieh, Ahmed Abu-Zaid, Isaac Yeboah Addo, Qorinah Estiningtyas Sakilah Adnani, Muhammad Sohail Afzal, Ayman Ahmed, Janardhana P Aithala, Meisam Akhlaghdoust, Saba Alvand, Nelson J Alvis-Zakzuk, Hubert Amu, Benny Antony, Jalal Arabloo, Judie Arulappan, Seyyed Shamsadin Athari, Sina Azadnajafabad, Alaa Badawi, Atif Amin Baig, Asaminew Birhanu Balta, Maciej Banach, Palash Chandra Banik, Amadou Barrow, Azadeh Bashiri, Lindsay M Bearne, Alehegn Bekele, Isabela M Bensenor, Neil Betteridge, Akshaya Srikanth Bhagavathula, Ajay Nagesh Bhat, Vijayalakshmi S Bhojaraja, Saeid Bitaraf, Rachel J Black, Belay Boda Abule Bodicha, João Silva Botelho, Peter M Brooks, Rachelle Buchbinder, Carlos A Castañeda-Orjuela, Vijay Kumar Chattu, Marita Cross, Natália Cruz-Martins, Garland T Culbreth, Katie de Luca, Fikadu Nugusu Dessalegn, Meghnath Dhimal, Paul Narh Doku, Karsten E Dreinhoefer, Muhammed Elhadi, Yasser Mohamed El-Sherbiny, Farshid Etaee, Rana Ezzeddini, Shahriar Faghani, Irina Filip, Florian Fischer, Takeshi Fukumoto, Balasankar Ganesan, Mathewos Alemu Gebremichael, Urge Gerema, Motuma Erena Getachew, Tiffany K Gill, Bhawna Gupta, Sapna Gupta, Veer Bala Gupta, Vivek Kumar Gupta, Lydia M Haile, Rabih Halwani, Md Abdul Hannan, Shafiul Haque, Netanja I Harlianto, Ahmed I Hasaballah, Simon I Hay, Khezar Hayat, Golnaz Heidari, Kamal Hezam, Catherine L Hill, Yuta Hiraike, Nobuyuki Horita, Amir Human Hoveidaei, Alexander Kevin Hsiao, Evelyn Hsieh, Salman Hussain, Ivo Iavicoli, Irena M Ilic, Sheikh Mohammed Shariful Islam, Nahlah Elkudssiah Ismail, Mihajlo Jakovljevic, Chinmay T Jani, Nitin Joseph, Vidya Kadashetti, Himal Kandel, Tesfaye K Kanko, Himanshu Khajuria, Md Jobair Khan, Moien AB Khan, Javad Khanali, Moawiah Mohammad Khatatbeh, Jagdish Khubchandani, Adnan Kisa, Farzad Kompani, Deborah R Kopansky-Giles, Ai Koyanagi, Kewal Krishan, Mohammed Kuddus, Narinder Kumar, Bagher Larijani, Justin Lo, Vanessa Sintra Machado, Preetam Bhalchandra Mahajan, Elaheh Malakan Rad, Ahmad Azam Malik, Lyn M March, Elezebeth Mathews, José João Mendes, Alexios-Fotios A Mentis, Mohamed Kamal Mesregah, Tomislav Mestrovic, Awoke Misganaw, Shafiu Mohammed, Ali H Mokdad, Md Moniruzzaman, Christopher J L Murray, Ghulam Mustafa, Mohsen Naghavi, Zuhair S Natto, Biswa Prakash Nayak, Cuong Tat Nguyen, Robina Khan Niazi, Ogochukwu Janet Nzoputam, In-Hwan Oh, Osaretin Christabel Okonji, Kanyin Liane Ong, Mayowa O Owolabi, Kevin Pacheco-Barrios, Jagadish Rao Padubidri, Jay Patel, Shrikant Pawar, Paolo Pedersini, Arokiasamy Perianayagam, Ionela-Roxana Petcu, Amir Radfar, Mohammad Hifz Ur Rahman, Vahid Rahmanian, Vahid Rashedi, Zubair Ahmed Ratan, Salman Rawaf, Elrashdy Moustafa Mohamed Redwan, Andre M N Renzaho, Nazila Rezaei, Nima Rezaei, Abanoub Riad, Aly M A Saad, Basema Saddik, Umar Saeed, Maryam Sahebazzamani, Amirhossein Sahebkar, Harihar Sahoo, Amir Salek Farrokhi, Allen Seylani, Saeed Shahabi, Bereket Beyene Shashamo, Jeevan K Shetty, Mika Shigematsu, Velizar Shivarov, Parnian Shobeiri, Migbar Mekonnen Sibhat, Jasvinder A Singh, Paramdeep Singh, Anna Aleksandrovna Skryabina, Helen Slater, Amanda E Smith, Yonatan Solomon, Jaimie D Steinmetz, Ker-Kan Tan, Nathan Y Tat, Arash Tehrani-Banihashemi, Samar Tharwat, Marcos Roberto Tovani-Palone, Biruk Shalmeno Tusa, Sahel Valadan Tahbaz, Stein Emil Vollset, Theo Vos, Anthony D Woolf, Ai-Min Wu, Sisay Shewasinad Yehualashet, Naohiro Yonemoto, Iman Zare, Armin Zarrintan, Chenwen Zhong, and Mohammad Zoladl.

Managing the estimation or publications process

Judie Arulappan, Marita Cross, Fikadu Nugusu Dessalegn, Hailey Hagins, Simon I Hay, Ali H Mokdad, Christopher J L Murray, Mohsen Naghavi, Kanyin Liane Ong, Nima Rezaei, Aly M A Saad, Jaimie D Steinmetz, and Theo Vos.
